# Supplementary material for: The relative contribution of close-proximity contacts, shared classroom exposure and indoor air quality to respiratory virus transmission in schools
Source: medRxiv. 2025 Aug 21:2025.08.18.25333891. Preprint. [Version 1] doi: 10.1101/2025.08.18.25333891 (PMC12393643; doi:10.1101/2025.08.18.25333891)
Supplement: Supplement 1 [file media-1.pdf]

Supplementary Information

A Genomic analysis 2

A.1 Supplementary methods 2

A.2 Supplementary results 3

B Pairwise survival analysis 8

B.1 Supplementary data 8

B.2 Supplementary results 9

B.3 Supplementary illustrations for methods 15

C Genomic sequence accession codes 18

C.1 Accession Codes for sequences used as targets in sequencing protocol 18

C.2 Accession codes for sequences from the Swiss Respiratory Virus Sequencing study 21

C.3 Accession codes for sequences from the Swiss Respiratory Virus Sequencing study 26

References 117

A Genomic analysis

A.1 Supplementary methods

Viral genomes from specimens testing positive for Influenza A (IAV), Influenza B (IBV), or respiratory syncytial virus (RSV) were sequenced using a tiled PCR amplicon-based Illumina™ sequencing-by-synthesis (SBS) workflow. Complementary DNA (cDNA) was generated from viral RNA and subsequently amplified using the xGen™ Respiratory Virus Amplicon Panel (IDT), which employs targeted primers for comprehensive genome coverage. The resulting amplicon libraries were prepared following standard Illumina library preparation protocols and sequenced on an Illumina NovaSeq X system. Resulting reads were aligned using BLAST to reference genomes for RSV A, RSV B, and IAV H1N1 and H3N2 and IBV victoria; accession numbers are detailed below. Consensus sequences were established for all specimens that had at least 20% coverage with depth of one read. For each specimen we generated three consensus sequences, masking sites with depth lower than 5, 10 and 20 reads.

We analysed the consensus sequences to establish likely transmission pairs and to evaluate the likely number of imported cases to best explain the diversity of IAV/IBV and RSV virus sampled from the school population. After sequencing it was clear that the general coverage of the consensus sequences was generally poor, but markedly better in the NA region of the IAV genome and the G region of the RSV genome Figure S1. For this reason, we used these sections exclusively for our analysis.

To establish likely transmission events, we compared every pair of sequences by quantifying the number of single nucleotide polymorphisms (SNPs) between sequences as a proportion of comparable sites (sites covered by the consensus in both sequences). Further, we calculated the likelihood that the viral RNA sequenced from each pair of hosts was consistent with a transmission event between them using Jukes-Cantor 1969 (JC69) model [1], assuming an evolution time of the number of days between test dates plus a perceived maximum of 4 days (2 days of within-host evolution per sample), and using the closed form [2]

$$\log \mathcal{L}(\mu, t \mid s_i, s_j) = l(s_i, s_j) \log\left(\frac{1}{4} + \frac{3}{4}e^{-\frac{4}{3}\mu t}\right) + d(s_i, s_j) \log\left(\frac{1}{4} - \frac{1}{4}e^{-\frac{4}{3}\mu t}\right),$$

where μ is the substitution rate, t is the time between samples in evolutionary time. For both, we assumed a nucleotide substitution rate of 1.5×10^{-3} mutations per site per year, which is close to the estimates in literature [3,4].

Further, to evaluate the relationship between sequences in a more holistic sense, we constructed maximum likelihood phylogenetic trees using a coalescent model using the *Nextstrain* framework [5]. Alongside the sequences acquired through the school study, we also included sequences from clinical samples across Switzerland [6] and publicly available sequences from GenBank (see the list of accession codes below).

A.2 Supplementary results

Consensus sequences were successfully defined for 10/13 samples testing positive for IAV, 3/6 for IBV (and 8/11 for RSV. The coverage of the consensus sequences was variable with 16–93% for IAV, 7–96% for IV and 10–86% for RSV at a read depth threshold of 10 (Figure S1). Due to the low number and relatively poor quality of IBV sequences, we chose not to analyse them in detail. Because IAV has a segmented genome, recombination of the segments makes comparison for the whole genomes challenging. Typically, the HA and NA genes are used for phylogenetic comparisons. Since in our case the HA gene was poorly covered, we focussed on the NA gene. For RSV, we focused on the G gene, which had consistently high coverage (site 4637 to 5631). Comparing the pairs of IAV consensus sequences, we found between 0 and 25 substitutions corresponding up to 0.025 substitutions per comparable pair of bases; the generation-based and JC69 log-likelihood values agreed well and resulted in 15 possible transmission pairs. For RSV, between 0 and 233 substitutions were present between sequences corresponding to up to 0.42 substitutions per comparable site. The JC69 and generation-based likelihoods were sufficiently comparable to identify the same 9 possible transition pairs. Pairwise comparisons for IAV and RSV sequences at read depths 5, 10, and 20 are shown in Figures S2–S7.

The IAV NA sequences were clustered on the maximum likelihood phylogenetic tree (Figure S8), suggesting that the viruses are all relatively closely related compared to the global diversity. The cluster was in a part of the tree amongst many other sequences from Switzerland; however, the location of this cluster is likely to be highly uncertain due to the relative genetic divergence from the rest of the tree. Moreover, the genetic divergences within the sub-clade are relatively large and mostly inconsistent with direct transmission between hosts. This structure partly arises because although five sequences are identical in overlapping sections (S-7, K-32, K-39, K-95 and K-75), they diverge on the tree as a subset (K-32 and K-95) are identical to S-7, where the others diverge. This introduces uncertainty into the precise local structure of the tree. The RSV G gene tree reveals four samples clustered together are genetically identical when overlapping sections are compared on a pairwise basis, and two that are distantly related. Of the four that are closely related, three (K-10, K-38 and P-67) are clustered together on the tree, with no divergence separating them. Sample P-79 has been placed in a separate clade on the tree despite being identical in the pairwise comparison, due to its relative proximity to sample K-75. The additional insights provided by the tree serve to highlight some dependency when assigning transmission pairs, however, precise groupings of samples is not possible with the information available.

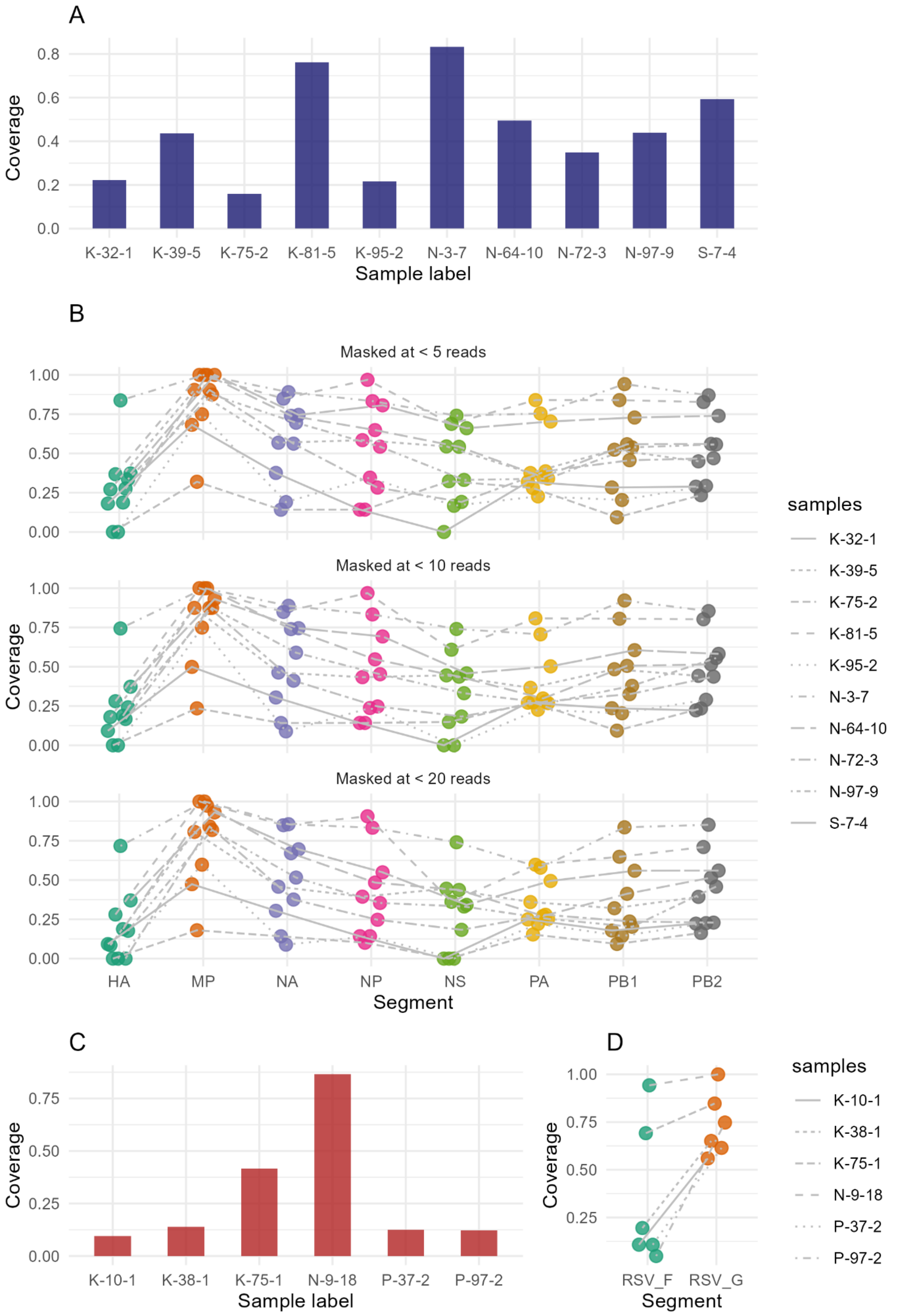
A
Coverage
0.0
0.2
0.4
0.6
0.8
K-32-1
K-39-5
K-75-2
K-81-5
K-95-2
N-3-7
N-64-10
N-72-3
N-97-9
S-7-4
Sample label
B
Masked at < 5 reads
Masked at < 10 reads
Masked at < 20 reads
Coverage
0.00
0.25
0.50
0.75
1.00
HA
MP
NA
NP
NS
PA
PB1
PB2
Segment
samples
K-32-1
K-39-5
K-75-2
K-81-5
K-95-2
N-3-7
N-64-10
N-72-3
N-97-9
S-7-4
C
Coverage
0.00
0.25
0.50
0.75
K-10-1
K-38-1
K-75-1
N-9-18
P-37-2
P-97-2
Sample label
D
Coverage
0.25
0.50
0.75
1.00
RSV_F
RSV_G
Segment
samples
K-10-1
K-38-1
K-75-1
N-9-18
P-37-2
P-97-2

Figure S1: Coverage of the consensus sequences. (A) Coverage of the whole influenza A H1N1 genome for each sample masked at read depth of 10. (B) Coverage for each segment of the influenza A H1N1 masked at different read depth thresholds (5, 10 and 20 reads). (C) Coverage of the whole genome of respiratory syncytial virus A for each sample masked at read depth of 10. D) Coverage for the F and G genes of RSV A masked a read depth threshold of 10.

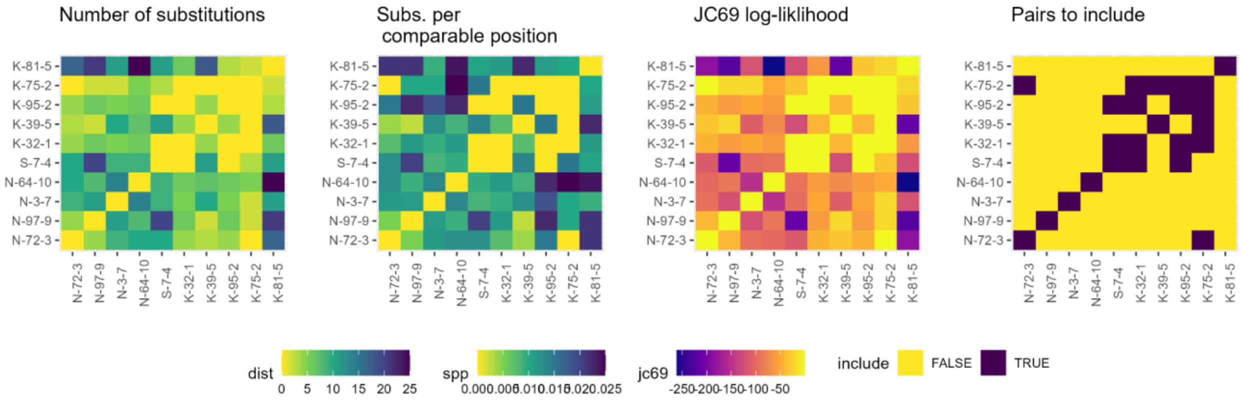

Figure S2: Pairwise comparison of the consensus sequences for the NA gene of influenza A virus H1N1, with masking at read depth of 5. The panels show the number of single nucleotide polymorphisms (SNPs), SNPs per nucleotide shared between sequences, the likelihood that pairs are consistent with a transmission event based on the Jukes-Cantor 1969 model and the pairs that were selected to be included or removed from the pairwise analysis.

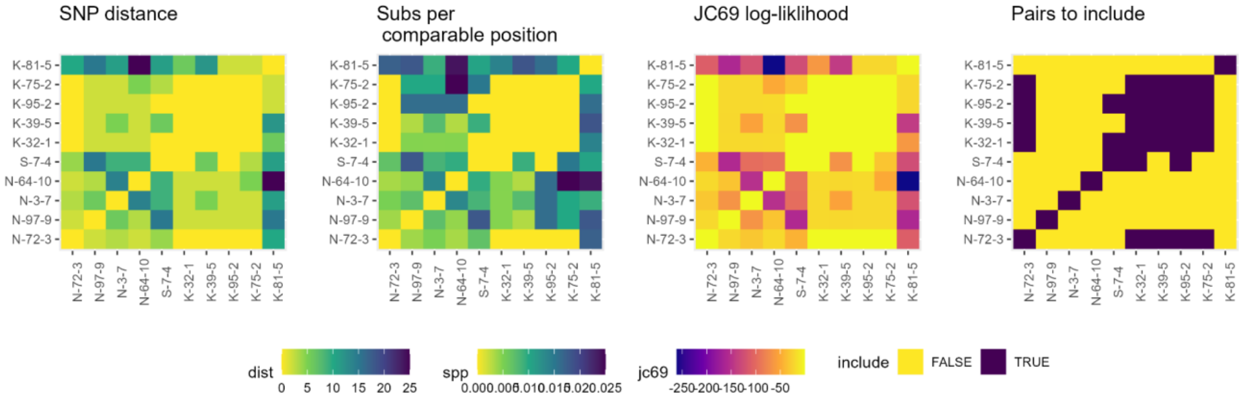

Figure S3: Pairwise comparison of the consensus sequences for the NA gene of influenza A virus H1N1, with masking at read depth of 10. The panels show the number of single nucleotide polymorphisms (SNPs), SNPs per nucleotide shared between sequences, the likelihood that pairs are consistent with a transmission event based on the Jukes-Cantor 1969 model and the pairs that were selected to be included or removed from the pairwise analysis.

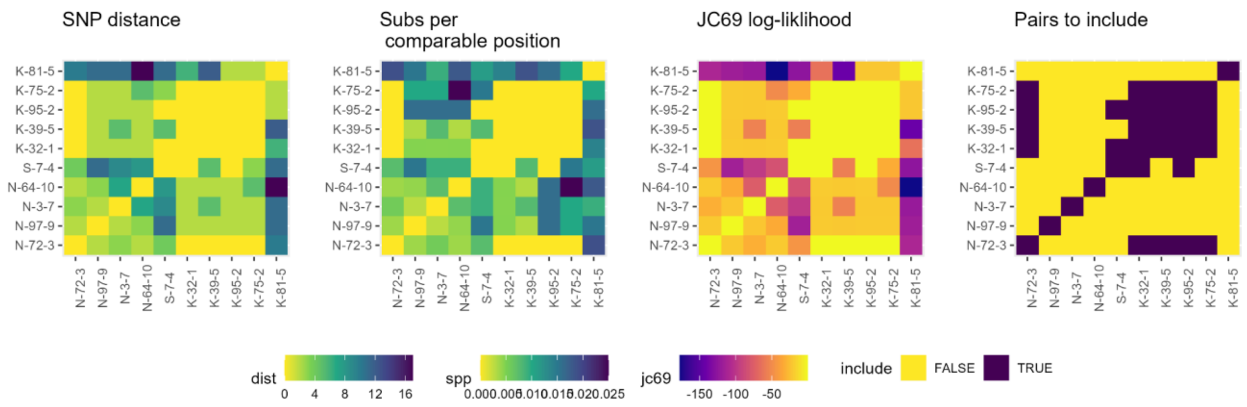

Figure S4: Pairwise comparison of the consensus sequences for the NA gene of influenza A virus H1N1, with masking at read depth of 20. The panels show the number of single nucleotide polymorphisms (SNPs), SNPs per nucleotide shared between sequences, the likelihood that pairs are consistent with a transmission event based on the Jukes-Cantor 1969 model and the pairs that were selected to be included or removed from the pairwise analysis.

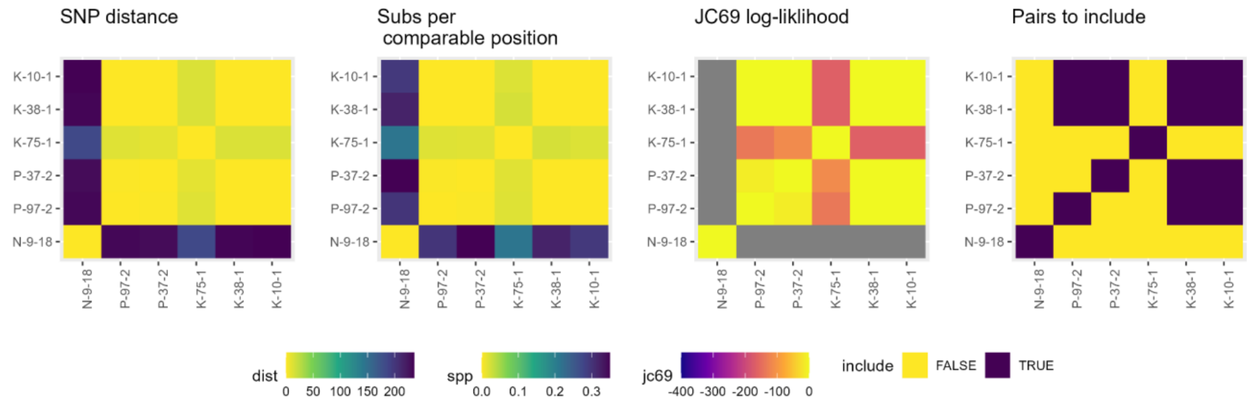

Figure S5: Pairwise comparison of the consensus sequences for the G gene of gene respiratory syncytial virus, with masking at read depth of 5. The panels show the number of single nucleotide polymorphisms (SNPs), SNPs per nucleotide shared between sequences, the likelihood that pairs are consistent with a transmission event based on the Jukes-Cantor 1969 model and the pairs that were selected to be included or removed from the pairwise analysis.

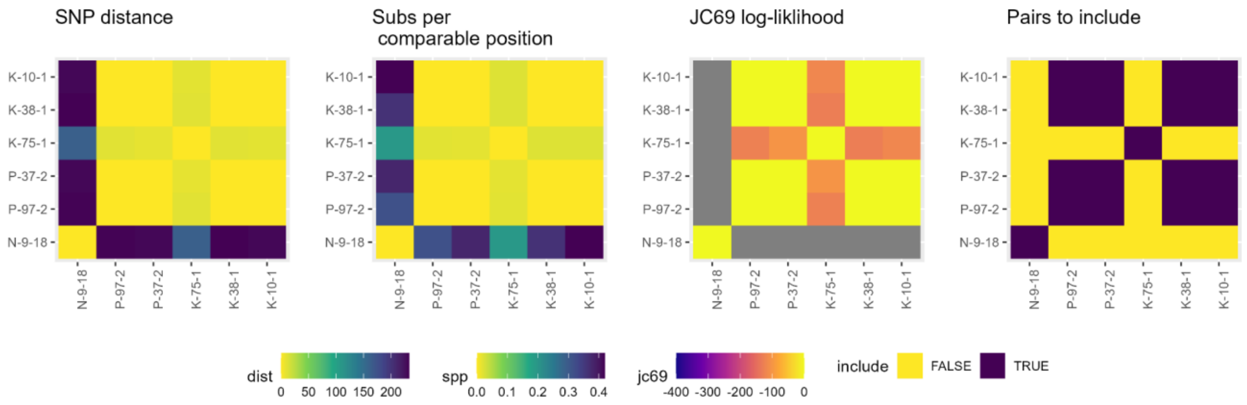

Figure S6: Pairwise comparison of the consensus sequences for the G gene of respiratory syncytial virus, with masking at read depth of 10. The panels show the number of single nucleotide polymorphisms (SNPs), SNPs per nucleotide shared between sequences, the likelihood that pairs are consistent with a transmission event based on the Jukes-Cantor 1969 model and the pairs that were selected to be included or removed from the pairwise analysis.

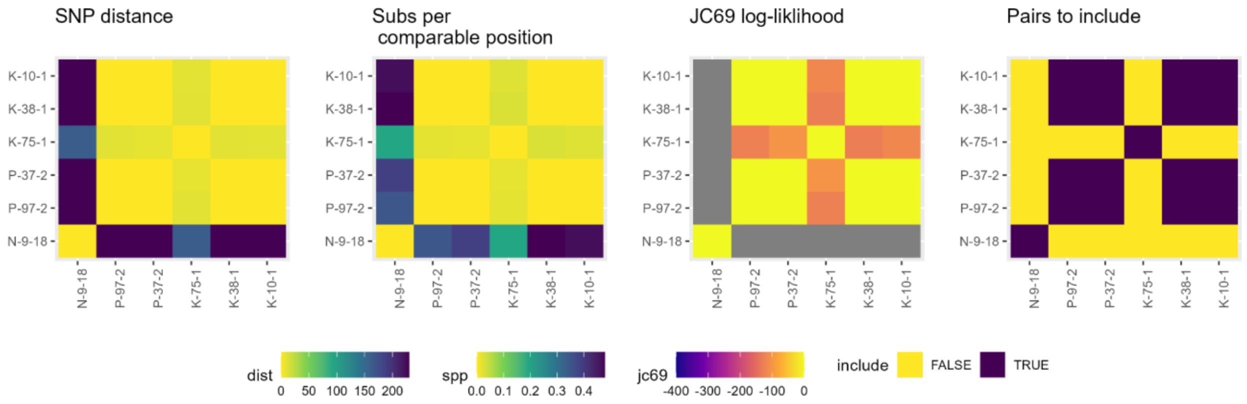

Figure S7: Pairwise comparison of the consensus sequences for the G of gene respiratory syncytial virus, with masking at read depth of 20. The panels show the number of single nucleotide polymorphisms (SNPs), SNPs per nucleotide shared between sequences, the likelihood that pairs are consistent with a transmission event based on the Jukes-Cantor 1969 model and the pairs that were selected to be included or removed from the pairwise analysis.

B Pairwise survival analysis

B.1 Supplementary data

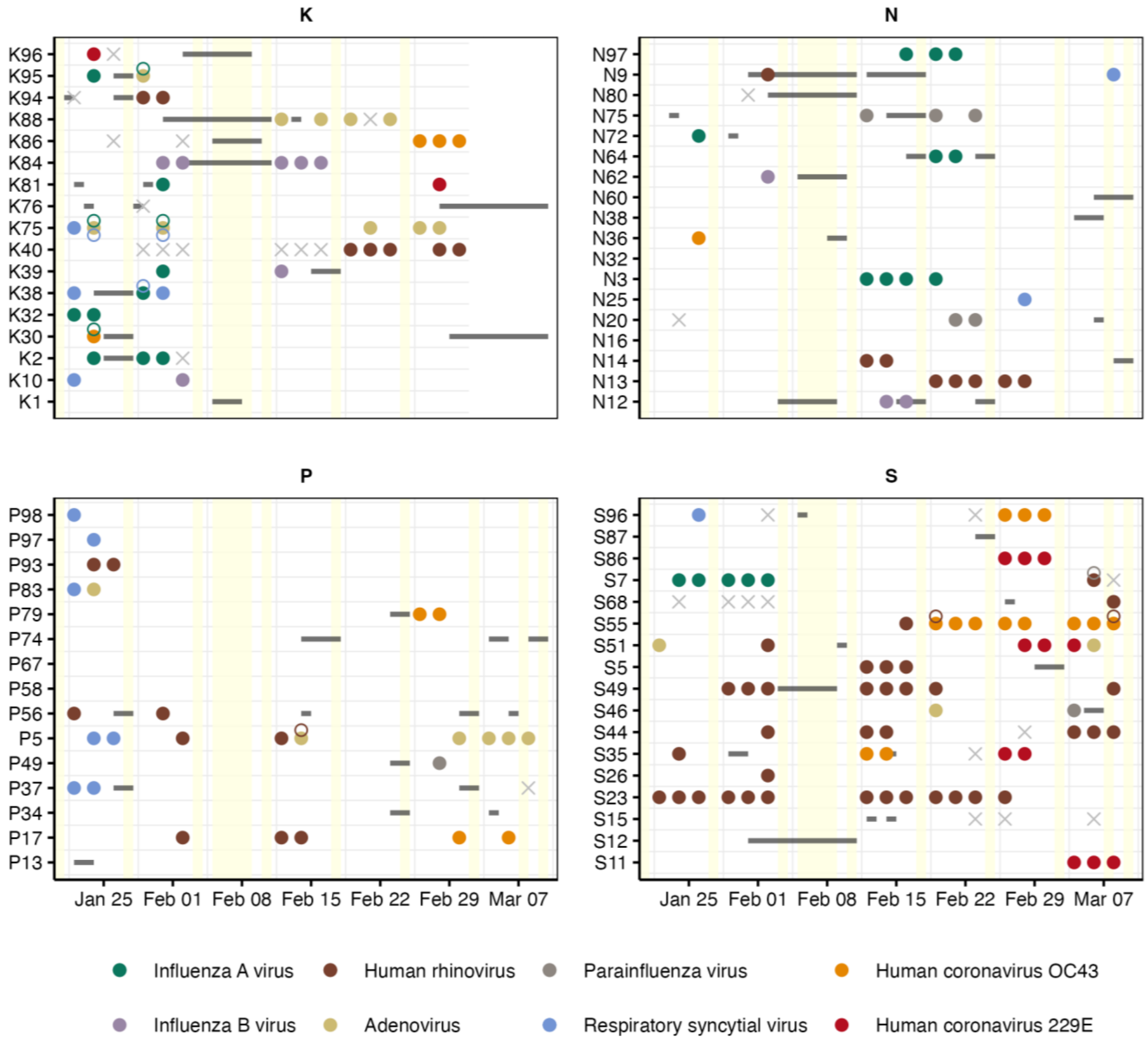

Figure S8: Dates of positive saliva test results (coloured dots) and absences from school (grey lines). Crosses show dates when students did not participate in saliva sampling. Yellow areas mark school-free days.

B.2 Supplementary results

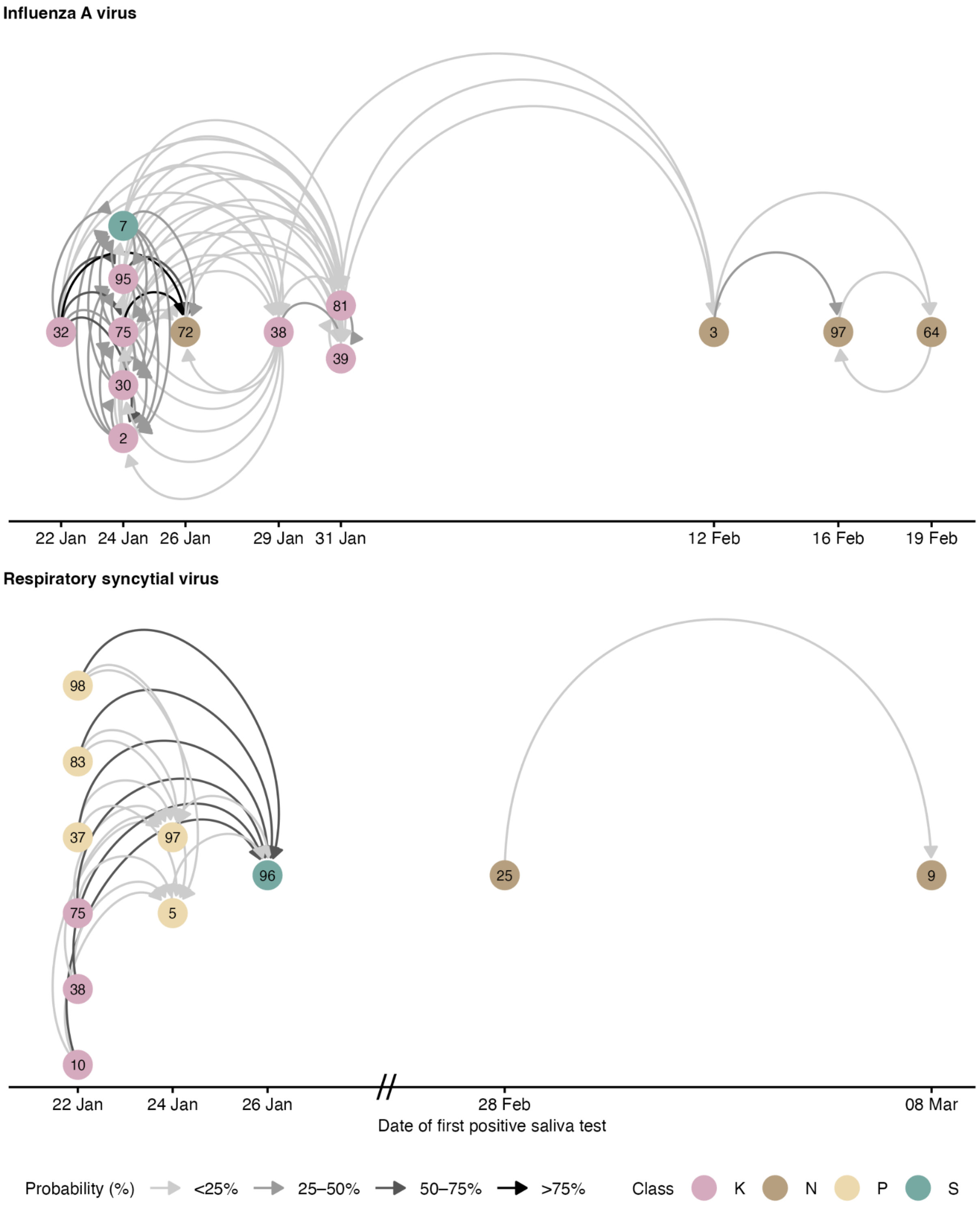

Figure S9: Transmission networks of influenza A and respiratory syncytial virus without excluding implausible links through genomic analysis. The probability is the proportion of paired datasets in which the link was present, considering uncertainty in pathogen-specific incubation and infectious periods. The temporal axis shows the date of the first positive saliva sample, which can be several days after the date of infection, so that circular links are possible.

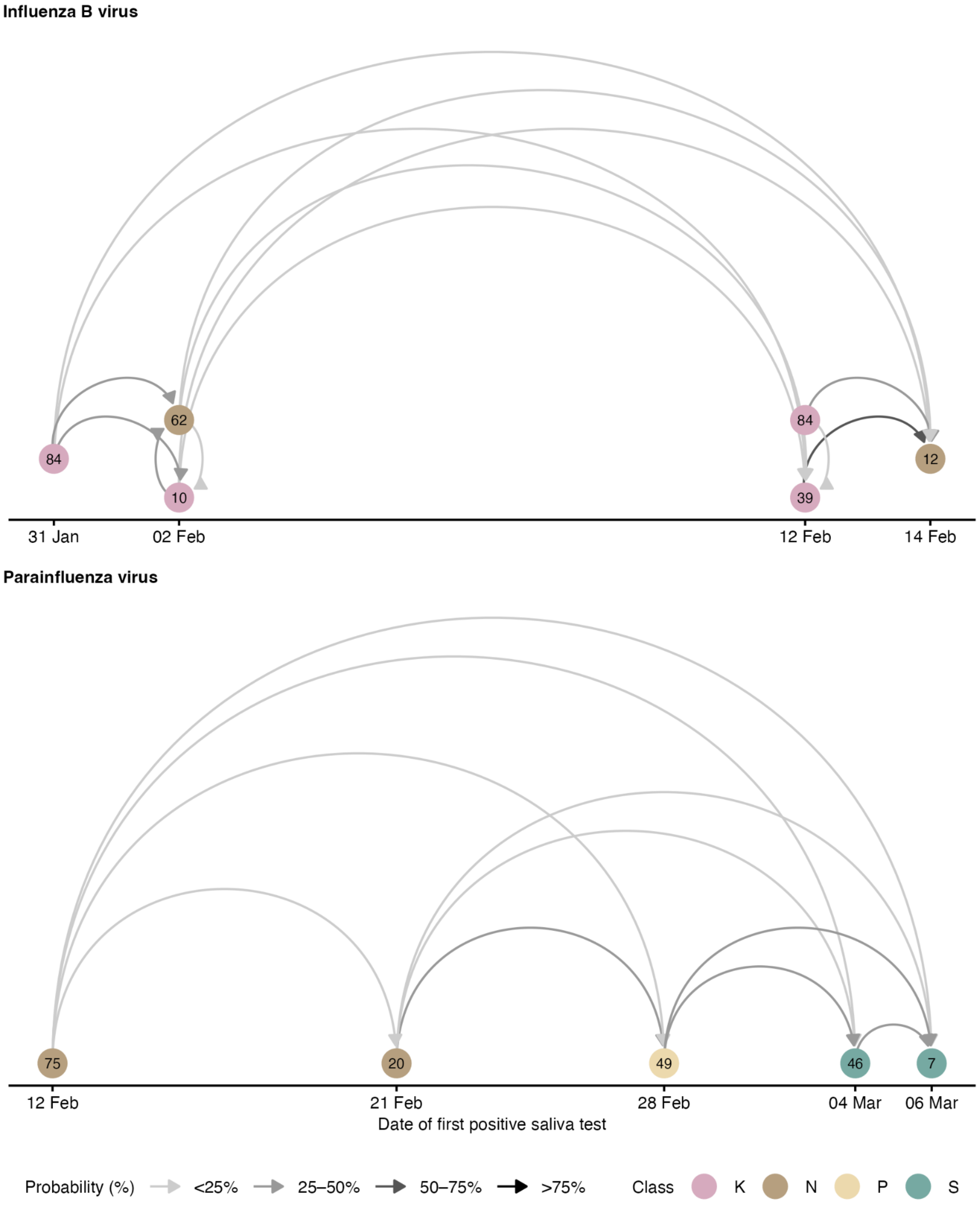

Figure S10: Transmission networks of influenza B and parainfluenza virus. The probability is the proportion of paired datasets in which the link was present, considering uncertainty in pathogen-specific incubation and infectious periods. The temporal axis shows the date of the first positive saliva sample, which can be several days after the date of infection, so that circular links are possible.

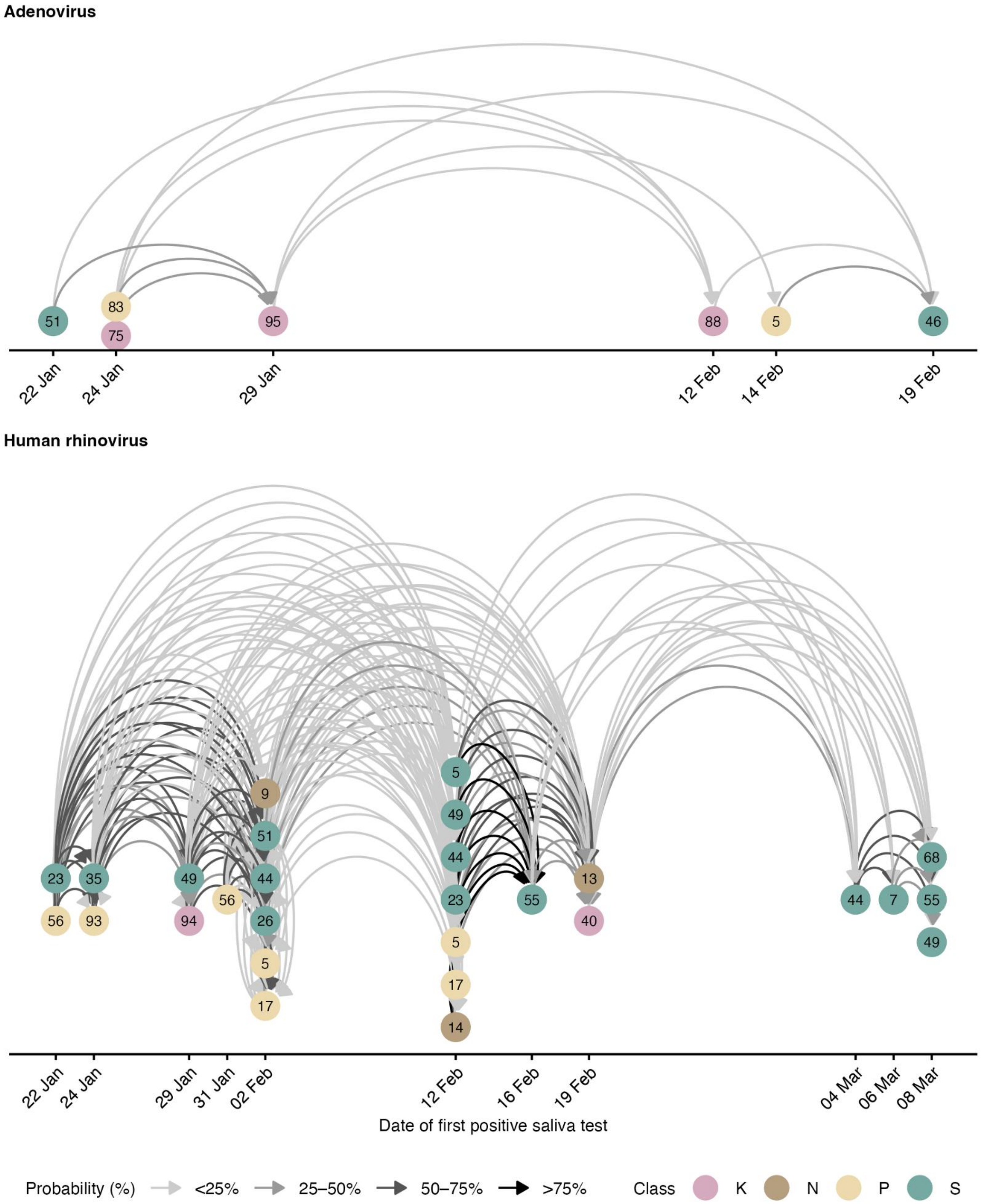

Figure S11: Transmission networks of human rhino and adenovirus. The probability is the proportion of paired datasets in which the link was present, considering uncertainty in pathogen-specific incubation and infectious periods. The temporal axis shows the date of the first positive saliva sample, which can be several days after the date of infection, so that circular links are possible.

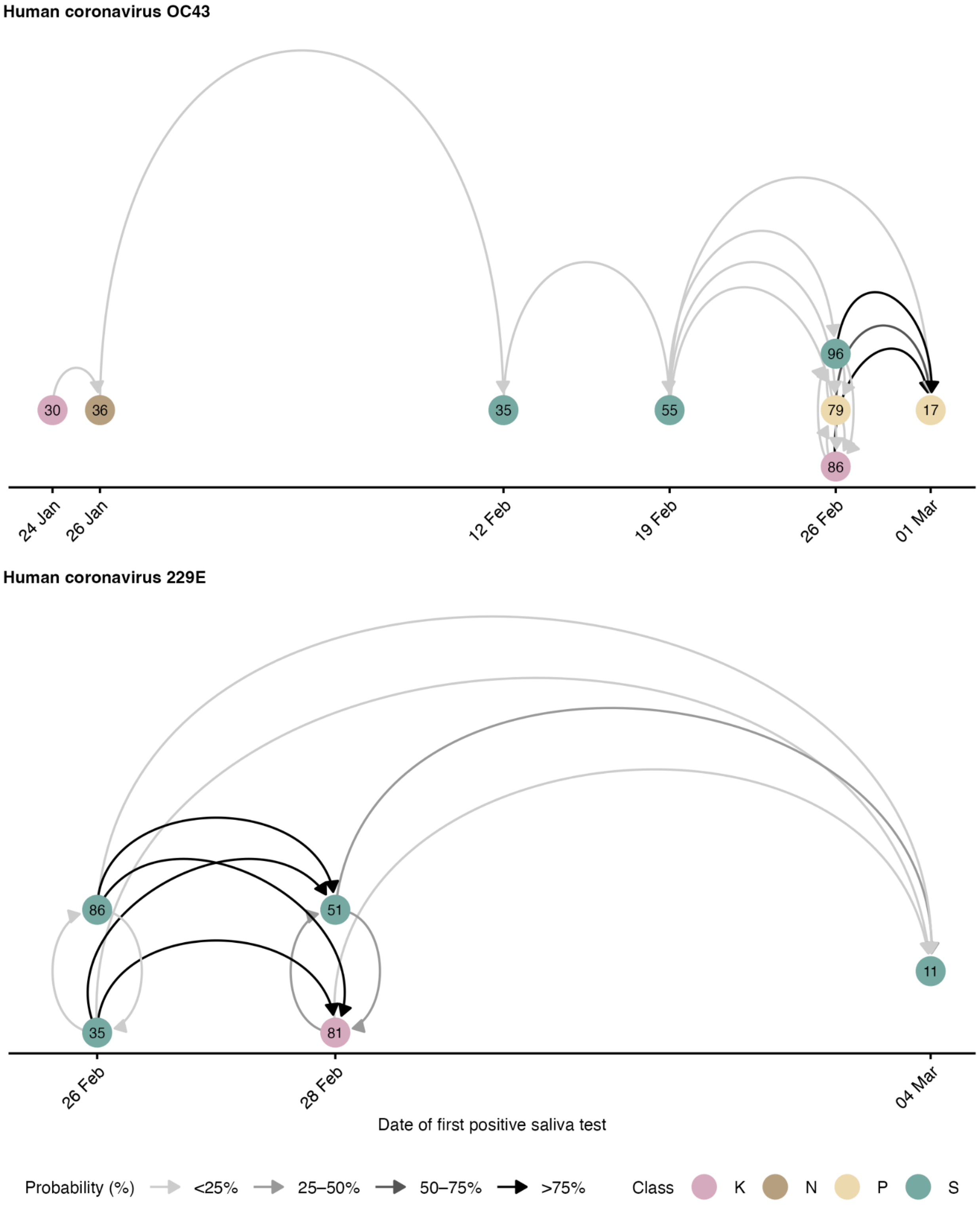

Figure S12: Transmission networks of human coronaviruses HCoV-OC43 and HCoV-229E. The probability is the proportion of paired datasets in which the link was present, considering uncertainty in pathogen-specific incubation and infectious periods. The temporal axis shows the date of the first positive saliva sample, which can be several days after the date of infection, so that circular links are possible.

Table S1: Rate ratios (median of the mean and 95%-confidence interval [CI] across paired datasets) of transmission risk factors from univariable models considering all transmissions within and between classes and only transmissions within classes. For time in close proximity, time in the shared classroom, and time with low indoor air quality, the rate ratio refers to a doubling in the time. The effects of time in the same classroom and time with low indoor air quality could not be assessed (n.a.) for all transmissions. The effect of being in the same class could not be assessed for transmissions only within classes. For social factors, we estimated the effects on internal and external transmission rates.

Variable	**All transmissions** Mean (95%-CI)	**Only within classes** Mean (95%-CI)
**Physical proximity**		–
Daily time in close proximity (log sec)		
Inf.-weighted average during exposure	1.16 (1.01–1.33)	1.09 (0.84–1.41)
Unweighted average during exposure	1.07 (0.90–1.27)	0.85 (0.69–1.06)
Overall median during exposure	1.12 (0.94–1.34)	0.95 (0.79–1.14)
**Shared classroom**		
Students in the same class	4.02 (1.69–9.64)	n.a.
Time in the same classroom (log min)	n.a.	8.96 (4.85–16.88)
**Indoor air quality**		
Daily time with low indoor air quality (log min)		
CO_2 >1,000 ppm in classroom	n.a.	5.59 (3.25 – 9.83)
CO_2 >1,400 ppm in classroom	n.a.	2.62 (1.53 – 4.46)
$PM_{2.5}$ > 5 µg/m³ in classroom	n.a.	2.83 (1.49 – 5.24)
**Social factors**		
Susceptible has siblings (internal)	1.81 (0.38–7.57)	1.15 (0.22–4.94)
Susceptible has extracurricular activities (internal)	1.69 (0.73–3.89)	1.03 (0.38–2.83)
Susceptible has siblings (external)	0.36 (0.06–2.44)	0.80 (0.14–5.51)
Susceptible has extracurricular activities (external)	0.55 (0.19–1.61)	1.05 (0.32–3.41)

Notes: Time in close proximity in the main analysis was measured by the daily infectiousness-weighted average time during the exposure period and indoor air quality by the daily time CO_2 *> 1,000 ppm in the classroom. Other measures were considered as part of a sensitivity analysis. Further note that* $PM_{2.5}$ *concentration (µg/m³) was only measured in the rooms of classes K and S.*

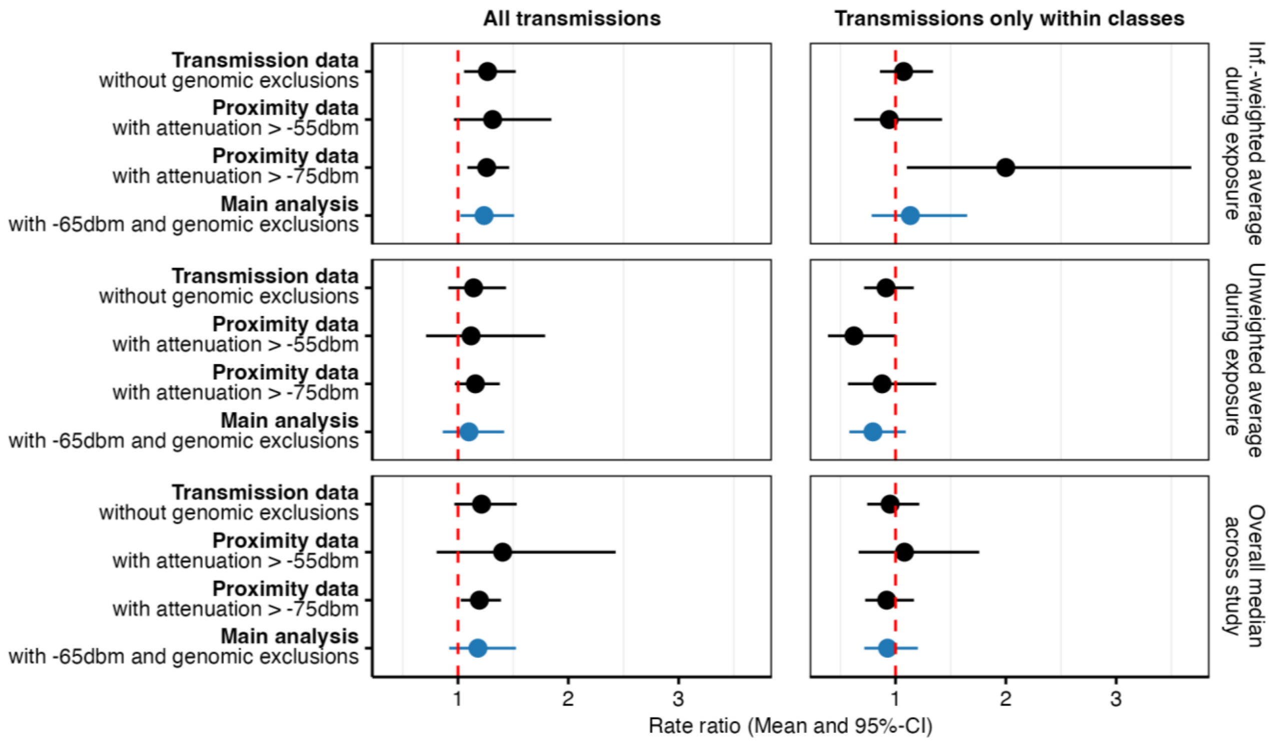

Figure S13: Sensitivity analysis showing the rate ratio (means as dots and 95%-confidence intervals [CIs] as lines) for the effects of close-proximity time on transmission using data without genomic exclusions for influenza A and respiratory syncytial virus and with different attenuation thresholds for detecting close-proximity contacts with the wearable sensors. Main analysis highlighted in blue on data with genomic exclusions and an attenuation threshold of -65dbm.

B.3 Supplementary illustrations for methods

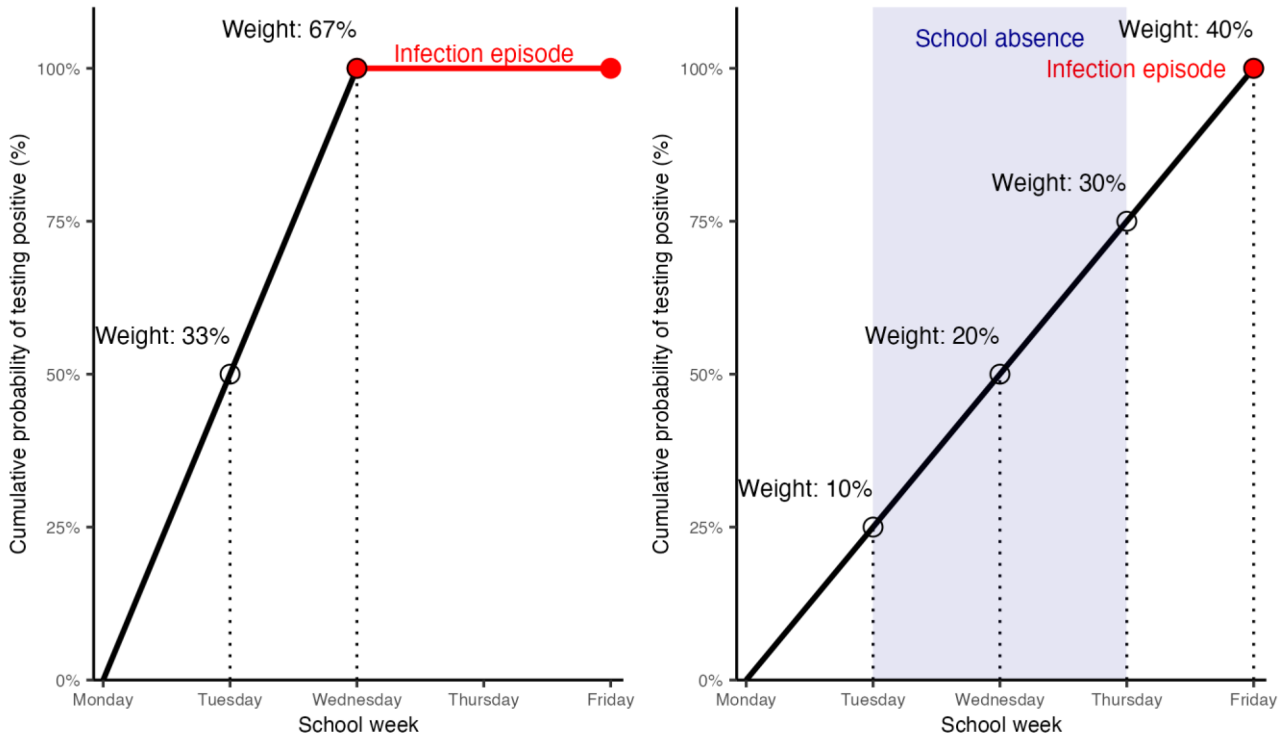

Figure S14: Illustration of probabilistic sampling for the first possible positive saliva test result (start of infectiousness). Left: Student first tested positive on Wednesday with first possible date on Tuesday, corresponding to sampling weights of 33% for Tuesday and 67% for Wednesday. Right: Student first tested positive on Friday but was absent from school from Tuesday to Thursday, therefore the weights are 10%, 20%, 30%, and 40% for Tuesday to Friday, respectively.

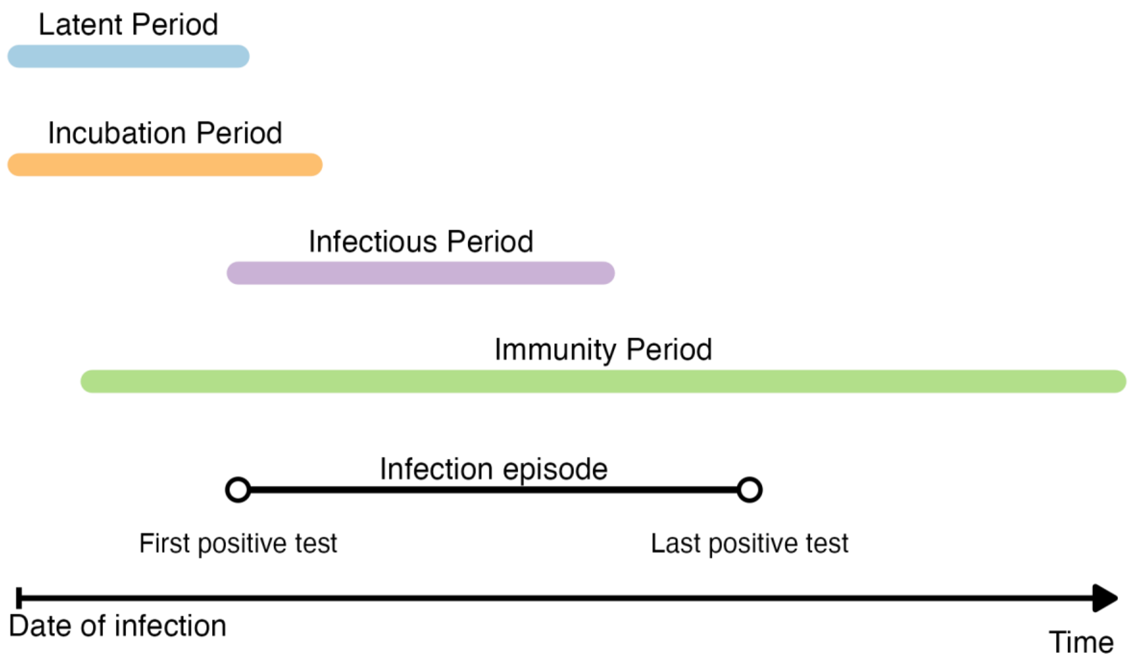

Figure S15: Illustration of the incubation, latent, infectious, and immunity period as derived from infection episodes defined by a period of consecutive positive saliva test results.

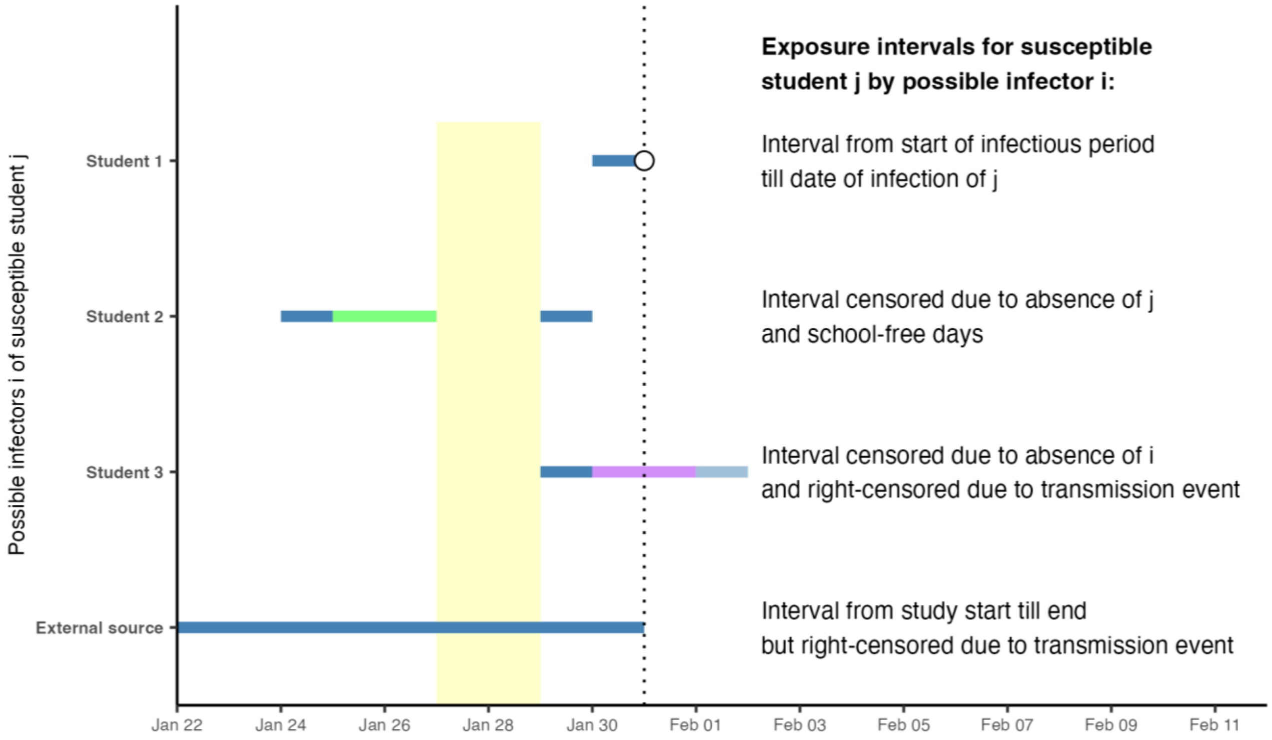

Figure S16: Illustration of different types of exposure intervals (blue lines) for a susceptible student j with varying infectors i. Date of infection shown as dot, weekend as yellow area, absence period of susceptible student j as green line and of infectious student i as purple line, right-censored exposure period as more transparent blue-coloured line. Note that the exposure interval is generally defined as the time the susceptible student is in school together with the infectious student. School-free days and absences can shorten the exposure interval, except for external sources to which the susceptible student is always considered exposed. Right-censoring can occur due prior infection by another student.

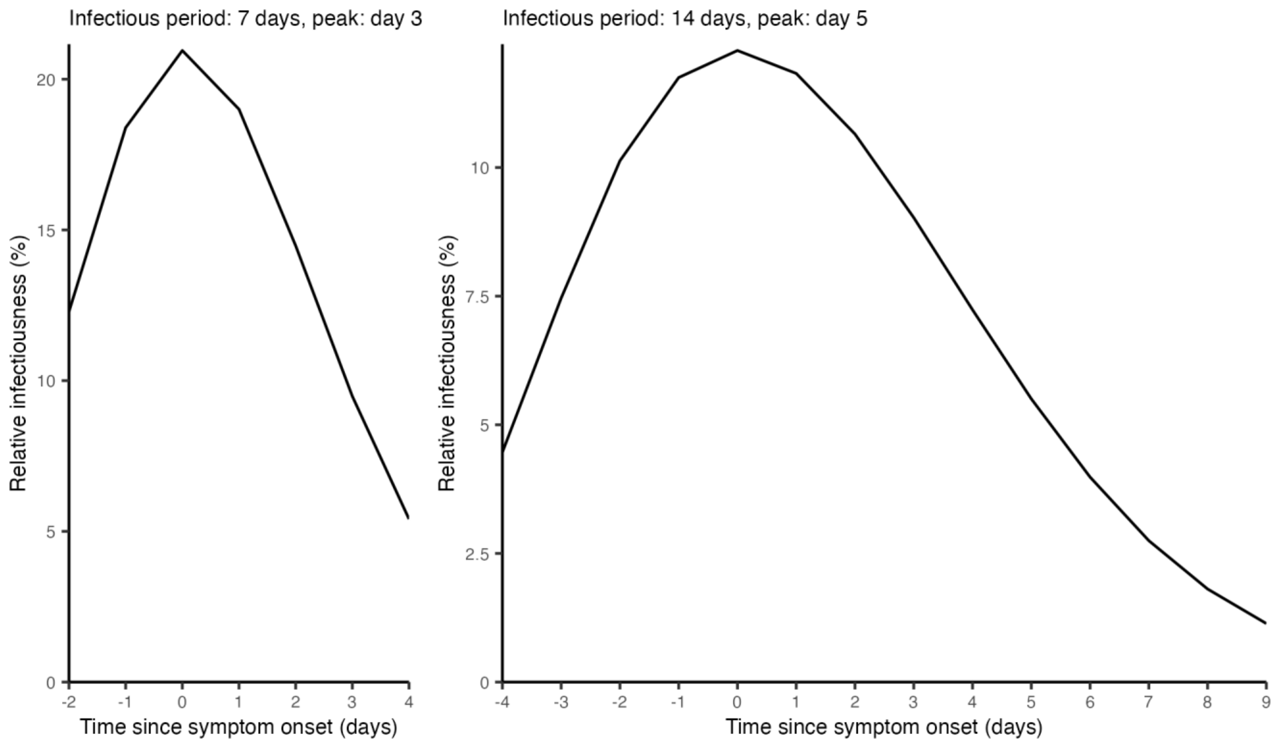

Figure S17: Illustration of the relative infectiousness for two infectious periods of seven and 14 days with a peak of infectiousness at days three and five, the days of symptom onset, respectively.

Table S2: Illustration of the impact of weighting time in close proximity by relative infectiousness. Shown are the daily average infectiousness-weighted times during the exposure period for three susceptible individuals A, B, and C. Each susceptible has a cumulative time of five minutes during the exposure period. Susceptible A has one minute on every day, B was not exposed (n.e.) on the first three days, while C was not exposed during the last three days. The infectious individual had an infectious period of five days with symptom onset (peak of infectiousness) on the second day.

	**Close contact times during infectious period**					**Total and average time during exposure period**		
**Susceptible**	Day 1	Day 2 (peak)	Day 3	Day 4	Day 5	Total	Unweighted average	Infectiousness-weighted average
**A**	1	1	1	1	1	5	1	1
**B**	n.e.	n.e.	n.e.	2	3	5	2.5	1.14
**C**	2	3	n.e.	n.e.	n.e.	5	2.5	3.53

C Genomic sequence accession codes

C.1 Accession Codes for sequences used as targets in sequencing protocol

IAV:

NC_007373.1-start: 1

NC_007373.1-end: 2341

NC_007372.1-start: 2342

NC_007372.1-end: 4682

NC_007371.1-start: 4683

NC_007371.1-end: 6915

NC_007366.1-start: 6916

NC_007366.1-end: 8677

NC_007369.1-start: 8678

NC_007369.1-end: 10243

NC_007368.1-start: 10244

NC_007368.1-end: 11710

NC_007367.1-start: 11711

NC_007367.1-end: 12737

NC_007372.1-start: 12738

NC_007372.1-end: 15078

IBV:

CY040455.1-start: 1

CY040455.1-end: 2315

CY040456.1-start: 2316

CY040456.1-end: 4675

CY040454.1-start: 4676

CY040454.1-end: 6927

CY040449.1-start: 6928

CY040449.1-end: 8754

CY040452.1-start: 8755

CY040452.1-end: 10530

CY040451.1-start: 10531

CY040451.1-end: 12015

CY040450.1-start: 12016

CY040450.1-end: 13159

CY040453.1-start: 13160

CY040453.1-end: 14196

<u>H1N1:</u>

NC_026431.1-start: 1

NC_026431.1-end: 982

NC_026432.1-start: 983

NC_026432.1-end: 1845

NC_026433.1-start: 1846

NC_026433.1-end: 3546

NC_026434.1-start: 3547

NC_026434.1-end: 4956

NC_026435.1-start: 4957

NC_026435.1-end: 7230

NC_026436.1-start: 7231

NC_026436.1-end: 8727

NC_026437.1-start: 8728

NC_026437.1-end: 10878

NC_026438.1-start: 10879

NC_026438.1-end: 13158

<u>SARS-CoV_2:</u>

NC_045512.2-start: 1

NC_045512.2-end: 29903

<u>RSV A:</u>

OP890336.1-start: 1

OP890336.1-end: 15261

<u>RSV B:</u>

OP965707.1-start: 1

OP965707.1-end: 15278

C.2 Accession codes for sequences from the Swiss Respiratory Virus Sequencing study

Table S3: Accession codes for the BioProject PRJEB83635, of genomic sequences from the Swiss Respiratory Virus Sequencing study used for the Influenza A NA phylogenetic tree

accession	date	country
xwkVbK	27.12.2023	Switzerland
kwbwf8	26.12.2023	Switzerland
wVBQbT	29.05.2024	Switzerland
3qDrEd	19.04.2023	Switzerland
h2ZJd5	31.01.2024	Switzerland
ydSpNQ	16.01.2024	Switzerland
qtZxkz	15.01.2024	Switzerland
Sm9GYA	12.01.2024	Switzerland
32WNFL	07.12.2023	Switzerland
g33df2	09.01.2024	Switzerland
gkcF5C	31.01.2024	Switzerland
xrRPFj	18.01.2024	Switzerland
PZQaiq	20.02.2024	Switzerland
3edUBd	30.11.2023	Switzerland
b99ooj	27.02.2024	Switzerland
Rtjhba	24.01.2024	Switzerland
VA9Yi5	10.01.2024	Switzerland
qeHUBn	11.04.2024	Switzerland
SQZYHn	17.01.2024	Switzerland
XEuJCy	22.12.2023	Switzerland
PGoRgN	20.02.2024	Switzerland
V5iihT	18.01.2024	Switzerland
QqAEGg	22.02.2024	Switzerland
gF3E5u	22.02.2024	Switzerland
SKJgwA	18.01.2024	Switzerland
FXFt6J	04.03.2024	Switzerland
gaQkyT	06.03.2024	Switzerland
YowhiU	16.02.2024	Switzerland
4SnyCD	10.04.2023	Switzerland
K2udH5	30.01.2024	Switzerland
xHGR9u	17.01.2024	Switzerland
jtjCjd	27.12.2023	Switzerland
iDqyV9	25.01.2024	Switzerland
HtiJwT	19.02.2024	Switzerland

3H4uLZ	01.02.2024	Switzerland
375EUk	08.12.2023	Switzerland
mc3GSV	28.12.2023	Switzerland
C983Zw	19.02.2024	Switzerland
4UF9K6	28.12.2023	Switzerland
f8rfBe	04.03.2024	Switzerland
qWgDtq	04.06.2024	Switzerland
4L3pXZ	27.01.2024	Switzerland
WrGEQp	27.02.2024	Switzerland
YYRzxL	21.02.2024	Switzerland
8QXJTp	28.01.2024	Switzerland
UqhsDL	23.12.2023	Switzerland
hozdkG	15.02.2024	Switzerland
sFFUfJ	14.03.2024	Switzerland
itJLqU	29.12.2023	Switzerland
w9v3Qv	12.01.2024	Switzerland
oLXZ9a	17.01.2024	Switzerland
m6243p	17.01.2024	Switzerland
q2oXnd	09.01.2024	Switzerland
URVR7h	19.01.2024	Switzerland
MHWQwo	11.01.2024	Switzerland
opfXjQ	11.01.2024	Switzerland
9HhvMh	18.01.2024	Switzerland
x6NRYc	16.01.2024	Switzerland
LosGHn	28.01.2024	Switzerland
ZAyjrq	18.12.2023	Switzerland
39kc9U	19.01.2024	Switzerland
2b8YpQ	16.02.2024	Switzerland
4t7Ncr	31.01.2024	Switzerland
RyXauM	27.12.2023	Switzerland
9eyPAv	30.03.2024	Switzerland
ztFGoy	02.04.2024	Switzerland
juVwzj	01.02.2024	Switzerland
kBgpZ4	16.01.2024	Switzerland
SpXNmN	29.01.2024	Switzerland
3VC6ft	30.01.2024	Switzerland
jHbrsp	20.02.2024	Switzerland
yhE88h	01.03.2024	Switzerland
bpDXvz	26.04.2024	Switzerland

DwiwwU	19.01.2024	Switzerland
JFKsEJ	20.02.2024	Switzerland
kNU7js	15.02.2024	Switzerland
4rvG7f	19.01.2024	Switzerland
BnbHLr	26.01.2024	Switzerland
LCBdav	31.01.2024	Switzerland
sr4bvM	02.02.2024	Switzerland
6z2wf2	07.03.2024	Switzerland
uTpHe5	24.01.2024	Switzerland
AcEG8S	18.02.2024	Switzerland
uSTYZ2	04.03.2024	Switzerland
DgRuyg	14.02.2024	Switzerland
YBrMoY	16.02.2024	Switzerland
DMLKnU	15.02.2024	Switzerland
gyUNUP	19.02.2024	Switzerland
sAyJww	27.02.2024	Switzerland
2hKeW2	27.02.2024	Switzerland
j3ZXpL	29.02.2024	Switzerland
TPgies	11.03.2024	Switzerland
5cJWyf	13.03.2024	Switzerland
xYnaUy	16.01.2024	Switzerland
F2HifQ	18.01.2024	Switzerland
3p7s5a	20.03.2024	Switzerland
TPsF65	01.03.2024	Switzerland
CLwScw	28.12.2023	Switzerland
eAr6Lo	15.01.2024	Switzerland
ss4Fh8	19.01.2024	Switzerland
mmYEfJ	01.02.2024	Switzerland
fhoncL	02.02.2024	Switzerland
gsuEGK	16.02.2024	Switzerland
8ochGa	10.06.2024	Switzerland
FHPnrx	17.01.2024	Switzerland
hzNQRG	01.02.2024	Switzerland

Table S4: Accession codes for the BioProject PRJEB83635, of the genomic sequences from the Swiss Respiratory Virus Sequencing study used for the RSV G gene phylogenetic tree

accession	date	country
wF4k2T	07.03.2024	Switzerland
zGrf4n	20.11.2023	Switzerland
nf9ieP	24.01.2024	Switzerland
MT4r8p	20.12.2023	Switzerland
HXGr8V	28.12.2023	Switzerland
2MHu4K	13.03.2024	Switzerland
ahT4kF	29.01.2024	Switzerland
v3KDUF	27.12.2023	Switzerland
3oZJ5x	24.11.2023	Switzerland
NDEjHn	17.02.2024	Switzerland
iZ7TLc	12.03.2024	Switzerland
M5iqYC	30.01.2024	Switzerland
sVxWai	17.02.2024	Switzerland
CgiSAY	20.03.2024	Switzerland
9eyPAv	30.03.2024	Switzerland
FcN3wC	05.03.2024	Switzerland
Di2qm7	27.02.2024	Switzerland
xPAa2d	22.12.2023	Switzerland
XisgNp	27.12.2023	Switzerland
33eCP4	06.12.2023	Switzerland
afGSAT	04.03.2024	Switzerland
RCdbJH	21.02.2024	Switzerland
6RPWyT	06.03.2024	Switzerland
8YYVHP	24.11.2023	Switzerland
TCsq7r	02.02.2024	Switzerland
3fLBSP	24.11.2023	Switzerland
zwqknf	23.11.2023	Switzerland
Ki8Aa3	12.03.2024	Switzerland
GpvELC	19.01.2024	Switzerland
4SRYNi	21.11.2023	Switzerland
3eGjGj	07.12.2023	Switzerland
hHKDyB	15.02.2024	Switzerland
Kr6366	10.04.2024	Switzerland
qFQ9kJ	10.04.2024	Switzerland
Q8Ba9A	30.01.2024	Switzerland
gnDemF	27.12.2023	Switzerland

Zu3u7N	29.12.2023	Switzerland
yebiMQ	23.02.2024	Switzerland
mzwDVC	10.01.2024	Switzerland
AnXy9n	16.02.2024	Switzerland
GbMzJy	16.02.2024	Switzerland
5fSMj3	07.03.2024	Switzerland
DBKZHX	04.03.2024	Switzerland
Qf8cLC	22.02.2024	Switzerland
T5VXtD	30.01.2024	Switzerland
pzbDPq	29.02.2024	Switzerland
zxhtR4	29.01.2024	Switzerland
HAAUgG	27.12.2023	Switzerland
4JSa55	29.11.2023	Switzerland
4i2tZF	29.12.2023	Switzerland
k9vgiw	31.01.2024	Switzerland
HXHQAT	20.12.2023	Switzerland
G86emu	21.02.2024	Switzerland
8ERNNJ	07.12.2023	Switzerland
3fGJE8	30.11.2023	Switzerland
G8vc2N	02.02.2024	Switzerland
NmHGDa	29.12.2023	Switzerland
ND22rx	04.03.2024	Switzerland
SrvHnQ	22.02.2024	Switzerland

C.3 Accession codes for sequences from the Swiss Respiratory Virus Sequencing study

Table S5: Accession codes for GenBank, of the global genomic sequences used for the Influenza A NA phylogenetic tree

accession	date	country
GS_002EHP8.1	01.01.2009	USA
GS_00286TP.1	23.03.2010	Nicaragua
GS_0022ULT.1	12.01.2010	USA
GS_001YQFG.1	22.05.2009	Taiwan
GS_0022RTG.1	01.11.2009	South Korea
GS_0026P8F.1	01.10.2009	USA
GS_0040EYK.1	12.03.2010	USA
GS_003U2RP.1	28.01.2011	China
GS_003RQ88.1	01.08.2010	Taiwan
GS_0028PFX.1	26.01.2011	USA
GS_00255SY.1	07.12.2009	Poland
GS_003VFDZ.1	24.03.2010	Singapore
GS_001ZB87.1	23.06.2009	Argentina
GS_002EZRN.1	26.06.2009	Singapore
GS_002ES9U.1	07.07.2009	Singapore
GS_002CU0E.1	05.08.2009	Hong Kong
GS_00233VY.1	18.01.2010	USA
GS_0021EDQ.1	18.10.2009	USA
GS_00AAQFJ.1	01.01.2017	Colombia
GS_002F67G.1	11.01.2011	Singapore
GS_006S8LU.1	07.09.2011	Uganda
GS_0041B49.1	25.12.2010	USA
GS_002CQDS.1	27.01.2011	USA
GS_00585CJ.1	30.11.2010	United Kingdom
GS_0041VV8.1	20.03.2012	USA
GS_0054YPA.1	08.07.2012	Brazil
GS_002PNE3.1	21.09.2012	Thailand
GS_0041SH1.1	02.07.2012	USA
GS_003SBJY.1	25.01.2011	Russia
GS_002G9DZ.1	26.01.2011	Estonia
GS_0090MH3.1	01.02.2011	USA
GS_002G9HQ.1	15.02.2011	China
GS_0029GPK.1	27.08.2010	Thailand
GS_0041SLV.1	18.01.2012	USA

GS_0041298.1	07.03.2011	Iran
GS_004QZWP.1	28.09.2010	Singapore
GS_0041SW9.1	07.02.2012	USA
GS_0041VNN.1	09.04.2012	USA
GS_0041WSD.1	12.03.2012	USA
GS_0041SX7.1	09.02.2012	USA
GS_0041VQJ.1	02.03.2012	USA
GS_0045VWY.1	19.02.2013	Russia
GS_0045FVE.1	10.04.2013	USA
GS_00454XM.1	05.02.2013	Finland
GS_002RKRD.1	30.06.2013	Nicaragua
GS_005510J.1	21.08.2013	Brazil
GS_002RMD2.1	25.12.2013	USA
GS_004AXCP.1	20.11.2013	USA
GS_0040P5X.1	04.02.2014	USA
GS_0045H9K.1	08.08.2013	USA
GS_004RH5L.1	19.12.2013	USA
GS_004AQUX.1	04.02.2014	Finland
GS_004AR75.1	17.02.2014	Finland
GS_002RNZU.1	10.03.2014	USA
GS_002RPA6.1	09.04.2014	USA
GS_00484NZ.1	30.12.2013	USA
GS_0055JH0.1	08.03.2014	Japan
GS_0055JJY.1	29.01.2014	Japan
GS_002Z03T.1	25.11.2015	Nicaragua
GS_004SG5K.1	07.01.2015	USA
GS_0051CAV.1	19.03.2015	State of Palestine
GS_004UT0E.1	05.11.2015	USA
GS_004X4NL.1	04.10.2015	Kenya
GS_004USN4.1	07.09.2015	USA
GS_004ZVCC.1	11.04.2016	USA
GS_004WAS8.1	30.12.2015	USA
GS_002WYJ4.1	12.05.2017	USA
GS_002V6AF.1	05.01.2017	USA
GS_004W6TA.1	17.12.2015	USA
GS_002WXPV.1	26.02.2017	USA
GS_002VVTR.1	09.02.2017	USA
GS_002WJDV.1	27.02.2017	USA

GS_004ZVVB.1	29.04.2016	USA
GS_00513FU.1	20.01.2016	USA
GS_004XV6U.1	28.02.2016	USA
GS_004USZG.1	31.10.2015	USA
GS_004W7D4.1	25.11.2015	USA
GS_004XR50.1	18.01.2016	USA
GS_00515MD.1	08.02.2016	USA
GS_004XN61.1	12.02.2016	USA
GS_0054366.1	21.03.2016	USA
GS_004Y3HV.1	15.04.2016	USA
GS_002V64T.1	26.12.2016	USA
GS_002VWCM.1	24.02.2017	USA
GS_006CH93.1	01.02.2019	USA
GS_006FTLY.1	11.03.2019	USA
GS_007B8ZY.1	14.02.2020	USA
GS_002XGFR.1	16.07.2017	USA
GS_002YZH1.1	03.09.2017	USA
GS_006Z28B.1	30.09.2018	USA
GS_006LS9B.1	14.02.2019	USA
GS_00976K0.1	14.12.2018	USA
GS_0066QS7.1	07.01.2019	USA
GS_0069HHS.1	29.12.2018	USA
GS_006BM75.1	11.01.2019	USA
GS_005HXW4.1	21.02.2018	USA
GS_005P1DP.1	11.04.2018	USA
GS_005YL60.1	03.10.2018	USA
GS_00602T5.1	06.08.2018	USA
GS_005HWTB.1	23.02.2018	USA
GS_005LHG7.1	09.03.2018	USA
GS_002ZQK4.1	08.12.2017	USA
GS_00615VW.1	14.11.2018	USA
GS_006Y55G.1	15.06.2019	USA
GS_006BJZP.1	03.01.2019	South Korea
GS_0079B9E.1	21.03.2019	China
GS_007B7E4.1	25.01.2020	USA
GS_007EA95.1	05.10.2019	USA
GS_00AND4S.1	01.01.2020	USA
GS_007AXQV.1	03.02.2020	USA
GS_0077BE8.1	29.10.2019	USA

GS_0079PR3.1	08.01.2020	USA
GS_0087SJH.1	24.02.2021	USA
GS_00ANJW2.1	01.02.2020	USA
GS_007B6RG.1	23.01.2020	USA
GS_00ANE9F.1	12.01.2020	USA
GS_007AXCL.1	03.02.2020	USA
GS_007BNN6.1	03.03.2020	USA
GS_007NYSZ.1	18.09.2021	USA
GS_0089SL9.1	09.07.2021	USA
GS_007NYR1.1	22.09.2021	USA
GS_007SFT6.1	24.10.2021	USA
GS_008VQ35.1	21.08.2022	USA
GS_009V71R.1	26.10.2022	United Kingdom
GS_007P9LY.1	17.09.2021	USA
GS_007P9SL.1	17.09.2021	USA
GS_007R422.1	19.11.2021	USA
GS_007P9QQ.1	19.11.2021	USA
GS_007R430.1	19.11.2021	USA
GS_009Y9VU.1	10.10.2023	USA
GS_009XNEE.1	06.12.2023	USA
GS_009WL28.1	26.10.2023	USA
GS_009Z7KE.1	30.11.2023	USA
GS_009WTK0.1	30.10.2023	USA
GS_009Z2EW.1	08.11.2023	USA
GS_00A0BJA.1	06.09.2023	Thailand
GS_009XA2G.1	26.12.2023	USA
GS_00A389S.1	16.02.2024	USA
GS_00A528W.1	28.12.2023	USA
GS_009WMCM.1	02.12.2023	USA
GS_00A54AQ.1	21.12.2023	USA
GS_009ZPWA.1	29.12.2023	USA
GS_009ZADQ.1	24.10.2023	USA
GS_009Z6Q5.1	25.10.2023	USA
GS_009YPCF.1	26.12.2023	Germany
GS_00AH8KA.1	26.02.2024	USA
GS_008V9MH.1	21.11.2022	USA
GS_009ABD2.1	27.02.2023	USA
GS_009270E.1	16.12.2022	USA

GS_008ZHCL.1	19.12.2022	Germany
GS_00AVXBE.1	13.02.2023	USA
GS_00A9VPY.1	08.12.2023	USA
GS_008V001.1	11.10.2022	USA
GS_0091MZ3.1	09.01.2023	USA
GS_009SPP4.1	25.09.2023	Japan
GS_009SPQ2.1	01.10.2023	Japan
GS_009A67K.1	28.01.2023	USA
GS_008VSYC.1	31.10.2022	USA
GS_009ABHT.1	28.02.2023	USA
GS_009HYS7.1	05.08.2023	USA
GS_009P17X.1	03.08.2023	USA
GS_009P18V.1	03.08.2023	USA
GS_009QKT4.1	13.12.2022	USA
GS_009J3GM.1	24.06.2023	USA
GS_009ZBFK.1	19.10.2023	USA
GS_009VEES.1	27.06.2023	Australia
GS_009ZKDF.1	19.09.2023	USA
GS_009WUWB.1	26.11.2023	USA
GS_009X9R4.1	24.12.2023	USA
GS_009Z72F.1	03.10.2023	USA
GS_009ZCVP.1	23.11.2023	USA
GS_009Y694.1	14.12.2023	USA
GS_009J2FQ.1	30.07.2023	USA
GS_009EHM5.1	24.07.2023	USA
GS_009A4Y4.1	08.02.2023	USA
GS_009A96J.1	15.02.2023	USA
GS_00A0VJS.1	10.08.2023	USA
GS_00A103G.1	30.01.2024	United Kingdom
GS_009YAGL.1	08.11.2023	USA
GS_00A5145.1	28.12.2023	USA
GS_009DK54.1	02.06.2023	USA
GS_009J3VU.1	30.06.2023	USA
GS_00A3FKY.1	31.12.2023	USA
GS_0096ESE.1	07.12.2022	USA
GS_009HJNV.1	04.01.2023	USA
GS_0096FW5.1	08.12.2022	USA
GS_009QLKK.1	25.01.2023	USA

GS_009VFWS.1	01.06.2023	United Kingdom
GS_009VFQ4.1	01.06.2023	United Kingdom
GS_009VLM5.1	01.06.2023	United Kingdom
GS_00A5CTF.1	17.11.2023	USA
GS_009WKRW.1	08.11.2023	USA
GS_00A5B9K.1	04.02.2024	USA
GS_009WUBG.1	05.11.2023	Japan
GS_009W58B.1	28.11.2023	United Kingdom
GS_009J581.1	16.07.2023	USA
GS_009ZHH8.1	01.11.2023	USA
GS_009ZKZ8.1	05.10.2023	USA
GS_00A4WCW.1	26.12.2023	USA
GS_00A51XJ.1	03.01.2024	USA
GS_009QNA2.1	03.09.2023	USA
GS_009ZECN.1	19.10.2023	USA
GS_009XN32.1	03.12.2023	USA
GS_009ZBUS.1	08.10.2023	USA
GS_00A45RU.1	19.12.2023	USA
GS_00A656V.1	11.12.2023	USA
GS_009Z7UW.1	27.09.2023	USA
GS_009X894.1	29.12.2023	USA
GS_00A3GDA.1	07.12.2023	USA
GS_00A043E.1	17.12.2023	USA
GS_009Z9AX.1	02.11.2023	USA
GS_00A4RS6.1	25.12.2023	USA
GS_009ZEUP.1	09.11.2023	USA
GS_009WKP0.1	09.11.2023	USA
GS_009XA76.1	26.12.2023	USA
GS_009X9ET.1	22.12.2023	USA
GS_009WHL8.1	21.11.2023	USA
GS_009ZPLW.1	29.12.2023	USA
GS_009ZK5X.1	14.11.2023	USA
GS_00A4XER.1	18.11.2023	USA
GS_00A4UZP.1	04.12.2023	USA
GS_009Z3T2.1	06.12.2023	USA
GS_00A5E2W.1	10.11.2023	USA
GS_00A5BMU.1	08.01.2024	USA

GS_00A5E5Q.1	06.12.2023	USA
GS_009J4M9.1	05.08.2023	USA
GS_009ZN8N.1	20.12.2023	USA
GS_00A58AL.1	30.11.2023	USA
GS_009ZBYJ.1	29.11.2023	USA
GS_00AKXR5.1	02.10.2023	USA
GS_009NP97.1	09.08.2023	USA
GS_00ABD2K.1	09.03.2024	USA
GS_009XQNV.1	11.12.2023	USA
GS_00A0SQH.1	23.01.2024	USA
GS_009ZBVQ.1	09.10.2023	USA
GS_00A9UK7.1	05.12.2023	USA
GS_009ZE44.1	24.10.2023	USA
GS_009ZE52.1	25.10.2023	USA
GS_00A25XL.1	12.02.2024	United Kingdom
GS_009Z8CU.1	01.12.2023	USA
GS_00A4ZSY.1	26.11.2023	USA
GS_009XUCC.1	23.12.2023	USA
GS_009WLST.1	27.11.2023	USA
GS_009Y8ZM.1	28.11.2023	USA
GS_009XT2Z.1	15.12.2023	USA
GS_00A310H.1	17.11.2023	Hong Kong
GS_00A4W4C.1	01.01.2024	USA
GS_00A4W3E.1	01.01.2024	USA
GS_009Z1U2.1	12.10.2023	USA
GS_00A597R.1	25.11.2023	USA
GS_00AFWLR.1	22.02.2024	USA
GS_009Z3ZQ.1	02.11.2023	USA
GS_009WU6S.1	08.11.2023	USA
GS_00A9UL5.1	03.12.2023	USA
GS_009Y6JK.1	06.12.2023	USA
GS_00A5E7L.1	06.12.2023	USA
GS_00A47TN.1	02.01.2024	USA
GS_009Z7MA.1	24.09.2023	USA
GS_009Z7YN.1	04.10.2023	USA
GS_009Y6EU.1	10.10.2023	Puerto Rico
GS_009Y6DW.1	11.10.2023	Puerto Rico
GS_009ZKK2.1	16.10.2023	USA

GS_009ZG18.1	21.10.2023	USA
GS_009ZGZB.1	30.10.2023	USA
GS_009ZAFL.1	01.11.2023	USA
GS_009ZBP2.1	02.12.2023	USA
GS_00A9WL3.1	12.12.2023	USA
GS_00A546Y.1	16.12.2023	USA
GS_009ZNAJ.1	18.12.2023	USA
GS_00ABEFS.1	01.01.2024	USA
GS_00A5F4R.1	03.01.2024	USA
GS_00A5EY3.1	18.12.2023	USA
GS_009Z6YP.1	21.09.2023	USA
GS_009Z288.1	29.10.2023	USA
GS_009WGN5.1	13.11.2023	USA
GS_009WLGE.1	24.11.2023	USA
GS_009W4QD.1	30.01.2024	Switzerland
GS_009X8VX.1	18.12.2023	USA
GS_00A4X83.1	23.12.2023	USA
GS_00A4WR3.1	14.12.2023	USA
GS_009YR11.1	25.12.2023	USA
GS_009ZG50.1	24.10.2023	USA
GS_009Z42J.1	08.11.2023	USA
GS_009ZLDE.1	04.12.2023	USA
GS_009WP6X.1	08.12.2023	USA
GS_00AM0BT.1	03.02.2024	USA
GS_00ABEA2.1	20.11.2023	USA
GS_009WULX.1	25.10.2023	Japan
GS_00A4TDX.1	14.11.2023	USA
GS_00A6341.1	05.02.2024	USA
GS_00AGT9F.1	12.12.2023	USA
GS_009ZG6Y.1	14.11.2023	USA
GS_009XN24.1	02.12.2023	USA
GS_00A0EVK.1	04.01.2024	USA
GS_009ZN12.1	15.12.2023	USA
GS_009YNK1.1	03.01.2024	United Kingdom
GS_00A3GH1.1	22.12.2023	USA
GS_0098FKP.1	10.02.2023	China
GS_009YNJ3.1	19.12.2023	United Kingdom
GS_00AWU9K.1	05.01.2025	USA

GS_009PTYN.1	02.05.2023	China
GS_009PTXQ.1	27.04.2023	China
GS_009W41S.1	29.01.2024	Switzerland
GS_00A4642.1	19.12.2023	USA
GS_009J5FM.1	18.07.2023	USA
GS_00A0Z5F.1	30.01.2024	Germany
GS_00A91CF.1	06.03.2024	Germany
GS_009WTEB.1	24.10.2023	USA
GS_009EGQ0.1	02.06.2023	Japan
GS_009YATX.1	16.11.2023	USA
GS_009YAJF.1	13.11.2023	USA
GS_009Y8UX.1	07.12.2023	USA
GS_00A4H8H.1	29.02.2024	USA
GS_009YPG7.1	03.01.2024	Japan
GS_00A1QQH.1	18.01.2024	USA
GS_00A1RE3.1	24.01.2024	USA
GS_00A2KUC.1	06.02.2024	USA
GS_00A916T.1	19.03.2024	South Korea
GS_00A3ENT.1	15.12.2023	USA
GS_009XBJG.1	13.12.2023	USA
GS_009XBYN.1	17.12.2023	USA
GS_009ZQ3W.1	31.12.2023	USA
GS_00A3FSJ.1	08.01.2024	USA
GS_00A0S0Y.1	15.01.2024	USA
GS_00ACVF9.1	13.05.2024	Germany
GS_00ABPSU.1	06.05.2024	Germany
GS_00ABPRW.1	06.05.2024	Germany
GS_00ABP9V.1	06.05.2024	Germany
GS_009YQ9L.1	15.12.2023	United Kingdom
GS_00A0XV2.1	28.12.2023	Germany
GS_009W4FY.1	25.01.2024	Switzerland
GS_009VR5Z.1	12.02.2024	United Kingdom
GS_00A3GRK.1	19.01.2024	USA
GS_009YQ12.1	14.12.2023	USA
GS_00A5T4D.1	01.03.2024	USA
GS_00A5QR7.1	04.03.2024	USA
GS_00A2D23.1	21.02.2024	USA
GS_00A331D.1	28.01.2024	USA

GS_00A0XRA.1	29.01.2024	Germany
GS_00A0XU4.1	31.01.2024	Germany
GS_00A2XU0.1	01.01.2024	Peru
GS_00A2XT2.1	01.01.2024	Peru
GS_00A27TS.1	29.12.2023	Peru
GS_00A27P0.1	28.12.2023	Peru
GS_00A27XJ.1	12.01.2024	Peru
GS_009X9WU.1	26.12.2023	USA
GS_00A2183.1	29.12.2023	USA
GS_00A5US1.1	05.02.2024	USA
GS_00A91MW.1	08.03.2024	USA
GS_00A9P57.1	15.02.2024	USA
GS_00A2G9L.1	15.02.2024	USA
GS_009Z53F.1	18.10.2023	USA
GS_00AM18Y.1	29.01.2024	USA
GS_00A0EJ7.1	01.01.2024	USA
GS_00A2MHY.1	26.02.2024	USA
GS_00AGY9A.1	01.04.2024	USA
GS_009YS10.1	27.12.2023	USA
GS_00AGT2V.1	01.04.2024	USA
GS_00AJ4M8.1	01.07.2024	Japan
GS_009VEHK.1	02.11.2023	United Kingdom
GS_00A9U1A.1	12.02.2024	USA
GS_00A0YNF.1	01.02.2024	United Kingdom
GS_00A0K21.1	08.01.2024	USA
GS_00A0XS8.1	30.01.2024	Germany
GS_009W45J.1	05.02.2024	Switzerland
GS_00A9RVQ.1	20.01.2024	USA
GS_00A5AUF.1	11.12.2023	USA
GS_00A61SS.1	15.02.2024	USA
GS_009W3D3.1	04.03.2024	Switzerland
GS_00A4HX4.1	21.02.2024	United Kingdom
GS_00AH24D.1	20.03.2024	USA
GS_009WHXL.1	29.11.2023	USA
GS_009WPPW.1	10.12.2023	USA
GS_009J4P5.1	18.07.2023	USA
GS_00A2XR6.1	01.01.2024	Peru
GS_00A5A4W.1	16.11.2023	USA

GS_009SR63.1	29.08.2023	USA
GS_009ZA0G.1	10.10.2023	USA
GS_009Z5ZN.1	23.10.2023	USA
GS_009WU04.1	01.11.2023	USA
GS_00A4TQ8.1	21.12.2023	USA
GS_00A60UP.1	02.01.2024	USA
GS_009Z5WU.1	16.10.2023	USA
GS_009Z63E.1	30.10.2023	USA
GS_00A4XAZ.1	10.11.2023	USA
GS_00A9U36.1	12.02.2024	USA
GS_009ZJ08.1	12.11.2023	USA
GS_00A2W0Q.1	13.02.2024	USA
GS_00A2XJL.1	01.01.2024	Peru
GS_00A2Y5C.1	01.01.2024	Peru
GS_00A2Y86.1	01.01.2024	Peru
GS_00AH3UX.1	04.03.2024	USA
GS_00A2PQG.1	02.03.2024	USA
GS_00A7T0H.1	13.03.2024	USA
GS_00AFWC8.1	28.12.2023	USA
GS_00A1WKM.1	19.01.2024	USA
GS_00A1RHW.1	25.01.2024	USA
GS_00AM055.1	04.02.2024	USA
GS_00A2J8L.1	13.02.2024	USA
GS_00A2WU1.1	22.02.2024	USA
GS_00A38EG.1	16.02.2024	USA
GS_00A5EAE.1	14.12.2023	USA
GS_00A9U9U.1	20.02.2024	USA
GS_00AH673.1	20.02.2024	USA
GS_00A1US9.1	12.02.2024	USA
GS_00ABGYP.1	15.04.2024	USA
GS_009Y6KH.1	07.12.2023	Germany
GS_00AHB8W.1	06.03.2024	USA
GS_00A55GB.1	18.12.2023	USA
GS_009YPXA.1	22.12.2023	USA
GS_009X80N.1	28.12.2023	USA
GS_00A0KXA.1	13.01.2024	USA
GS_00A0SBA.1	19.01.2024	USA
GS_00A0WRB.1	07.02.2024	USA
GS_00A2WDY.1	22.02.2024	USA

GS_00AH7Q1.1	02.03.2024	USA
GS_00AGZ2P.1	15.03.2024	USA
GS_00AF0HT.1	15.04.2024	USA
GS_00ABMRY.1	28.03.2024	USA
GS_00A03HL.1	29.11.2023	USA
GS_00A60PZ.1	12.12.2023	USA
GS_00A0GPV.1	16.01.2024	USA
GS_00A927Q.1	11.03.2024	USA
GS_00ALW1K.1	29.01.2024	USA
GS_00A6250.1	26.01.2024	USA
GS_00ALZUV.1	30.01.2024	USA
GS_00A4XUW.1	10.12.2023	USA
GS_00A2WR7.1	22.02.2024	USA
GS_009XNSP.1	11.12.2023	USA
GS_00A38K5.1	16.02.2024	USA
GS_009W3FZ.1	06.05.2024	Switzerland
GS_00AWSCF.1	02.01.2025	USA
GS_00A9UWK.1	07.12.2023	USA
GS_00AGSQK.1	14.03.2024	USA
GS_00AGUBA.1	10.04.2024	USA
GS_009XA3E.1	26.12.2023	USA
GS_00A5FNN.1	30.01.2024	USA
GS_00A5B7P.1	31.01.2024	USA
GS_00AGZ6F.1	04.03.2024	USA
GS_00A2M0Z.1	19.02.2024	USA
GS_009EGN4.1	20.06.2023	Japan
GS_00A0TV6.1	27.01.2024	USA
GS_009N2L5.1	22.08.2023	USA
GS_009W4S9.1	14.03.2024	Switzerland
GS_00ABNAU.1	25.03.2024	USA
GS_009Y6UZ.1	04.12.2023	Germany
GS_009ZNMV.1	28.12.2023	USA
GS_00A2UZT.1	02.02.2024	USA
GS_00AKX1L.1	18.02.2024	USA
GS_00A2464.1	27.02.2024	Germany
GS_00A26AU.1	27.02.2024	Germany
GS_00A26BS.1	27.02.2024	Germany
GS_00A26CQ.1	27.02.2024	Germany
GS_00A24ZH.1	27.02.2024	Germany

GS_00A26EL.1	27.02.2024	Germany
GS_00A24YK.1	27.02.2024	Germany
GS_00A26FJ.1	28.02.2024	Germany
GS_00A250F.1	28.02.2024	Germany
GS_00AETMU.1	08.12.2023	USA
GS_00ALYYN.1	29.07.2024	USA
GS_009ZJEE.1	09.10.2023	USA
GS_00AKTJP.1	27.03.2024	USA
GS_009ZF19.1	30.10.2023	USA
GS_009ZF0B.1	30.10.2023	USA
GS_00A5R2K.1	06.03.2024	USA
GS_009WNRT.1	28.11.2023	USA
GS_009YA74.1	30.10.2023	USA
GS_00A1UA8.1	08.01.2024	USA
GS_00A47QU.1	29.12.2023	USA
GS_009XTWA.1	19.12.2023	USA
GS_00A54CL.1	23.12.2023	USA
GS_00A9W26.1	04.02.2024	USA
GS_00A1TFZ.1	29.01.2024	USA
GS_00A2XKJ.1	01.01.2024	Peru
GS_00A9YFC.1	20.12.2023	USA
GS_00A3GF6.1	07.12.2023	USA
GS_009XSX9.1	14.12.2023	USA
GS_00A5BNS.1	14.01.2024	USA
GS_00A6614.1	01.02.2024	USA
GS_00A2FMW.1	11.02.2024	USA
GS_00A2KSG.1	05.02.2024	USA
GS_009WHWN.1	28.11.2023	USA
GS_00ALW77.1	28.01.2024	USA
GS_00AETJ0.1	16.03.2024	USA
GS_00AWU8M.1	05.01.2025	USA
GS_00AWWW8.1	03.01.2025	USA
GS_00AWUKY.1	06.01.2025	USA
GS_00A9XZA.1	11.02.2024	USA
GS_00A52N1.1	25.11.2023	USA
GS_009WPGB.1	09.12.2023	USA
GS_00AH6LA.1	26.02.2024	USA
GS_00A0THW.1	25.01.2024	USA
GS_009Z2R7.1	20.11.2023	USA

GS_00A62PX.1	16.01.2024	USA
GS_009WP8T.1	08.12.2023	USA
GS_009XB91.1	20.12.2023	USA
GS_00ALW69.1	07.02.2024	USA
GS_009ZA64.1	20.11.2023	USA
GS_00A5DD9.1	18.11.2023	USA
GS_00A9VSS.1	02.02.2024	USA
GS_00A4JF2.1	01.03.2024	USA
GS_00A7VUS.1	08.03.2024	USA
GS_00A4UVX.1	03.12.2023	USA
GS_009ZNKZ.1	16.12.2023	USA
GS_00A5D1Z.1	31.12.2023	USA
GS_009ZKL0.1	15.10.2023	USA
GS_009YABW.1	06.11.2023	USA
GS_00ABE5C.1	20.11.2023	USA
GS_00A4YM9.1	02.12.2023	USA
GS_009XU3W.1	21.12.2023	USA
GS_00A4725.1	23.12.2023	USA
GS_00A0CDL.1	08.01.2024	USA
GS_00A2PE3.1	01.03.2024	USA
GS_00A61CM.1	13.02.2024	USA
GS_00A61EH.1	29.01.2024	USA
GS_00AH459.1	12.03.2024	USA
GS_00A61GD.1	07.02.2024	USA
GS_00A2FQQ.1	11.02.2024	USA
GS_009X95B.1	19.12.2023	USA
GS_00A2CSP.1	19.02.2024	USA
GS_00A2T7D.1	25.02.2024	USA
GS_00A9QJD.1	24.02.2024	USA
GS_00A3A9Q.1	04.12.2023	USA
GS_00A3FE9.1	15.12.2023	USA
GS_00AF16F.1	22.05.2024	USA
GS_00A5UWT.1	14.02.2024	USA
GS_00A51BR.1	14.01.2024	USA
GS_00AGSW7.1	20.03.2024	USA
GS_00A2NV7.1	28.02.2024	USA
GS_00ABCR7.1	14.03.2024	USA
GS_00AH5P5.1	04.04.2024	USA
GS_00A3CLZ.1	27.01.2024	USA

GS_00AAEFU.1	01.02.2024	USA
GS_00A61M2.1	04.02.2024	USA
GS_00A9VL4.1	31.01.2024	USA
GS_00ALC05.1	22.10.2024	USA
GS_00A24P3.1	05.02.2024	Germany
GS_009X7JL.1	20.12.2023	USA
GS_009ZNCE.1	22.12.2023	USA
GS_00A1U6G.1	16.12.2023	USA
GS_00A66J3.1	09.02.2024	USA
GS_00A2547.1	13.02.2024	Italy
GS_00A26N3.1	13.02.2024	Italy
GS_00AWUAH.1	05.01.2025	USA
GS_00AGTLS.1	24.03.2024	USA
GS_00AKP5L.1	09.07.2024	USA
GS_00AUQXG.1	07.10.2024	USA
GS_00AKRQD.1	22.04.2024	USA
GS_00AEZGZ.1	05.05.2024	USA
GS_00A5BJ0.1	03.01.2024	USA
GS_00A5DY4.1	12.01.2024	USA
GS_00A64L1.1	26.01.2024	USA
GS_00AGVC7.1	11.04.2024	USA
GS_00ABMUS.1	22.04.2024	USA
GS_00AGEQX.1	15.07.2024	USA
GS_00AGXA9.1	24.04.2024	USA
GS_00ALY4B.1	30.08.2024	USA
GS_00AKUMG.1	25.06.2024	USA
GS_00AKRC4.1	02.07.2024	USA
GS_00AKTV1.1	05.07.2024	USA
GS_00AWS7R.1	02.01.2025	USA
GS_00AHCXG.1	31.05.2024	USA
GS_00AUR42.1	02.10.2024	USA
GS_00AUQJ8.1	22.10.2024	USA
GS_00ASLYN.1	16.11.2024	USA
GS_00AUE3F.1	29.10.2024	USA
GS_00AM1GG.1	26.08.2024	USA
GS_009W3X0.1	30.01.2024	Switzerland
GS_00A2WT3.1	20.02.2024	USA
GS_00A2SW1.1	16.02.2024	USA
GS_00A26HD.1	06.02.2024	Germany

GS_00A0KAK.1	08.01.2024	USA
GS_009W39B.1	31.01.2024	Switzerland
GS_00A0682.1	03.01.2024	USA
GS_00A063C.1	29.12.2023	USA
GS_009W2YZ.1	31.01.2024	Switzerland
GS_009W4YX.1	19.02.2024	Switzerland
GS_009YRG5.1	26.12.2023	USA
GS_00A269W.1	20.02.2024	Germany
GS_009W4C4.1	22.01.2024	Switzerland
GS_009W43N.1	23.01.2024	Switzerland
GS_00A2E06.1	06.01.2024	USA
GS_00AEQ9N.1	20.03.2024	USA
GS_00AGT1X.1	15.04.2024	USA
GS_00AWQ23.1	09.08.2024	USA
GS_00ALNJS.1	23.09.2024	USA
GS_00AMLRC.1	21.10.2024	Germany
GS_00AMLPG.1	21.10.2024	Germany
GS_00AR5PP.1	27.10.2024	USA
GS_00AWSAK.1	02.01.2025	USA
GS_00AUF1J.1	28.10.2024	USA
GS_00AWUE9.1	06.01.2025	USA
GS_00ASB9A.1	14.11.2024	USA
GS_009W4B6.1	11.03.2024	Switzerland
GS_00AWQ15.1	02.08.2024	USA
GS_00AETQN.1	02.05.2024	USA
GS_009W3KQ.1	02.02.2024	Switzerland
GS_00A4ZZJ.1	14.12.2023	USA
GS_009W32R.1	16.02.2024	Switzerland
GS_00AFXHW.1	16.07.2024	USA
GS_00AKQZW.1	09.07.2024	USA
GS_00ALYS0.1	18.08.2024	USA
GS_00A2670.1	06.02.2024	Germany
GS_00A2753.1	22.02.2024	United Kingdom
GS_00AL8VJ.1	30.09.2024	USA
GS_00AUEZN.1	04.11.2024	USA
GS_00AW4QC.1	17.12.2024	USA
GS_00AWUCD.1	05.01.2025	USA
GS_00AW8CZ.1	25.11.2024	USA

GS_00AVGGK.1	23.11.2024	USA
GS_00AURAQ.1	04.10.2024	USA
GS_00AUQWJ.1	05.10.2024	USA
GS_009W3ML.1	22.01.2024	Switzerland
GS_009W4E0.1	24.01.2024	Switzerland
GS_00ALYDT.1	26.02.2024	USA
GS_00AM0L8.1	23.07.2024	USA
GS_009YQBG.1	26.12.2023	United Kingdom
GS_009W4HT.1	15.01.2024	Switzerland
GS_009W4GW.1	15.01.2024	Switzerland
GS_009W3ZW.1	26.02.2024	Switzerland
GS_00AW5R9.1	16.12.2024	USA
GS_00AWRPT.1	29.12.2024	USA
GS_00AWSK0.1	04.01.2025	USA
GS_00AWSJ2.1	04.01.2025	USA
GS_00AWSDD.1	03.01.2025	USA
GS_00AWSF9.1	03.01.2025	USA
GS_00AWWUC.1	02.01.2025	USA
GS_00AWU7P.1	05.01.2025	USA
GS_00AWUPQ.1	08.01.2025	USA
GS_00AMLX0.1	21.10.2024	Japan
GS_00AKYDV.1	29.05.2024	Hong Kong
GS_00AL263.1	27.09.2024	Japan
GS_00AJL3V.1	28.08.2024	Japan
GS_00AJL7M.1	27.08.2024	Japan
GS_00AWSG7.1	03.01.2025	USA
GS_00AMLYY.1	09.10.2024	United Kingdom
GS_00AV897.1	05.11.2024	USA
GS_00AWRLZ.1	24.12.2024	USA
GS_00AWMH9.1	06.01.2025	USA
GS_00AWSBH.1	02.01.2025	USA
GS_00AWUG5.1	06.01.2025	USA
GS_00AR4Y6.1	13.11.2024	USA
GS_00AWW2X.1	20.11.2024	USA
GS_00AV988.1	03.12.2024	USA
GS_00AJV0S.1	24.08.2024	USA
GS_00AWUMU.1	07.01.2025	USA
GS_00AM1FJ.1	24.08.2024	USA

GS_00AR45T.1	24.10.2024	USA
GS_00AWUQN.1	08.01.2025	USA
GS_00AKTZT.1	02.07.2024	USA
GS_00AWUNS.1	07.01.2025	USA
GS_00AWWVA.1	02.01.2025	USA
GS_00AWW9H.1	20.12.2024	USA
GS_00ALP8C.1	22.10.2024	USA
GS_00AW3T7.1	26.11.2024	Slovakia
GS_00AUUQS.1	29.10.2024	USA
GS_00AUUPU.1	29.10.2024	USA
GS_00AWUBF.1	05.01.2025	USA
GS_00AWUF7.1	06.01.2025	USA
GS_00AWTNT.1	26.12.2024	USA
GS_00AWS6T.1	01.01.2025	USA
GS_00AWSEB.1	03.01.2025	USA
GS_00AWS9M.1	02.01.2025	USA
GS_00AUUCH.1	31.10.2024	USA
GS_00AWWTE.1	02.01.2025	USA
GS_00AWUJ0.1	06.01.2025	USA
GS_00AWNM0.1	22.12.2024	USA
GS_00AWS5V.1	01.01.2025	USA
GS_00AVC0M.1	11.12.2024	Germany
GS_00AVCDV.1	10.12.2024	Germany
GS_00A0DHA.1	21.12.2023	Italy
GS_00AWUDB.1	05.01.2025	USA
GS_00A7TR0.1	29.03.2024	USA
GS_009WJJB.1	25.10.2023	USA
GS_00AL22B.1	01.10.2024	USA
GS_009W49A.1	15.01.2024	Switzerland
GS_009W4W1.1	21.02.2024	Switzerland
GS_009W3C5.1	29.01.2024	Switzerland
GS_00A104E.1	06.02.2024	United Kingdom
GS_009YQDC.1	03.01.2024	United Kingdom
GS_00A2XME.1	01.01.2024	Peru
GS_00A2662.1	06.02.2024	Germany
GS_009W3V4.1	02.02.2024	Switzerland
GS_00A27UQ.1	04.01.2024	Peru
GS_009W40U.1	16.01.2024	Switzerland

GS_009W4D2.1	15.01.2024	Switzerland
GS_00A0YS7.1	01.02.2024	United Kingdom
GS_009W3A9.1	06.02.2024	Switzerland
GS_009VR33.1	06.02.2024	United Kingdom
GS_009VR41.1	04.02.2024	United Kingdom
GS_009VR25.1	06.02.2024	United Kingdom
GS_009W4V3.1	26.01.2024	Switzerland
GS_009W3GX.1	14.02.2024	Switzerland
GS_00A0Z6D.1	24.01.2024	Germany
GS_00A26K9.1	08.02.2024	Germany
GS_009W3E1.1	02.04.2024	Switzerland
GS_009XNPV.1	10.12.2023	USA
GS_009W47E.1	13.02.2024	Switzerland
GS_009W31T.1	19.01.2024	Switzerland
GS_009W2X1.1	29.01.2024	Switzerland
GS_00AERK0.1	23.01.2024	USA
GS_00AWQNW.1	06.12.2024	USA
GS_00A0WXZ.1	08.02.2024	USA
GS_00A0U1U.1	28.01.2024	USA
GS_00AJ4LA.1	01.07.2024	Germany
GS_009W34M.1	01.03.2024	Switzerland
GS_00ABH3D.1	01.04.2024	USA
GS_00AKQX0.1	20.05.2024	USA
GS_009WHP2.1	21.11.2023	USA
GS_00AWUH2.1	06.01.2025	USA
GS_009YQCE.1	28.12.2023	United Kingdom
GS_009W4RB.1	30.01.2024	Switzerland
GS_009W4MK.1	05.02.2024	Switzerland
GS_00A24AW.1	06.02.2024	United Kingdom
GS_00A249Y.1	06.02.2024	United Kingdom
GS_009W3W2.1	23.02.2024	Switzerland
GS_00A252B.1	21.02.2024	Germany

Table S6: Accession codes for GenBank, of the global genomic sequences used for the RSV G gene phylogenetic tree

accession	date	country
MZ417571	21.09.2018	Taiwan
PQ599899	24.01.2024	Mexico
PQ599900	30.01.2024	Mexico
PP376265	01.04.2019	South Africa
PP376453	27.12.2018	France
PP376578	11.12.2018	Spain
PP376633	16.12.2019	Spain
PP376549	09.12.2017	Finland
PP376459	28.01.2018	Finland
PP376546	08.04.2019	Finland
PP376786	30.04.2019	Finland
PP376349	17.08.2018	Brazil
PP376278	10.07.2019	Brazil
PP376680	02.06.2019	Australia
PP376434	25.08.2019	Australia
PP376682	02.09.2019	Japan
PP376605	13.02.2020	South Korea
PP376504	04.12.2019	South Korea
PP376530	06.12.2019	South Korea
PP376356	18.12.2019	South Korea
PP376548	09.01.2020	South Korea
KJ723474	08.01.1989	USA
KJ723486	31.12.1979	USA
DQ171792	01.01.1998	New Zealand
DQ171800	31.12.1987	New Zealand
DQ171797	01.01.1988	New Zealand
MG642079	30.12.1982	USA
HQ731719	31.12.1985	United Kingdom
MG642083	31.12.1987	USA
DQ171807	01.01.1994	New Zealand
KU316133	17.01.1990	USA
KU316176	05.04.1990	USA
JX198115	10.01.1991	USA
KP258728	31.12.1992	USA
JX513382	31.08.2001	Brazil

KJ627725	01.01.2004	USA
JX069803	04.03.2000	USA
JX661572	10.02.2000	Germany
PQ591393	28.09.2008	United Kingdom
JX015485	04.01.2005	Netherlands
JX015488	09.01.2006	Netherlands
DQ171829	10.11.1997	New Zealand
JX513310	15.01.2002	Brazil
KP317949	13.02.2003	Kenya
KT765815	02.04.2004	Kenya
JQ901449	10.01.2001	Netherlands
KF826827	26.05.2004	Argentina
HQ731698	01.01.2006	Thailand
KJ627662	31.12.2003	USA
HQ731773	01.01.2007	United Kingdom
HQ731774	01.01.2007	United Kingdom
KF826828	04.06.2004	Argentina
JX513298	31.12.2005	Brazil
JX513309	31.12.2005	Brazil
KF826826	24.12.2004	Mexico
KF826841	02.05.2007	Argentina
KF826850	01.01.2008	USA
KU950561	05.01.2006	USA
KF826836	04.01.2006	Mexico
MG642077	17.07.1987	USA
DQ171786	30.09.1986	New Zealand
HQ731716	16.10.1981	United Kingdom
DQ171761	01.01.1989	New Zealand
DQ171743	14.08.1982	New Zealand
HQ731720	31.12.1984	United Kingdom
DQ171742	01.01.1988	New Zealand
OR795296	20.03.2000	Germany
KU316166	31.12.1977	USA
KT765780	18.01.2000	Kenya
OK649657	01.01.1995	Canada
MG642050	01.01.1994	USA
KU316118	01.01.1996	USA
KC476743	01.01.2009	South Africa
OR795314	06.01.2005	Germany

KF826848	01.01.2007	Australia
JQ901452	22.12.2001	Netherlands
JQ901447	03.01.2001	Netherlands
JX661580	02.10.2004	Germany
JX015486	05.01.2005	Netherlands
OR795322	05.02.2007	Germany
HQ731775	29.06.2007	United Kingdom
KT765661	18.02.2000	Kenya
KT765664	21.07.2000	Kenya
KT765663	13.03.2000	Kenya
MT854048	24.01.2013	Zambia
JX131638	16.03.2008	Saudi Arabia
JX182822	25.05.2007	Brazil
JX182849	09.04.2010	Brazil
KF826838	06.06.2006	Argentina
JF920047	17.01.2008	USA
JX513320	31.12.2007	Brazil
JX015481	07.01.2009	Netherlands
JX645814	10.12.2009	Belgium
JX645812	11.12.2009	Belgium
KU955051	08.04.2008	China
KF300971	14.10.2008	Panama
HQ731725	01.01.2010	United Kingdom
JX645796	14.12.2008	Belgium
JX015482	22.11.2006	Belgium
JF714705	25.02.2008	Saudi Arabia
JX661578	10.04.2008	Germany
KF156409	09.12.2008	Kenya
KU955056	31.12.2007	China
KC342394	22.07.2010	Thailand
HQ731730	01.01.2010	United Kingdom
KM508818	04.07.2010	Paraguay
KM508820	07.07.2011	Paraguay
HQ731735	31.12.2009	United Kingdom
KC297421	02.01.2009	China
JF979153	11.03.2010	Latvia
KR816586	11.12.2009	Thailand
MK344683	21.09.2009	Taiwan
KY327967	01.09.2012	Thailand

MK344715	31.12.2012	Taiwan
KT765285	25.04.2011	Kenya
KT765427	02.01.2011	Kenya
KX510263	13.04.2010	Kenya
KJ939949	26.03.2010	Vietnam
JX976353	05.11.2011	Cuba
MK109775	14.09.2011	Jordan
KY327980	01.09.2012	Thailand
JX661579	19.04.2008	Germany
JX645831	13.12.2010	Belgium
KC763335	03.10.2010	Saudi Arabia
JX645830	18.01.2011	Belgium
KC476658	15.04.2009	South Africa
MN262219	14.03.2011	Mozambique
KC476668	01.01.2010	South Africa
KU955113	01.01.2007	China
MN604236	01.03.2011	Netherlands
JX645825	31.10.2008	Belgium
KU955165	03.06.2007	China
KU955083	01.01.2007	China
KU955170	01.01.2011	China
JX131639	16.03.2008	Saudi Arabia
FJ157348	31.07.2005	China
MK182714	01.03.2012	Saudi Arabia
OR795330	24.04.2010	Germany
MH760600	22.09.2011	Australia
MH760593	17.06.2010	Australia
JX513301	01.01.2011	Brazil
KF915183	05.12.2011	Spain
MG971423	29.01.2013	Netherlands
MT853822	10.09.2013	Mali
MT853882	05.07.2012	South Africa
MN262215	16.12.2010	Morocco
OR795335	08.02.2012	Germany
MT853793	28.11.2012	Mali
OR795333	10.01.2011	Germany
JN257701	28.01.2011	Canada
OQ261746	29.01.2019	Austria
MK720784	01.01.2018	Colombia

MH181939	07.11.2012	Kenya
ON237288	24.06.2016	Argentina
MT853770	01.03.2013	Gambia
MZ961918	31.01.2016	China
KX453332	13.03.2013	Kenya
KU350791	12.06.2012	Argentina
KF915251	19.12.2012	Spain
OR795341	18.01.2013	Germany
KC677857	15.10.2012	Cuba
KC677871	19.09.2012	Cuba
MH142239	30.12.2016	Russia
OR795347	18.03.2014	Germany
KU350778	09.05.2012	Argentina
MH760607	08.07.2013	Australia
KY865207	01.01.2014	Egypt
KU350811	13.06.2013	Argentina
JN257694	19.01.2011	Canada
JN257693	29.12.2010	Canada
PP974146	08.01.2024	China
MN031363	23.12.2016	Cambodia
OP927977	13.03.2018	Germany
OR288011	13.03.2019	Zambia
MH750252	01.01.2015	Brazil
KT781345	24.03.2014	China
MG971424	14.02.2013	Netherlands
OR162242	08.03.2022	Kenya
OR795354	25.02.2016	Germany
MF496477	01.01.2015	Taiwan
MH388030	04.01.2016	Saudi Arabia
PP529983	02.03.2020	Hungary
MW678361	22.07.2019	Thailand
KX765972	01.07.2013	New Zealand
KX765919	09.06.2013	New Zealand
KX765954	08.05.2013	New Zealand
MF445799	21.11.2011	China
KU681161	07.10.2013	South Korea
MH828493	07.11.2013	Vietnam
MG971433	03.10.2016	Netherlands
MW160778	22.03.2017	Australia

MN434119	01.01.2017	Saudi Arabia
MH828491	10.12.2013	Vietnam
MH142232	25.03.2014	Russia
MH142234	18.03.2013	Russia
MF496378	25.10.2013	Taiwan
PP525308	01.01.2017	USA
MZ961932	30.11.2017	China
OY757645	28.11.2022	Australia
OP554443	01.05.2022	Saudi Arabia
PP376653	18.12.2018	Canada
PP958027	31.12.2017	Ireland
MN007064	02.02.2018	China
KY328023	08.10.2014	Thailand
MK306343	01.01.2016	Taiwan
KU681168	08.12.2014	South Korea
PP352304	28.02.2018	USA
MG971426	24.12.2014	Netherlands
PP957980	01.01.2016	Ireland
MN630100	01.02.2016	USA
PP454564	01.01.2023	Saudi Arabia
PP454566	01.01.2023	Saudi Arabia
MH760610	19.11.2016	Australia
MK306338	31.12.2016	Taiwan
MW160761	31.03.2017	Australia
MG793382	01.02.2015	Lebanon
PP109421	30.10.2017	United Kingdom
PQ349000	09.05.2022	United Kingdom
PP969952	21.09.2022	Ireland
OR287922	04.01.2020	USA
OR522506	22.01.2023	USA
PQ788245	17.08.2024	USA
OR522487	28.11.2022	USA
OR872596	14.10.2022	USA
PP969998	15.11.2023	Ireland
PP969999	20.11.2023	Ireland
PP454567	31.12.2023	Saudi Arabia
PQ066224	15.02.2024	USA
PP270247	09.10.2023	USA
OR522464	01.11.2022	USA

OP890337	30.11.2022	USA
OR522510	15.01.2023	USA
PQ762763	30.12.2023	France
PP203259	31.12.2022	USA
PQ638697	08.11.2022	USA
OR522502	15.12.2022	USA
OR522523	08.12.2022	USA
PQ638681	25.12.2022	USA
PP203263	31.12.2022	USA
OP890316	01.10.2022	USA
PQ638726	13.10.2022	USA
PQ638680	15.12.2022	USA
PQ638709	07.10.2022	USA
PP770463	15.01.2024	USA
PQ390738	31.12.2023	USA
PQ390744	26.12.2023	USA
PP342420	01.12.2023	USA
PQ638788	15.11.2023	USA
PQ638791	11.11.2023	USA
PQ638792	03.11.2023	USA
PQ638719	27.10.2023	USA
PQ638714	17.10.2023	USA
PQ638716	11.10.2023	USA
PQ410479	31.12.2018	Belgium
OQ171919	06.08.2022	USA
PP681273	03.01.2023	USA
OR601479	12.02.2023	USA
OR975300	05.02.2023	USA
OQ024137	06.11.2022	USA
OQ024114	04.11.2022	USA
OQ024116	03.11.2022	USA
OQ171922	23.10.2022	USA
OQ171920	17.10.2022	USA
OQ024149	05.11.2022	USA
PP352376	31.12.2022	USA
OR975303	30.11.2022	USA
OR915732	03.11.2022	USA
OQ171916	23.10.2022	USA
OQ171894	20.07.2022	USA

PP970055	31.10.2022	Ireland
PP352342	30.11.2022	USA
OQ171909	11.08.2022	USA
PP352377	30.11.2022	USA
OR601475	23.10.2022	USA
OQ024120	03.11.2022	USA
OQ171896	11.08.2022	USA
OQ024139	04.11.2022	USA
OQ171899	23.10.2022	USA
MW026979	01.03.2018	Brazil
OP927929	27.02.2020	Germany
ON237343	29.06.2017	Argentina
MT236290	29.06.2017	Brazil
PP529974	28.12.2018	Hungary
MZ515750	21.12.2019	Spain
OR872589	31.10.2022	USA
PQ834838	28.10.2022	Mexico
PQ834839	09.11.2022	Mexico
OR522503	18.12.2022	USA
OR522501	18.12.2022	USA
OR522518	04.12.2022	USA
OR601477	19.12.2022	USA
OR601476	17.12.2022	USA
OR883002	25.05.2023	USA
OR522521	11.12.2022	USA
PP084056	10.12.2022	USA
OR522499	18.12.2022	USA
OR522504	26.12.2022	USA
PP352326	01.10.2022	USA
PP681267	13.12.2022	USA
PP352375	30.11.2022	USA
PP352350	31.10.2022	USA
PP352356	01.10.2022	USA
PQ618030	24.10.2022	Panama
PQ618020	19.12.2022	Panama
PQ618026	22.09.2022	Panama
PQ618021	08.11.2022	Panama
PQ618027	11.08.2022	Panama
PQ618025	03.10.2022	Panama

PQ618022	08.11.2022	Panama
PQ618023	27.10.2022	Panama
PQ618029	10.10.2022	Panama
PQ618028	10.10.2022	Panama
PQ618024	20.10.2022	Panama
PQ618032	20.10.2022	Panama
PQ618031	01.01.2023	Panama
PQ834849	22.12.2022	Mexico
OR143142	12.12.2022	USA
PQ834848	13.12.2022	Mexico
OQ171901	18.10.2022	USA
OR872628	21.09.2022	USA
OR522491	06.12.2022	USA
OR872640	09.11.2022	USA
OR872622	27.04.2022	USA
PQ638693	11.11.2022	USA
PP352333	10.10.2022	USA
PP352327	10.10.2022	USA
OQ024109	08.11.2022	USA
PP529981	17.01.2023	Hungary
OR883007	27.11.2022	USA
OQ024136	06.11.2022	USA
PQ762819	09.11.2023	France
PQ763132	27.12.2023	France
PP352325	01.10.2022	USA
PP352357	31.10.2022	USA
PP681275	05.01.2023	USA
OQ024121	05.11.2022	USA
PP959043	04.01.2024	USA
OR143222	30.11.2022	USA
OR872602	26.10.2022	USA
OR872625	15.10.2022	USA
OR872632	18.08.2022	USA
OR522507	23.01.2023	USA
PQ638706	16.11.2022	USA
OR601474	23.10.2022	USA
PQ638704	16.11.2022	USA
OR601472	16.10.2022	USA
PQ638674	23.11.2022	USA

PQ638692	11.01.2023	USA
PQ638730	15.10.2022	USA
OR975305	22.12.2022	USA
OR601473	16.10.2022	USA
OR522462	30.10.2022	USA
PQ638695	08.11.2022	USA
OR522479	23.10.2022	USA
PQ638668	25.11.2022	USA
OR522520	30.01.2023	USA
PQ638675	30.11.2022	USA
PQ638688	11.11.2022	USA
OR522512	10.01.2023	USA
PP342434	17.12.2023	USA
OR143189	29.01.2023	USA
PP352351	01.10.2022	USA
PP352368	01.11.2022	USA
OR143184	20.02.2023	USA
PP352352	01.10.2022	USA
PP681243	06.12.2022	USA
PP352354	31.10.2022	USA
OR872644	24.10.2022	USA
OR143154	09.12.2022	USA
OR143168	02.12.2022	USA
PP352341	01.11.2022	USA
OR522517	05.12.2022	USA
OR522498	18.10.2022	USA
OR522497	18.10.2022	USA
PP495848	09.01.2024	USA
PP973778	16.02.2024	USA
PP495851	15.01.2024	USA
PP978499	16.02.2024	USA
PQ849780	05.12.2024	USA
PQ849777	06.12.2024	USA
PQ849795	03.12.2024	USA
PQ834845	25.10.2022	Mexico
PQ834844	26.10.2022	Mexico
PQ788250	14.01.2024	USA
PP781407	27.01.2024	USA
PQ834878	07.12.2022	Mexico

PQ834875	18.11.2022	Mexico
PQ834869	16.11.2022	Mexico
PQ834847	07.11.2022	Mexico
OR872587	22.07.2022	USA
OR143170	19.12.2022	USA
OR143179	02.01.2023	USA
OR872585	17.10.2022	USA
PQ638673	25.11.2022	USA
PP352369	01.11.2022	USA
PQ066219	21.12.2023	USA
PP352347	01.10.2022	USA
PP594806	24.11.2023	USA
PP973769	05.02.2024	USA
PP910757	16.01.2024	USA
PP910760	14.01.2024	USA
PP973748	10.01.2024	USA
PP973750	08.01.2024	USA
PP709397	02.01.2024	USA
PP709386	27.12.2023	USA
PP709375	19.12.2023	USA
PP709374	18.12.2023	USA
PP973754	22.01.2024	USA
PP910759	15.01.2024	USA
PP973749	09.01.2024	USA
PP934460	07.03.2024	USA
PP973760	29.01.2024	USA
PP973753	22.01.2024	USA
PP973752	21.01.2024	USA
PP973758	29.01.2024	USA
PP973757	29.01.2024	USA
PP352345	30.11.2022	USA
OR143223	31.12.2022	USA
OR143224	04.12.2022	USA
PQ638703	22.03.2023	USA
PP795161	06.01.2024	USA
PP882667	21.11.2023	USA
PP270250	13.11.2023	USA
PP882659	17.10.2023	USA
PP970062	27.10.2023	Ireland

PP970036	04.11.2023	Ireland
PP969961	30.10.2023	Ireland
PP970003	24.11.2023	Ireland
PP970063	06.11.2023	Ireland
PP970029	29.10.2023	Ireland
PP970025	18.10.2023	Ireland
PP970023	13.10.2023	Ireland
PQ763136	17.01.2024	France
PQ763005	29.12.2023	France
PQ763126	24.11.2023	France
OR883014	14.12.2022	USA
PP781387	17.01.2024	USA
PP934468	30.03.2024	USA
PP934470	01.04.2024	USA
PP957778	03.04.2024	USA
PQ849789	28.11.2024	USA
PP830408	21.12.2023	USA
PP957771	28.03.2024	USA
PP978527	03.03.2024	USA
PQ763164	12.03.2024	France
PQ762815	30.12.2023	France
PQ762814	27.12.2023	France
PQ763060	21.12.2023	France
PQ763094	12.12.2023	France
PQ763092	05.12.2023	France
PQ763085	25.11.2023	France
PQ763083	21.11.2023	France
PQ763091	02.12.2023	France
PQ763108	05.12.2023	France
PQ762920	03.11.2023	France
PQ763101	29.12.2023	France
PQ763096	15.12.2023	France
PQ763163	06.02.2024	France
PQ763088	27.11.2023	France
PQ763087	27.11.2023	France
PQ763089	01.12.2023	France
PQ763093	06.12.2023	France
PQ763098	21.12.2023	France
PQ763086	26.11.2023	France

PQ762810	27.11.2023	France
PQ763084	23.11.2023	France
PQ763097	18.12.2023	France
PQ763090	01.12.2023	France
PQ762843	08.01.2024	France
PQ762986	29.12.2023	France
PQ763011	27.11.2023	France
PQ763010	26.12.2023	France
PQ762981	24.12.2023	France
PQ763000	22.12.2023	France
PQ762996	11.02.2024	France
PQ763001	25.11.2023	France
PQ763059	20.12.2023	France
PQ762653	19.12.2023	France
PQ763012	28.11.2023	France
PQ762997	14.12.2023	France
PQ763006	20.12.2023	France
PQ763017	17.12.2023	France
PQ762706	17.12.2023	France
PQ763003	05.01.2024	France
PQ762982	13.12.2023	France
PP781409	23.01.2024	USA
PP847361	09.01.2024	USA
PP781397	29.01.2024	USA
PP278015	23.10.2023	USA
PQ066216	01.01.2024	USA
PQ066222	19.12.2023	USA
PP795137	19.10.2023	USA
PP847379	11.01.2024	USA
PP668165	03.12.2023	USA
PP386340	27.10.2023	USA
PP135017	07.09.2023	USA
PP770476	20.02.2024	USA
PQ788206	25.11.2024	USA
PQ788208	22.11.2024	USA
PP847386	21.01.2024	USA
PP709445	28.12.2023	USA
PP386335	26.10.2023	USA
PP504644	13.11.2023	USA

PP847370	04.01.2024	USA
PP830389	16.12.2023	USA
PP830395	14.12.2023	USA
PP957776	06.04.2024	USA
PP957772	30.03.2024	USA
PQ762507	19.03.2024	USA
PQ762505	18.03.2024	USA
PP978523	26.02.2024	USA
PP910783	12.02.2024	USA
PP910776	29.01.2024	USA
PP847387	18.01.2024	USA
PP830410	23.12.2023	USA
PP270262	04.12.2023	USA
PP795149	15.11.2023	USA
PP795142	28.10.2023	USA
PP795140	26.10.2023	USA
PP795136	16.10.2023	USA
PP795160	02.01.2024	USA
PQ762522	02.05.2024	USA
PP957765	21.03.2024	USA
PP978535	11.03.2024	USA
PP978526	02.03.2024	USA
PQ762517	28.04.2024	USA
PP795145	01.11.2023	USA
PP934452	02.03.2024	USA
PP934456	05.03.2024	USA
PP934472	02.04.2024	USA
PP934469	01.04.2024	USA
PP830398	17.12.2023	USA
PP978529	05.03.2024	USA
PP847393	18.01.2024	USA
PP594796	17.11.2023	USA
PP973747	12.01.2024	USA
PP871355	03.10.2023	USA
PP957760	07.02.2024	USA
PQ762508	19.03.2024	USA
PP847369	09.01.2024	USA
PP957767	25.03.2024	USA
PP934473	13.04.2024	USA

PP934474	13.04.2024	USA
PP978498	16.02.2024	USA
PP978525	02.03.2024	USA
PP910771	27.01.2024	USA
OQ261751	06.05.2022	Austria
PP781389	14.02.2024	USA
PP969942	28.12.2022	Ireland
OR162259	13.04.2022	Kenya
OR162265	19.05.2022	Kenya
OR162267	24.05.2022	Kenya
OR162247	10.03.2022	Kenya
OR162238	02.03.2022	Kenya
OR162234	01.03.2022	Kenya
OR162253	19.03.2022	Kenya
PQ763129	11.12.2023	France
PQ763128	05.12.2023	France
OR287842	29.10.2019	USA
PQ762803	11.12.2023	France
PQ117646	04.06.2024	USA
PQ763074	06.01.2024	France
OR162266	22.05.2022	Kenya
PP830378	04.12.2023	USA
OR162255	25.03.2022	Kenya
OR162261	19.04.2022	Kenya
OR162256	28.03.2022	Kenya
OR162260	13.04.2022	Kenya
OR162268	26.05.2022	Kenya
OR162271	22.06.2022	Kenya
OR162257	30.03.2022	Kenya
OR162250	12.03.2022	Kenya
OR162235	02.03.2022	Kenya
OR162258	10.04.2022	Kenya
OR162249	11.03.2022	Kenya
OR143206	07.12.2022	USA
PQ349026	13.04.2023	United Kingdom
PQ763133	02.01.2024	France
PQ762764	03.01.2024	France
PQ763106	02.12.2023	France
PQ762656	24.12.2023	France

PP970021	02.10.2023	Ireland
PQ762659	12.01.2024	France
PQ762851	05.12.2023	France
PQ762725	21.11.2023	France
PP970004	24.11.2023	Ireland
PQ763161	12.12.2023	France
PQ762752	15.11.2023	France
OM857287	29.11.2020	Australia
PP530274	08.12.2023	USA
PQ762995	20.01.2024	France
PQ763077	26.12.2023	France
PQ763075	28.12.2023	France
PQ763141	19.01.2024	France
PQ762701	07.12.2023	France
PQ762993	12.11.2023	France
PQ763049	16.12.2023	France
PQ410468	13.11.2022	Belgium
PQ376587	25.10.2023	Belgium
OR872610	07.10.2022	USA
PQ763052	24.11.2023	France
PQ763144	11.12.2023	France
PQ762719	13.12.2023	France
PQ763032	04.02.2024	France
PQ762731	06.12.2023	France
PQ762671	28.11.2023	France
PQ763051	26.11.2023	France
PQ762722	30.10.2023	France
PQ762745	18.10.2023	France
PQ763110	08.12.2023	France
PQ763115	14.12.2023	France
PQ763113	11.12.2023	France
PQ763118	26.12.2023	France
PQ763151	10.12.2023	France
PQ762902	02.01.2024	France
PQ763042	12.12.2023	France
PQ763165	12.12.2023	France
PQ762771	31.12.2023	France
PQ762712	24.12.2023	France
PQ762947	10.12.2023	France

PQ762944	08.12.2023	France
PQ762931	04.12.2023	France
PQ763080	04.12.2023	France
PQ762922	18.11.2023	France
PQ762928	28.11.2023	France
PQ762932	06.12.2023	France
PQ762924	21.11.2023	France
PQ763160	30.11.2023	France
PQ762943	07.12.2023	France
PQ763008	26.12.2023	France
PQ763099	25.12.2023	France
PQ762934	11.12.2023	France
PQ762919	01.11.2023	France
PQ762936	28.11.2023	France
PQ762933	09.12.2023	France
PQ762939	04.12.2023	France
PQ762941	05.12.2023	France
PQ762935	12.12.2023	France
PQ762923	19.11.2023	France
PQ763111	19.12.2023	France
PQ763079	21.12.2023	France
PQ762809	09.12.2023	France
PQ762794	28.11.2023	France
PQ763078	13.11.2023	France
PQ762795	08.11.2023	France
PQ762693	21.11.2023	France
PQ762930	02.12.2023	France
PQ762925	24.11.2023	France
PQ762921	08.11.2023	France
PQ762899	09.01.2024	France
PQ763138	04.12.2023	France
PQ762696	05.12.2023	France
PQ762945	10.12.2023	France
PQ762950	03.12.2023	France
PQ762948	11.12.2023	France
PQ762927	26.11.2023	France
PQ763114	13.12.2023	France
PQ763015	26.03.2024	France
PQ763058	20.12.2023	France

PQ762988	25.12.2023	France
PQ762709	19.12.2023	France
PQ762697	06.12.2023	France
PQ763162	11.01.2024	France
OR162251	14.03.2022	Kenya
OR162246	10.03.2022	Kenya
OR162252	16.03.2022	Kenya
OR162237	01.03.2022	Kenya
OR162269	04.06.2022	Kenya
OR162278	07.09.2022	Kenya
OR162233	28.02.2022	Kenya
OR162272	23.06.2022	Kenya
OR162232	27.02.2022	Kenya
OR162245	09.03.2022	Kenya
PP970034	04.11.2023	Ireland
PP969949	18.07.2022	Ireland
PP969941	03.12.2022	Ireland
PP969982	08.12.2022	Ireland
PP970013	30.11.2022	Ireland
PP969995	13.12.2022	Ireland
PP970014	19.12.2022	Ireland
PP352361	30.11.2022	USA
PP352362	01.11.2022	USA
PP352330	01.10.2022	USA
PP352340	01.11.2022	USA
PP970020	04.11.2023	Ireland
PP978522	26.02.2024	USA
PQ788199	26.11.2024	USA
PP969988	25.11.2023	Ireland
PQ788216	19.11.2024	USA
PP770469	27.02.2024	USA
PQ066220	21.12.2023	USA
PQ348860	29.07.2022	United Kingdom
PP969997	14.11.2023	Ireland
PQ349023	06.02.2023	United Kingdom
PQ348886	05.11.2022	United Kingdom
PQ763120	04.01.2024	France
PQ763117	24.12.2023	France
PQ763107	04.12.2023	France

PQ763135	05.01.2024	France
PP781416	14.12.2023	USA
PP970017	01.11.2023	Ireland
PP970042	19.11.2023	Ireland
PQ762807	03.12.2023	France
PQ762624	08.11.2023	France
PQ737347	08.11.2023	France
PQ762834	24.11.2023	France
PQ762870	23.12.2023	France
PP781415	17.02.2024	USA
PQ762979	21.12.2023	France
PQ763140	18.01.2024	France
PQ762787	18.12.2023	France
PQ762946	10.12.2023	France
PQ763076	04.01.2024	France
PQ763041	03.12.2023	France
PQ763007	31.03.2024	France
PQ763149	24.12.2023	France
PQ763014	22.12.2023	France
PQ763130	18.12.2023	France
PQ762910	18.12.2023	France
PQ763150	15.12.2023	France
PQ763153	12.12.2023	France
PQ763152	10.12.2023	France
PQ763127	02.12.2023	France
PQ762994	28.11.2023	France
PQ762980	22.11.2023	France
PQ762820	09.11.2023	France
PQ762816	08.11.2023	France
PQ762655	24.12.2023	France
PQ762866	24.11.2023	France
PQ762718	12.12.2023	France
PQ763123	23.12.2023	France
PQ762742	11.12.2023	France
PQ762677	31.10.2023	France
PQ762644	08.12.2023	France
PQ737367	08.12.2023	France
PQ762914	23.11.2023	France
PQ763156	13.11.2023	France

PQ762664	28.11.2023	France
PQ762758	13.12.2023	France
PQ763116	18.12.2023	France
PQ762858	16.12.2023	France
PQ763057	05.12.2023	France
PQ762913	17.11.2023	France
PQ763050	17.11.2023	France
PQ763104	23.10.2023	France
PQ763054	23.12.2023	France
PQ762859	16.12.2023	France
PQ762918	23.11.2023	France
PQ762833	12.12.2023	France
PQ763105	05.12.2023	France
PQ762825	02.11.2023	France
PQ762873	19.12.2023	France
PQ763124	31.12.2023	France
PQ763157	21.11.2023	France
PQ763037	03.12.2023	France
PQ762668	17.12.2023	France
PQ763145	25.12.2023	France
PQ849798	28.11.2024	USA
PQ762883	08.01.2024	France
PQ763102	04.01.2024	France
PP959045	04.01.2024	USA
PQ762658	03.01.2024	France
PQ763002	27.12.2023	France
PQ762911	27.12.2023	France
PQ763016	27.12.2023	France
PQ762813	25.12.2023	France
PQ762942	06.12.2023	France
PQ737366	06.12.2023	France
PQ762872	26.11.2023	France
PQ762620	02.11.2023	France
PQ737353	30.10.2023	France
PQ762892	25.10.2023	France
PQ376586	19.10.2023	Belgium
PQ763056	17.12.2023	France
PQ737343	02.11.2023	France
PQ763061	12.01.2024	France

PQ762663	26.12.2023	France
PQ763109	14.12.2023	France
PQ762828	17.12.2023	France
PQ763095	12.12.2023	France
PQ762630	30.10.2023	France
PQ762643	06.12.2023	France
PQ763009	27.12.2023	France
PQ762735	06.12.2023	France
PQ762740	30.11.2023	France
PQ762733	27.11.2023	France
PQ762730	26.11.2023	France
PQ763069	02.01.2024	France
PQ763070	13.12.2023	France
PQ737345	29.10.2023	France
PQ762622	29.10.2023	France
PQ618042	01.12.2022	Panama
PP270276	30.11.2023	USA
PP270275	04.12.2023	USA
PP495846	03.01.2024	USA
PP957756	07.02.2024	USA
OR143138	19.12.2022	USA
OR143220	16.12.2022	USA
OR882975	17.11.2022	USA
OR872645	25.10.2022	USA
OR872626	26.10.2022	USA
OR143197	30.11.2022	USA
OR882985	07.11.2022	USA
OR882981	04.11.2022	USA
OR915734	03.11.2022	USA
OR915756	24.10.2022	USA
PP903812	22.10.2022	USA
OR882974	26.11.2022	USA
OR882978	29.11.2022	USA
PP970035	04.11.2023	Ireland
PP970032	31.10.2023	Ireland
MZ515640	31.12.2019	Spain
PQ618033	15.01.2020	Panama
OR915735	19.01.2023	USA
OQ024155	04.11.2022	USA

PP681283	05.12.2022	USA
PP681242	06.12.2022	USA
OR143137	13.12.2022	USA
OR522496	17.10.2022	USA
OR143188	25.12.2022	USA
PP760419	15.10.2022	USA
OP890314	15.10.2022	USA
PP352344	30.11.2022	USA
OY757638	22.07.2022	Australia
OY757684	22.07.2022	Australia
OY757641	15.07.2022	Australia
OY757595	15.07.2022	Australia
PP770472	19.02.2024	USA
OR143209	19.12.2022	USA
PP969963	04.12.2023	Ireland
PP969991	29.11.2023	Ireland
PP969989	26.11.2023	Ireland
PP970018	02.11.2023	Ireland
PP970019	01.11.2023	Ireland
PP970026	20.10.2023	Ireland
PP970022	11.10.2023	Ireland
PP970033	26.10.2023	Ireland
PP969957	24.11.2023	Ireland
PP970028	28.10.2023	Ireland
PP970044	15.11.2023	Ireland
PP970037	06.11.2023	Ireland
PP970061	19.10.2023	Ireland
PP970046	19.11.2023	Ireland
PP969962	04.12.2023	Ireland
PP970041	19.11.2023	Ireland
PP969985	16.11.2023	Ireland
PP969984	16.11.2023	Ireland
PP970005	17.11.2023	Ireland
PP969958	23.11.2023	Ireland
PP970000	17.11.2023	Ireland
PP969986	27.11.2023	Ireland
PP969959	15.11.2023	Ireland
PP969956	22.11.2023	Ireland
PQ849787	03.12.2024	USA

PQ762801	20.11.2023	France
PQ349019	27.09.2022	United Kingdom
PQ348960	27.09.2022	United Kingdom
PQ348963	27.09.2022	United Kingdom
PQ348962	27.09.2022	United Kingdom
PQ763064	20.12.2023	France
PQ737349	05.12.2023	France
PQ762626	05.12.2023	France
PQ762769	19.12.2023	France
PQ763112	22.12.2023	France
PQ762726	24.11.2023	France
PQ763071	06.01.2024	France
PQ762721	31.10.2023	France
PQ762749	30.10.2023	France
OR522473	06.11.2022	USA
OR872616	28.09.2022	USA
PP910758	16.01.2024	USA
PP352338	01.10.2022	USA
PQ618041	20.09.2022	Panama
PQ618040	31.10.2022	Panama
OQ171931	01.07.2022	USA
OQ171911	13.07.2022	USA
OQ024154	14.11.2022	USA
OQ171926	26.10.2022	USA
OR143183	01.12.2022	USA
OR143198	06.12.2022	USA
OQ171897	23.10.2022	USA
OQ024152	03.11.2022	USA
PQ762661	15.01.2024	France
PQ762634	13.11.2023	France
PQ737357	13.11.2023	France
PP681280	10.01.2023	USA
PP203260	12.10.2022	USA
PP681260	13.12.2022	USA
PQ638672	24.11.2022	USA
PP681270	20.12.2022	USA
OR522526	30.01.2023	USA
OR522527	01.02.2023	USA
OQ024124	14.11.2022	USA

OQ024148	03.11.2022	USA
PQ618039	23.12.2023	Panama
OR601470	02.10.2022	USA
OR872619	21.06.2022	USA
PP903821	21.10.2022	USA
PP903827	21.10.2022	USA
PP970008	17.11.2022	Ireland
PP970054	30.10.2022	Ireland
PP970052	19.10.2022	Ireland
PP970051	17.10.2022	Ireland
PQ348882	16.10.2022	United Kingdom
PQ348881	16.10.2022	United Kingdom
PQ348877	08.10.2022	United Kingdom
PQ348884	19.10.2022	United Kingdom
PQ348883	17.10.2022	United Kingdom
PQ348875	03.10.2022	United Kingdom
PQ348862	27.08.2022	United Kingdom
PQ349022	21.11.2022	United Kingdom
PQ348869	09.09.2022	United Kingdom
PQ348861	28.08.2022	United Kingdom
PQ348880	13.10.2022	United Kingdom
PQ348868	09.09.2022	United Kingdom
PQ348865	28.08.2022	United Kingdom
PQ348876	04.10.2022	United Kingdom
PQ348864	14.08.2022	United Kingdom
PQ348859	26.07.2022	United Kingdom
PP970058	08.12.2022	Ireland
PQ349008	19.07.2022	United Kingdom
PQ349014	08.08.2022	United Kingdom
PQ349013	04.08.2022	United Kingdom
PQ349012	01.08.2022	United Kingdom
PQ349009	22.07.2022	United Kingdom
PQ349006	13.07.2022	United Kingdom
PQ348967	18.03.2022	United Kingdom
PQ348965	18.03.2022	United Kingdom
PQ348999	18.03.2022	United Kingdom
PQ349015	09.08.2022	United Kingdom
PQ348879	12.10.2022	United Kingdom
PQ348961	11.07.2022	United Kingdom

PQ349004	11.07.2022	United Kingdom
PP970056	03.11.2022	Ireland
PP970049	11.10.2022	Ireland
PP970048	11.10.2022	Ireland
PQ349007	16.07.2022	United Kingdom
PQ349018	18.09.2022	United Kingdom
PQ349001	17.05.2022	United Kingdom
PP969950	10.09.2022	Ireland
PQ349005	13.07.2022	United Kingdom
PQ349017	05.09.2022	United Kingdom
PQ349010	26.07.2022	United Kingdom
PP781403	02.02.2024	USA
PP270232	24.10.2023	USA
PP270274	06.12.2023	USA
PP781402	28.12.2023	USA
PP781388	05.12.2023	USA
PP903809	16.03.2024	USA
PP270251	23.10.2023	USA
PP781418	05.02.2024	USA
PP270271	28.11.2023	USA
OR882986	16.05.2023	USA
PP270253	02.10.2023	USA
PP781417	14.12.2023	USA
PP781420	18.12.2023	USA
PP270272	12.12.2023	USA
PP270231	21.11.2023	USA
PP781383	15.01.2024	USA
PP270270	02.12.2023	USA
PP795159	03.01.2024	USA
PP882666	14.11.2023	USA
PP386365	05.11.2023	USA
PP795133	05.10.2023	USA
PP882665	08.11.2023	USA
PQ762761	15.12.2023	France
PQ762897	02.11.2023	France
PQ762882	03.12.2023	France
OQ171906	27.07.2022	USA
OR522529	01.01.2023	USA
OR143187	26.12.2022	USA

PP530267	29.11.2023	USA
PQ610207	11.06.2023	Argentina
PQ390732	04.01.2024	USA
PP882664	02.11.2023	USA
PQ788217	20.11.2024	USA
PQ849796	28.11.2024	USA
PQ788215	13.11.2024	USA
OR143171	01.02.2023	USA
OR143166	01.02.2023	USA
PQ610205	15.06.2023	Argentina
PQ610204	04.06.2023	Argentina
PQ610203	23.05.2023	Argentina
PQ762938	02.12.2023	France
PQ610209	15.05.2023	Argentina
PP934463	17.03.2024	USA
PQ762759	14.12.2023	France
PQ763158	24.11.2023	France
PQ610198	22.05.2023	Argentina
PQ610200	29.05.2023	Argentina
PP934471	02.04.2024	USA
PP957750	03.02.2024	USA
PQ762509	19.03.2024	USA
PQ762523	04.05.2024	USA
PQ610199	27.05.2023	Argentina
PQ610197	12.05.2023	Argentina
PQ610196	25.05.2023	Argentina
PQ610202	11.05.2023	Argentina
PQ610201	17.05.2023	Argentina
PP790963	04.12.2023	Mexico
PQ610208	23.06.2023	Argentina
PQ610206	22.05.2023	Argentina
PQ834887	21.11.2023	Mexico
PP495853	31.01.2024	USA
PP770471	11.01.2024	USA
PQ834895	04.12.2023	Mexico
PQ834890	27.11.2023	Mexico
PQ834883	14.11.2023	Mexico
PQ834879	16.10.2023	Mexico
PQ834901	13.12.2023	Mexico

PQ834899	06.12.2023	Mexico
PQ762876	28.11.2023	France
PQ762875	28.11.2023	France
PP969992	29.11.2023	Ireland
PP978501	21.02.2024	USA
PP978536	11.03.2024	USA
PP978534	10.03.2024	USA
PP795148	10.11.2023	USA
PQ618038	13.10.2023	Panama
PQ610195	03.07.2024	Argentina
PQ117649	02.01.2024	USA
PP668172	05.12.2023	USA
PP709446	29.12.2023	USA
PP781425	02.01.2024	USA
PP342431	13.12.2023	USA
PQ834857	28.11.2022	Mexico
PQ834872	18.11.2022	Mexico
PQ638684	31.12.2022	USA
PQ638670	22.11.2022	USA
PQ638676	12.11.2022	USA
PP781390	29.02.2024	USA
OR872588	23.10.2022	USA
OR872637	16.10.2022	USA
OR872607	25.09.2022	USA
OR975306	29.09.2023	USA
PQ638682	23.12.2022	USA
PP795156	14.12.2023	USA
PP795162	19.01.2024	USA
PP795157	22.12.2023	USA
PQ834855	04.11.2022	Mexico
PQ834858	28.11.2022	Mexico
PQ834870	16.11.2022	Mexico
PQ834861	11.01.2023	Mexico
PQ834907	16.01.2024	Mexico
PQ834908	24.01.2024	Mexico
PP342419	30.11.2023	USA
PP342426	05.12.2023	USA
PQ008881	11.12.2023	USA
OR872618	13.09.2022	USA

OR883018	07.11.2022	USA
OR882991	01.11.2022	USA
OR915770	15.11.2022	USA
PP781426	23.02.2024	USA
PP270243	01.11.2023	USA
PP270265	06.12.2023	USA
PP781378	03.01.2024	USA
PP781406	22.12.2023	USA
PP781385	30.01.2024	USA
PQ834866	27.02.2023	Mexico
PQ834860	02.12.2022	Mexico
PQ834868	15.11.2022	Mexico
PP084057	25.10.2023	USA
PP352384	01.12.2022	USA
PQ834862	16.01.2023	Mexico
PQ638696	11.11.2022	USA
PQ763004	04.10.2023	France
PQ762987	28.11.2023	France
PQ763013	26.12.2023	France
PQ762896	05.12.2023	France
PQ762901	04.12.2023	France
PQ762887	12.12.2023	France
PQ762908	01.12.2023	France
PQ834897	05.12.2023	Mexico
PQ834900	08.12.2023	Mexico
PQ834905	14.12.2023	Mexico
PQ638722	29.01.2023	USA
PQ834876	01.12.2022	Mexico
PQ834854	01.11.2022	Mexico
OR975308	24.10.2023	USA
PP342441	04.01.2024	USA
PP084058	02.11.2023	USA
PP970024	13.10.2023	Ireland
PP342416	19.11.2023	USA
PP970040	20.11.2023	Ireland
OR975307	22.10.2023	USA
PP342435	17.12.2023	USA
PQ008877	01.01.2024	USA
PP342438	20.12.2023	USA

PP342422	04.12.2023	USA
PQ834863	24.01.2023	Mexico
PQ834864	24.01.2023	Mexico
PP852062	13.02.2024	USA
PP910768	26.01.2024	USA
PP847391	17.01.2024	USA
PP978537	12.03.2024	USA
PQ762526	19.06.2024	USA
OR872583	01.10.2022	USA
PQ834865	15.02.2023	Mexico
PQ834874	18.11.2022	Mexico
PQ834871	16.11.2022	Mexico
PP352349	01.10.2022	USA
PQ834867	14.11.2022	Mexico
PP978496	31.12.2024	USA
PP342439	20.12.2023	USA
PP969987	27.11.2023	Ireland
PQ849801	20.09.2024	USA
PQ762916	31.12.2023	France
PQ638678	01.12.2022	USA
PQ762799	15.12.2023	France
PQ762798	15.12.2023	France
PQ763155	12.11.2023	France
MW678370	18.09.2019	Thailand
MW678310	29.10.2018	Thailand
PP151402	16.09.2022	Kuwait
PP151344	19.10.2021	Kuwait
PP151368	11.05.2022	Kuwait
PP957764	22.03.2024	USA
PP709396	04.01.2024	USA
OR840708	15.04.2023	China
OR840704	05.04.2023	China
PP974155	10.12.2023	China
PP270249	03.11.2023	USA
PP903814	19.03.2024	USA
PQ638797	08.11.2023	USA
PP760389	01.10.2022	USA
OR883017	13.04.2023	USA
OQ171910	14.10.2022	USA

PP508181	30.05.2022	Peru
OR522477	10.11.2022	USA
OR872621	16.09.2022	USA
PP352346	31.10.2022	USA
PP135031	22.09.2023	USA
PP342432	14.12.2023	USA
OR143140	19.12.2022	USA
PQ834856	16.11.2022	Mexico
PP781413	31.01.2024	USA
PQ618051	07.12.2022	Panama
PQ390730	29.12.2023	USA
PQ762990	29.11.2023	France
PP135024	18.09.2023	USA
PP352386	31.12.2022	USA
PQ618052	17.10.2022	Panama
PQ638662	22.11.2022	USA
OR522493	02.10.2022	USA
OR522494	27.09.2022	USA
OR143195	13.12.2022	USA
PP270255	25.10.2023	USA
PQ618048	21.06.2024	Panama
PQ618049	21.06.2024	Panama
PQ618046	28.06.2024	Panama
PQ618045	11.07.2024	Panama
PQ618047	11.07.2024	Panama
PP795134	11.10.2023	USA
PP084059	04.11.2023	USA
PQ618050	11.07.2024	Panama
PP084060	08.11.2023	USA
PP795147	07.11.2023	USA
PQ117653	12.01.2024	USA
PP969969	18.10.2022	Ireland
PQ638666	22.11.2022	USA
PQ638699	11.11.2022	USA
OR522481	06.12.2022	USA
PP530268	28.11.2023	USA
PP270234	01.11.2023	USA
PP969943	04.10.2022	Ireland
PP969951	22.09.2022	Ireland

PP454563	16.02.2023	Saudi Arabia
OR162274	14.07.2022	Kenya
PQ762676	08.10.2023	France
PQ762736	04.12.2023	France
OR522470	06.11.2022	USA
OR975298	06.11.2022	USA
PP970007	12.11.2022	Ireland
PP352367	30.11.2022	USA
PQ348870	11.09.2022	United Kingdom
PQ348871	19.09.2022	United Kingdom
OR872581	26.09.2022	USA
PQ192488	05.10.2023	Thailand
PQ348889	14.12.2022	United Kingdom
PQ348874	28.09.2022	United Kingdom
PQ348890	20.12.2022	United Kingdom
PQ849774	07.12.2024	USA
OR872604	01.08.2022	USA
OP927864	19.01.2022	Germany
OR143218	17.12.2022	USA
PQ762495	13.03.2024	USA
PQ788236	08.11.2024	USA
PQ788244	24.10.2024	USA
PQ348888	28.12.2022	United Kingdom
PP529967	11.02.2023	Hungary
PP970012	13.12.2022	Ireland
PP969953	26.09.2022	Ireland
PP970045	17.11.2023	Ireland
PQ788229	13.11.2024	USA
PQ788196	22.11.2024	USA
PQ788220	19.11.2024	USA
PQ788205	23.11.2024	USA
PQ788237	05.11.2024	USA
PP454562	31.12.2023	Saudi Arabia
PP454570	13.10.2023	Saudi Arabia
PP760393	31.10.2022	USA
PQ763055	03.12.2023	France
PP681261	14.12.2022	USA
PP270266	05.12.2023	USA
PP270277	03.12.2023	USA

PP847388	18.01.2024	USA
PP978538	12.03.2024	USA
PP957743	01.02.2024	USA
PP978517	24.02.2024	USA
PQ849781	09.12.2024	USA
PQ008880	21.12.2023	USA
OR143144	08.12.2022	USA
PP681262	14.12.2022	USA
PP495852	18.01.2024	USA
PQ762871	17.12.2023	France
PQ762741	03.12.2023	France
PQ762868	02.12.2023	France
PQ763137	29.11.2023	France
PQ762991	26.11.2023	France
PQ410476	01.01.2018	Belgium
OQ248602	30.11.2022	China
OR140550	01.05.2023	China
OR140549	02.04.2023	China
OR140554	01.01.2023	China
OQ248606	01.12.2022	China
OQ248607	31.12.2022	China
OQ024158	05.11.2022	USA
PP974164	10.12.2023	China
PP974167	18.11.2022	China
PQ626971	12.08.2024	China
PP833560	01.03.2024	China
PQ762685	26.09.2023	France
OR840707	12.04.2023	China
PP974163	18.12.2023	China
PP974168	19.12.2023	China
PP974169	03.01.2024	China
OR840709	21.04.2023	China
OR840706	11.04.2023	China
PP970030	31.10.2023	Ireland
PP974161	08.12.2023	China
PP819400	14.01.2024	China
PQ192487	28.11.2023	Thailand
PP974162	22.12.2023	China
PQ762684	24.09.2023	France

OR872636	11.10.2022	USA
PP411979	13.08.2023	Thailand
ON152648	18.10.2021	Russia
OR143177	06.12.2022	USA
PQ618058	11.08.2023	Panama
PQ618059	27.02.2023	Panama
OQ024138	14.11.2022	USA
OQ024143	14.11.2022	USA
OR143207	30.11.2022	USA
PQ618057	12.06.2024	Panama
OQ024157	14.11.2022	USA
OQ171900	20.07.2022	USA
OQ024135	05.11.2022	USA
PP970011	29.11.2022	Ireland
OQ024122	03.11.2022	USA
MZ961930	31.10.2017	China
OQ248601	01.11.2022	China
OR140547	01.04.2023	China
OR140552	31.05.2023	China
PP974156	18.12.2023	China
OR140544	01.02.2023	China
OR140543	01.02.2023	China
PP974159	18.11.2022	China
OR140546	01.04.2023	China
OR140545	01.04.2023	China
OR140551	01.05.2023	China
PP668167	03.12.2023	USA
PP847377	05.01.2024	USA
LC816568	10.08.2023	Japan
PQ066217	18.12.2023	USA
PP781382	06.01.2024	USA
PP781414	04.01.2024	USA
PQ762886	06.12.2023	France
OR795475	07.11.2022	Germany
OR795481	05.12.2022	Germany
PP386343	27.10.2023	USA
PP709453	01.01.2024	USA
PQ762491	28.01.2024	USA
PP910764	25.01.2024	USA

PP847395	21.01.2024	USA
PQ788247	12.01.2024	USA
PP709398	01.01.2024	USA
PP594813	22.11.2023	USA
PP504628	05.11.2023	USA
PP386349	28.10.2023	USA
PP386344	27.10.2023	USA
PP386337	25.10.2023	USA
PP135023	12.09.2023	USA
PP847380	11.01.2024	USA
PP978507	17.02.2024	USA
PQ762940	04.12.2023	France
PP352334	01.10.2022	USA
PP352363	30.11.2022	USA
PP681264	12.12.2022	USA
PP342424	05.12.2023	USA
PP342425	06.12.2023	USA
PP795151	22.11.2023	USA
PQ618056	23.05.2022	Panama
PQ618055	31.10.2022	Panama
OR882972	08.05.2023	USA
PQ348895	17.10.2022	United Kingdom
PQ348893	13.10.2022	United Kingdom
PQ348892	10.10.2022	United Kingdom
PP709379	22.12.2023	USA
PP770466	14.02.2024	USA
PP770461	19.01.2024	USA
PQ638796	02.11.2023	USA
PP084061	15.11.2023	USA
PQ008879	10.11.2023	USA
PP342430	09.12.2023	USA
PP770452	31.01.2024	USA
PP934458	11.03.2024	USA
PP342423	05.12.2023	USA
PQ638790	11.11.2023	USA
PQ638799	02.11.2023	USA
PQ638718	19.10.2023	USA
PQ638715	11.10.2023	USA
OR915772	13.08.2023	USA

PP903815	12.04.2024	USA
PQ390740	25.11.2023	USA
PQ638720	29.10.2023	USA
PP770459	22.01.2024	USA
PQ638784	12.12.2023	USA
PP776593	29.02.2024	USA
PP959040	20.02.2024	USA
PP959047	10.01.2024	USA
PP530264	15.12.2023	USA
PP709370	05.12.2023	USA
PQ390742	25.11.2023	USA
PP709395	03.01.2024	USA
PP709392	04.01.2024	USA
PP709369	04.12.2023	USA
PP530269	03.01.2024	USA
PP847358	22.12.2023	USA
PP830387	16.12.2023	USA
PP401819	28.11.2023	USA
PP709385	26.12.2023	USA
LC846999	02.09.2019	Myanmar
LC847002	12.09.2020	Myanmar
LC847027	06.12.2021	Myanmar
OQ024119	03.11.2022	USA
OQ024123	03.11.2022	USA
OR287942	13.01.2020	USA
OR666541	01.12.2019	China
LC847003	22.08.2020	Myanmar
OQ933826	25.11.2021	China
PQ117650	11.03.2024	USA
PP974160	11.01.2024	China
LC816567	26.10.2022	Japan
OR795370	31.12.2018	Germany
OR140542	31.01.2023	China
OQ248604	01.12.2022	China
OQ248603	01.12.2022	China
PP352337	30.11.2022	USA
OQ024111	08.11.2022	USA
PP352378	01.11.2022	USA
PQ834853	19.10.2022	Mexico

OR522460	26.10.2022	USA
OR522461	26.10.2022	USA
PQ638724	12.10.2022	USA
PP760397	01.11.2022	USA
PP760422	01.11.2022	USA
OP890319	01.11.2022	USA
OR522474	08.11.2022	USA
OR975302	30.11.2022	USA
OR522516	04.12.2022	USA
OR975301	27.11.2022	USA
OR522524	01.01.2023	USA
PP401815	24.11.2023	USA
PP352385	01.12.2022	USA
PP681272	05.01.2023	USA
PP352382	01.11.2022	USA
OQ024141	14.11.2022	USA
LC847044	23.07.2022	Myanmar
LC847045	28.07.2022	Myanmar
OR162280	28.01.2023	Kenya
OR872605	30.10.2022	USA
OR162279	28.01.2023	Kenya
OP890315	01.10.2022	USA
OR795478	22.11.2022	Germany
PP760406	31.01.2023	USA
PP910780	13.02.2024	USA
PP847359	06.01.2024	USA
PP504654	13.11.2023	USA
PP978521	25.02.2024	USA
PQ762520	08.05.2024	USA
PQ762483	13.01.2024	USA
PP386360	05.11.2023	USA
PP957763	10.02.2024	USA
PP973780	10.02.2024	USA
OP690363	31.12.2020	Taiwan
OR795480	07.12.2022	Germany
OR162275	19.09.2022	Kenya
PP970047	20.11.2023	Ireland
PQ762961	12.12.2023	France
PP957761	08.02.2024	USA

PP957749	01.02.2024	USA
PP910767	26.01.2024	USA
PQ762487	15.01.2024	USA
PP847363	09.01.2024	USA
PP668171	05.12.2023	USA
PP594816	04.12.2023	USA
PP504647	13.11.2023	USA
PP237786	09.10.2023	USA
PP237784	09.10.2023	USA
PP709447	30.12.2023	USA
PP847382	13.01.2024	USA
PP668163	03.12.2023	USA
PP401817	27.11.2023	USA
PP594814	26.11.2023	USA
PQ788252	15.01.2024	USA
PP910752	26.12.2023	USA
PP847378	11.01.2024	USA
PQ762679	21.01.2024	France
PP974158	10.12.2023	China
PP411981	13.07.2023	Thailand
PP411984	12.08.2023	Thailand
PQ638721	01.08.2023	USA
OQ248605	01.12.2022	China
OR140553	01.05.2023	China
PP974165	11.01.2024	China
PP974166	20.12.2023	China
PQ763068	05.12.2023	France
PQ763039	12.11.2023	France
PQ762756	05.12.2023	France
PQ763143	08.12.2023	France
OQ024128	03.11.2022	USA
OR872641	10.10.2022	USA
OR872608	22.06.2022	USA
OR872623	14.07.2022	USA
OQ024115	02.11.2022	USA
PP795131	03.10.2023	USA
PQ834859	30.11.2022	Mexico
PP830388	17.12.2023	USA
PP270242	14.11.2023	USA

PP352379	31.12.2022	USA
PP781419	03.01.2024	USA
PP504630	02.11.2023	USA
PP401816	23.11.2023	USA
PP709450	30.12.2023	USA
PP957780	13.04.2024	USA
PQ762512	20.04.2024	USA
PP681277	10.01.2023	USA
OQ261753	02.11.2022	Austria
OP890340	31.10.2022	USA
OR143213	17.12.2022	USA
OR143139	14.12.2022	USA
OR143212	08.12.2022	USA
OR143204	04.12.2022	USA
PP681274	03.01.2023	USA
PQ834906	02.01.2024	Mexico
PQ834902	13.12.2023	Mexico
PQ834880	08.11.2023	Mexico
PQ834881	13.11.2023	Mexico
PQ834888	22.11.2023	Mexico
PQ834904	13.12.2023	Mexico
PQ618043	22.07.2024	Panama
PQ618044	05.07.2024	Panama
PQ008876	13.12.2023	USA
PP342429	08.12.2023	USA
PP342433	17.12.2023	USA
PP342440	26.12.2023	USA
PP342436	19.12.2023	USA
PQ008871	29.12.2023	USA
PQ008872	29.12.2023	USA
OY757830	15.09.2022	Australia
PP454569	04.06.2023	Saudi Arabia
PP969976	06.11.2022	Ireland
PP969974	02.11.2022	Ireland
PQ066221	17.04.2024	USA
PQ788231	08.11.2024	USA
PQ849778	06.12.2024	USA
PQ849773	07.12.2024	USA
PQ849797	01.12.2024	USA

PQ788227	14.11.2024	USA
PQ788200	25.11.2024	USA
PQ788212	04.11.2024	USA
PQ849782	09.12.2024	USA
PQ788232	09.11.2024	USA
PQ849771	09.12.2024	USA
PQ788235	12.11.2024	USA
PQ788210	22.11.2024	USA
PQ788211	22.11.2024	USA
PQ788209	24.11.2024	USA
PQ788224	19.11.2024	USA
PQ788238	05.11.2024	USA
PQ849779	05.12.2024	USA
PQ849799	29.11.2024	USA
PQ788234	12.11.2024	USA
PQ788228	14.11.2024	USA
PQ849775	06.12.2024	USA
PQ849785	08.12.2024	USA
PQ788241	31.10.2024	USA
PQ849794	28.11.2024	USA
PQ849792	29.11.2024	USA
PQ849776	06.12.2024	USA
PQ788197	24.11.2024	USA
PQ788233	09.11.2024	USA
PQ849788	01.12.2024	USA
PQ788239	03.11.2024	USA
PQ849800	27.11.2024	USA
PQ788226	16.11.2024	USA
PQ788222	19.11.2024	USA
PQ788198	27.11.2024	USA
PQ849793	03.12.2024	USA
PQ849786	08.12.2024	USA
PQ788207	26.11.2024	USA
PQ788221	19.11.2024	USA
OR162270	12.06.2022	Kenya
OR162262	26.04.2022	Kenya
OR162254	21.03.2022	Kenya
OR162276	19.03.2022	Kenya
PP530261	03.01.2024	USA

PP709373	18.12.2023	USA
PP795165	11.02.2024	USA
PQ762511	20.03.2024	USA
PQ762498	13.03.2024	USA
OR143202	06.01.2023	USA
OR522468	06.11.2022	USA
OR882992	28.02.2023	USA
OR882988	26.02.2023	USA
PP594804	22.11.2023	USA
PQ638725	12.10.2022	USA
PQ638723	12.10.2022	USA
PQ638717	03.11.2022	USA
OR502561	30.09.2021	India
PP969973	01.11.2022	Ireland
PP970010	13.11.2022	Ireland
PP969978	13.11.2022	Ireland
PP969975	06.11.2022	Ireland
PP969967	13.10.2022	Ireland
PP352353	31.10.2022	USA
PP352371	01.12.2022	USA
PQ788243	27.10.2024	USA
PP270237	14.11.2023	USA
PQ348968	10.09.2022	United Kingdom
PP781405	08.01.2024	USA
PP781384	03.01.2024	USA
PP270256	14.11.2023	USA
PP270239	25.10.2023	USA
OQ171918	19.07.2022	USA
OQ024125	05.11.2022	USA
OQ024150	05.11.2022	USA
PP978530	05.03.2024	USA
PQ788204	23.11.2024	USA
PQ788214	11.11.2024	USA
PQ788218	20.11.2024	USA
PQ788202	04.10.2024	USA
PQ788201	26.11.2024	USA
PQ788219	20.11.2024	USA
PP135009	08.08.2023	USA
OR143210	01.01.2023	USA

PP352355	01.10.2022	USA
PP681251	08.12.2022	USA
PP352381	01.12.2022	USA
OR872627	28.10.2022	USA
PP270241	10.10.2023	USA
OP890322	01.10.2022	USA
PP681281	23.11.2022	USA
PP352364	30.11.2022	USA
PP934451	03.03.2024	USA
PP973771	05.02.2024	USA
PP934461	12.03.2024	USA
PP852061	11.02.2024	USA
PP973779	09.02.2024	USA
OR872600	12.10.2022	USA
PP781410	22.12.2023	USA
PP270257	04.12.2023	USA
OR915737	31.10.2022	USA
PP903805	31.10.2022	USA
OR883010	28.10.2022	USA
OR882969	18.05.2023	USA
PP903830	28.10.2022	USA
OR915760	24.10.2022	USA
PP969948	28.06.2022	Ireland
OQ261748	09.09.2021	Austria
PP386359	03.11.2023	USA
PP970059	13.11.2022	Ireland
PQ762768	15.12.2023	France
PQ762850	05.12.2023	France
PQ763159	26.11.2023	France
PQ762690	26.09.2023	France
PQ762724	23.11.2023	France
PQ763139	05.12.2023	France
PQ762915	01.12.2023	France
PQ762817	08.11.2023	France
PQ762864	22.12.2023	France
PQ762845	07.11.2023	France
PQ762848	04.12.2023	France
PQ762983	07.01.2024	France
PQ762860	18.12.2023	France

PQ763122	13.12.2023	France
PQ762852	10.12.2023	France
PQ762846	01.12.2023	France
PQ762865	20.12.2023	France
PQ762862	30.12.2023	France
PQ762861	21.12.2023	France
PQ762844	06.11.2023	France
PQ762975	12.12.2023	France
PQ763073	12.12.2023	France
PQ762732	27.11.2023	France
PQ762779	30.11.2023	France
PQ762974	11.12.2023	France
PQ762956	05.11.2023	France
PQ762951	10.01.2024	France
PQ762953	15.10.2023	France
PQ763147	29.12.2023	France
PQ762898	03.11.2023	France
PQ762905	19.12.2023	France
PQ763031	09.02.2024	France
PQ762784	12.12.2023	France
PQ763168	28.11.2023	France
PQ762890	18.12.2023	France
PQ763067	03.12.2023	France
PQ762906	29.12.2023	France
PQ762774	28.12.2023	France
PQ762651	16.12.2023	France
PQ762879	02.11.2023	France
PQ763034	25.01.2024	France
PQ762881	04.12.2023	France
PQ763146	27.12.2023	France
PQ762646	07.12.2023	France
PQ762785	06.01.2024	France
PQ762877	28.11.2023	France
PQ762964	09.12.2023	France
PQ762965	28.01.2024	France
PQ762999	25.11.2023	France
PQ762824	02.11.2023	France
PQ762826	17.12.2023	France
PQ762667	02.12.2023	France

PQ762757	07.12.2023	France
PQ762904	08.12.2023	France
PQ762699	07.12.2023	France
PQ762836	27.11.2023	France
PQ762751	10.11.2023	France
PQ762838	04.12.2023	France
PQ117652	09.01.2024	USA
PQ117648	15.12.2023	USA
PQ762707	17.12.2023	France
PQ762762	19.12.2023	France
PQ762728	24.11.2023	France
PQ762710	20.12.2023	France
PQ762680	03.12.2023	France
PQ762884	31.12.2023	France
PQ762926	25.11.2023	France
PQ762797	15.12.2023	France
PQ763045	23.11.2023	France
PQ762970	21.11.2023	France
PQ762992	28.11.2023	France
KX453505	29.04.2015	Kenya
OR795343	30.12.2013	Germany
KX765977	12.02.2014	New Zealand
MH142228	20.03.2014	Russia
PP660602	16.03.2021	South Africa
MH182010	10.12.2014	Kenya
KX453394	19.04.2014	Kenya
OP744445	25.03.2022	Kenya
OR162211	18.12.2022	Kenya
OR162239	04.03.2022	Kenya
OP744443	22.03.2022	Kenya
OP744449	01.04.2021	Kenya
PQ638686	07.01.2023	USA
PQ638701	12.01.2023	USA
PQ762856	12.12.2023	France
PQ762847	04.12.2023	France
PQ762662	17.12.2023	France
PQ762855	12.12.2023	France
PQ763038	20.10.2023	France
PQ762962	11.12.2023	France

PQ762857	13.12.2023	France
MZ516118	27.12.2019	Netherlands
PQ762808	02.12.2023	France
PQ763119	01.01.2024	France
PP760414	31.12.2022	USA
PP203266	10.09.2022	USA
PP203262	10.09.2022	USA
OP320398	04.02.2020	Philippines
OP320399	09.03.2020	Philippines
PP681245	05.12.2022	USA
PP594810	23.11.2023	USA
OR872615	25.10.2022	USA
OR883003	19.03.2023	USA
PQ410470	31.12.2022	Belgium
PQ348887	21.12.2022	United Kingdom
PP969994	30.11.2022	Ireland
PP681257	07.12.2022	USA
OR795477	15.11.2022	Germany
PQ762800	26.11.2023	France
PP969972	28.10.2022	Ireland
PP969983	08.11.2022	Ireland
PQ348878	09.10.2022	United Kingdom
PQ348863	31.08.2022	United Kingdom
PQ348866	30.08.2022	United Kingdom
PQ348964	10.10.2022	United Kingdom
PQ348966	31.12.2022	United Kingdom
PQ348885	21.10.2022	United Kingdom
PQ348873	22.09.2022	United Kingdom
PP270238	07.11.2023	USA
PQ762818	25.11.2023	France
PQ737354	30.10.2023	France
PQ762631	30.10.2023	France
PQ762903	08.01.2024	France
PQ008873	03.03.2024	USA
PQ008875	25.02.2024	USA
OR601480	15.08.2023	USA
PQ638789	11.11.2023	USA
PP594805	25.11.2023	USA
PP342417	28.11.2023	USA

PQ762675	18.12.2023	France
PP454568	01.01.2023	Saudi Arabia
OR045924	01.01.2021	United Arab Emirates
OQ980512	31.12.2021	United Arab Emirates
OR162263	03.05.2022	Kenya
OR162277	30.08.2022	Kenya
OR162273	06.07.2022	Kenya
OR162248	10.03.2022	Kenya
OR162264	03.05.2022	Kenya
OR162243	09.03.2022	Kenya
OR162236	01.03.2022	Kenya
OR162241	07.03.2022	Kenya
OR162244	09.03.2022	Kenya
OR162240	06.03.2022	Kenya
OP744446	25.03.2022	Kenya
PP957752	05.02.2024	USA
PQ762524	15.05.2024	USA
PP957775	09.04.2024	USA
PP978515	23.02.2024	USA
PP973770	05.02.2024	USA
PP847389	20.01.2024	USA
PP847381	12.01.2024	USA
PP847384	12.01.2024	USA
PP847362	09.01.2024	USA
PP847365	08.01.2024	USA
PP830405	23.12.2023	USA
PP830402	21.12.2023	USA
PP830390	16.12.2023	USA
PP830382	10.12.2023	USA
PP830381	08.12.2023	USA
PP668166	30.11.2023	USA
PP594797	25.11.2023	USA
PP668162	20.11.2023	USA
PP504641	11.11.2023	USA
PP504648	09.11.2023	USA
PP386361	06.11.2023	USA
PP386363	05.11.2023	USA
PP504636	03.11.2023	USA

PP504637	03.11.2023	USA
PP386364	02.11.2023	USA
PP386352	28.10.2023	USA
PP386355	28.10.2023	USA
PP386353	28.10.2023	USA
PP386338	25.10.2023	USA
PP237795	19.10.2023	USA
PP237792	16.10.2023	USA
PP237787	05.10.2023	USA
PP237780	29.09.2023	USA
PP135034	25.09.2023	USA
PP135019	11.09.2023	USA
PP135016	30.08.2023	USA
PP135013	25.08.2023	USA
PP781381	03.01.2024	USA
PP270278	26.11.2023	USA
PP594815	04.12.2023	USA
PP668170	02.12.2023	USA
PP237794	13.10.2023	USA
PP504631	05.11.2023	USA
PP830406	24.12.2023	USA
PP847376	05.01.2024	USA
PP709454	03.01.2024	USA
PP709384	28.12.2023	USA
PP504627	03.11.2023	USA
PP594799	23.11.2023	USA
PP709452	01.01.2024	USA
PP504633	02.11.2023	USA
PP830393	14.12.2023	USA
PP709380	24.12.2023	USA
PP830400	22.12.2023	USA
PP830413	23.12.2023	USA
PP957744	01.02.2024	USA
PP910762	22.01.2024	USA
PP504626	04.11.2023	USA
PP386366	05.11.2023	USA
PP830403	24.12.2023	USA
PP830409	22.12.2023	USA
PQ762497	13.03.2024	USA

PP781380	07.02.2024	USA
PQ762484	14.01.2024	USA
PP237790	13.10.2023	USA
PP594798	21.11.2023	USA
PP386346	28.10.2023	USA
PP978510	22.02.2024	USA
PP504651	11.11.2023	USA
PP709459	02.01.2024	USA
PP504652	10.11.2023	USA
PP386348	28.10.2023	USA
PP386351	28.10.2023	USA
PP978509	22.02.2024	USA
PP237785	09.10.2023	USA
PP978511	22.02.2024	USA
PP709440	25.12.2023	USA
PP957768	22.03.2024	USA
PP504645	10.11.2023	USA
PP401811	03.11.2023	USA
PP237788	08.10.2023	USA
PP910774	28.01.2024	USA
PQ762503	16.03.2024	USA
PQ762482	13.01.2024	USA
PP910756	11.01.2024	USA
PP668168	30.11.2023	USA
PP386341	27.10.2023	USA
PP668176	16.12.2023	USA
PP847360	06.01.2024	USA
PP709394	04.01.2024	USA
PP386357	04.11.2023	USA
PP910772	27.01.2024	USA
PP978528	03.03.2024	USA
PP386358	04.11.2023	USA
PP504655	09.11.2023	USA
PP504635	06.11.2023	USA
PP386356	02.11.2023	USA
PQ762488	30.01.2024	USA
PQ762494	12.03.2024	USA
PP668173	19.12.2023	USA
PP978514	23.02.2024	USA

PP830391	16.12.2023	USA
PP847366	08.01.2024	USA
PQ762490	30.01.2024	USA
PP401813	09.11.2023	USA
PP830399	15.12.2023	USA
PP709376	21.12.2023	USA
PP237789	08.10.2023	USA
PP386342	27.10.2023	USA
PP386362	04.11.2023	USA
PP594807	24.11.2023	USA
PP957755	04.02.2024	USA
PP504656	14.11.2023	USA
PP594812	21.11.2023	USA
PP957746	02.02.2024	USA
PQ762492	28.01.2024	USA
PP910766	26.01.2024	USA
PQ788251	13.01.2024	USA
PQ788249	12.01.2024	USA
PP847371	10.01.2024	USA
PP709457	31.12.2023	USA
PQ762480	22.12.2023	USA
PP668164	04.12.2023	USA
PP504653	09.11.2023	USA
PP504629	04.11.2023	USA
PP386350	28.10.2023	USA
PP386339	27.10.2023	USA
PP386345	27.10.2023	USA
PP237791	12.10.2023	USA
PP237783	04.10.2023	USA
PP237781	01.10.2023	USA
PP957747	02.02.2024	USA
PP978513	23.02.2024	USA
MH388041	09.02.2015	Saudi Arabia
MK182718	11.01.2013	Saudi Arabia
OM256492	01.01.2018	
OR143190	30.11.2022	USA
OR522515	13.11.2022	USA
OP320383	14.09.2019	Philippines
PP970009	09.12.2022	Ireland

PQ763033	09.02.2024	France
PQ762968	20.11.2023	France
PQ762909	15.12.2023	France
PQ762854	11.12.2023	France
PQ762849	04.12.2023	France
PQ763018	30.12.2023	France
PQ763021	16.12.2023	France
PP508189	18.05.2023	Peru
PP969993	29.11.2023	Ireland
PP969960	27.11.2023	Ireland
PP970001	17.11.2023	Ireland
PQ762698	06.12.2023	France
PQ762700	08.12.2023	France
PP959049	12.01.2024	USA
PQ762739	30.11.2023	France
PQ762839	07.12.2023	France
PQ762842	31.12.2023	France
PQ762777	08.02.2024	France
PQ762781	04.12.2023	France
PQ763062	12.11.2023	France
PQ762720	29.10.2023	France
PQ762912	28.10.2023	France
PQ762780	05.12.2023	France
PQ762791	02.11.2023	France
PQ762687	18.11.2023	France
PQ762716	10.01.2024	France
PQ762874	28.11.2023	France
PQ762713	25.12.2023	France
PQ763053	21.12.2023	France
PQ762682	24.11.2023	France
PQ762793	26.11.2023	France
PQ762835	27.11.2023	France
PQ763134	05.01.2024	France
PQ762674	04.12.2023	France
PQ762830	12.12.2023	France
PQ762966	29.01.2024	France
PP969947	18.06.2022	Ireland
PP770464	15.01.2024	USA
PP709780	04.04.2022	Russia

OR522490	27.11.2022	USA
PP342421	03.12.2023	USA
PP342437	19.12.2023	USA
PP352358	01.10.2022	USA
OR883005	07.11.2022	USA
OR915744	05.11.2022	USA
OR915758	05.11.2022	USA
OR883000	15.10.2022	USA
PP903813	11.10.2022	USA
PP903817	01.10.2022	USA
OR872620	12.09.2022	USA
OR915728	03.11.2022	USA
OR915738	01.11.2022	USA
PQ638685	22.01.2023	USA
PQ117651	02.01.2024	USA
PP352329	01.10.2022	USA
PP709448	30.12.2023	USA
PP795158	02.01.2024	USA
OR872624	16.07.2022	USA
OR872599	10.09.2022	USA
OR872630	15.10.2022	USA
PQ638793	03.11.2023	USA
PP957777	05.04.2024	USA
PQ762514	21.04.2024	USA
PQ849791	02.12.2024	USA
PQ008878	12.02.2024	USA
PP781386	08.01.2024	USA
PP781408	29.02.2024	USA
PP270240	11.10.2023	USA
PP495850	11.01.2024	USA
PQ638795	07.11.2023	USA
PQ638713	14.10.2023	USA
PP970038	09.11.2023	Ireland
PQ834893	04.12.2023	Mexico
OQ024142	05.11.2022	USA
OR872639	27.05.2022	USA
OR882999	06.12.2022	USA
OQ024112	06.11.2022	USA
OQ024153	03.11.2022	USA

PP454565	22.06.2023	Saudi Arabia
PQ348896	18.10.2022	United Kingdom
OR143194	19.12.2022	USA
OR143221	18.12.2022	USA
OR143149	10.12.2022	USA
PQ349024	12.02.2023	United Kingdom
PQ762959	24.11.2023	France
PQ762670	26.10.2023	France
PQ762669	13.10.2023	France
PQ762747	28.10.2023	France
PQ762869	12.12.2023	France
PQ762867	03.12.2023	France
PQ762929	01.12.2023	France
PQ762963	09.12.2023	France
PQ762717	18.01.2024	France
PQ763019	27.12.2023	France
PQ762678	29.10.2023	France
PQ762788	01.01.2024	France
PQ762772	29.12.2023	France
PQ762967	19.11.2023	France
PQ762737	08.12.2023	France
PQ763036	21.03.2024	France
PQ762831	19.11.2023	France
PQ762660	15.01.2024	France
PQ762958	13.01.2024	France
PQ762657	02.01.2024	France
PQ762775	27.12.2023	France
PQ762654	22.12.2023	France
PQ762641	05.12.2023	France
PQ737362	26.11.2023	France
PQ737358	18.11.2023	France
PQ762714	26.12.2023	France
PQ762989	31.12.2023	France
PQ737368	09.12.2023	France
PQ762635	18.11.2023	France
PQ762770	21.12.2023	France
PQ762652	18.12.2023	France
PQ762636	20.11.2023	France
PQ763020	31.12.2023	France

PQ762647	12.12.2023	France
PQ737364	05.12.2023	France
PQ762648	13.12.2023	France
PQ737359	20.11.2023	France
PQ762650	14.12.2023	France
PQ762649	13.12.2023	France
PQ737365	05.12.2023	France
PQ763043	13.12.2023	France
PQ762642	05.12.2023	France
PQ737363	27.11.2023	France
PQ762640	27.11.2023	France
PQ762639	26.11.2023	France
PQ762645	09.12.2023	France
PP970053	22.10.2022	Ireland
PP970050	14.10.2022	Ireland
PP970057	07.11.2022	Ireland
PP970060	11.11.2022	Ireland
PQ348894	13.10.2022	United Kingdom
PQ349020	15.11.2022	United Kingdom
PQ349016	16.08.2022	United Kingdom
PQ348891	07.10.2022	United Kingdom
PQ349025	12.04.2023	United Kingdom
OR522478	23.10.2022	USA
PP203261	19.09.2022	USA
PQ638707	16.11.2022	USA
PQ638677	12.11.2022	USA
OR522486	12.10.2022	USA
PQ349011	30.07.2022	United Kingdom
PQ788230	08.11.2024	USA
PQ849802	19.08.2024	USA
OK500268	30.04.2021	France
PQ762760	16.12.2023	France
PQ762840	27.12.2023	France
PQ763026	27.11.2023	France
PQ762727	18.11.2023	France
OR872609	11.05.2022	USA
PP970006	16.11.2022	Ireland
OR872617	22.09.2022	USA
PP970015	12.11.2022	Ireland

OR872638	19.09.2022	USA
PP969954	26.09.2022	Ireland
PP969979	01.12.2022	Ireland
PP957779	04.04.2024	USA
PP973773	10.02.2024	USA
PP973766	06.02.2024	USA
PP973765	04.02.2024	USA
PP973763	03.02.2024	USA
PP709387	28.12.2023	USA
PP882669	28.11.2023	USA
PP386354	28.10.2023	USA
PP957774	03.04.2024	USA
PP973755	28.01.2024	USA
PP709378	23.12.2023	USA
PP973777	16.02.2024	USA
PP910761	20.01.2024	USA
PP709401	09.01.2024	USA
PP709399	05.01.2024	USA
PP934457	05.03.2024	USA
PP934462	17.03.2024	USA
PP973775	10.02.2024	USA
PP709393	01.01.2024	USA
PP830386	14.12.2023	USA
PP957753	02.02.2024	USA
PP709400	09.01.2024	USA
PP830380	06.12.2023	USA
PP934453	29.02.2024	USA
PP910754	14.01.2024	USA
PQ762515	24.04.2024	USA
PP973772	07.02.2024	USA
PP973774	10.02.2024	USA
PP830385	11.12.2023	USA
PP934454	29.02.2024	USA
PP973764	04.02.2024	USA
PP709371	19.12.2023	USA
PQ638702	26.02.2023	USA
PQ638727	23.02.2023	USA
OQ024131	05.11.2022	USA
OQ024147	03.11.2022	USA

PP508184	21.04.2023	Peru
PP508185	21.04.2023	Peru
PP508183	18.04.2023	Peru
OR882982	10.03.2023	USA
PP270273	08.12.2023	USA
PP781398	26.12.2023	USA
PQ763025	24.12.2023	France
PQ762811	28.11.2023	France
PQ762949	11.12.2023	France
PQ762665	01.12.2023	France
PQ763024	25.11.2023	France
PQ762812	16.12.2023	France
OQ024117	03.11.2022	USA
OQ171924	26.10.2022	USA
OR915740	10.11.2022	USA
OP890330	30.11.2022	USA
PP681258	12.12.2022	USA
OR872590	23.10.2022	USA
OR522531	03.01.2023	USA
OR601471	09.10.2022	USA
OR522530	03.01.2023	USA
OR975304	22.12.2022	USA
PP781393	30.01.2024	USA
PP882672	18.10.2023	USA
PP681266	13.12.2022	USA
PP681249	07.12.2022	USA
PP681246	07.12.2022	USA
PP681271	28.12.2022	USA
PP681282	27.11.2022	USA
PP681255	08.12.2022	USA
PP681244	29.11.2022	USA
PP957754	05.02.2024	USA
PP352383	01.12.2022	USA
OR872586	24.06.2022	USA
PQ638687	11.11.2022	USA
OR872635	25.10.2022	USA
PP352336	01.11.2022	USA
OR872594	11.07.2022	USA
PP795143	29.10.2023	USA

PP795150	15.11.2023	USA
PQ763121	12.12.2023	France
PQ638710	16.10.2022	USA
PP770462	14.01.2024	USA
PQ638712	09.08.2023	USA
PQ638785	23.11.2023	USA
PQ638794	03.11.2023	USA
OR795472	14.10.2022	Germany
PQ762806	30.11.2023	France
PQ762672	01.12.2023	France
PQ618068	12.07.2024	Panama
PQ618071	30.05.2024	Panama
PQ618070	28.06.2024	Panama
PQ618069	14.06.2024	Panama
PQ618074	27.06.2024	Panama
PQ618073	05.07.2024	Panama
PQ618072	05.07.2024	Panama
PP270269	18.12.2023	USA
PP978508	18.02.2024	USA
PP978531	06.03.2024	USA
PP530278	03.11.2023	USA
PQ618076	01.08.2023	Panama
PQ763044	12.02.2024	France
PP934467	24.03.2024	USA
PP508188	18.05.2023	Peru
PQ618075	11.12.2023	Panama
PQ618077	19.11.2023	Panama
PP969990	28.11.2023	Ireland
PP970031	29.10.2023	Ireland
PP970027	25.10.2023	Ireland
PP681259	30.11.2022	USA
PP969981	16.11.2022	Ireland
PQ349021	21.11.2022	United Kingdom
OQ261752	27.10.2022	Austria
PP969971	25.10.2022	Ireland
PP969964	03.10.2022	Ireland
PP969968	16.10.2022	Ireland
PP969965	11.10.2022	Ireland
PQ410473	10.11.2023	Belgium

PQ763082	20.12.2023	France
PQ762729	18.11.2023	France
PQ762955	06.11.2023	France
PQ762723	03.11.2023	France
PQ763148	29.12.2023	France
PQ762960	09.01.2024	France
OQ024134	14.11.2022	USA
OR522509	22.01.2023	USA
OR795476	07.11.2022	Germany
OR795474	03.11.2022	Germany
OR143215	01.12.2022	USA
PP681247	06.12.2022	USA
PP681265	12.12.2022	USA
OR522465	01.11.2022	USA
PQ763035	11.03.2024	France
PP270264	04.12.2023	USA
OR143134	31.01.2023	USA
PP957766	27.03.2024	USA
PP847390	23.01.2024	USA
PP957769	27.03.2024	USA
PQ638679	10.12.2022	USA
OP890312	31.10.2022	USA
PQ638728	07.10.2022	USA
PP970043	16.11.2023	Ireland
PQ594188	01.01.2023	China
PQ192486	17.09.2022	Thailand
PP270259	05.12.2023	USA
PQ117647	15.12.2023	USA
PP970016	31.10.2023	Ireland
PP969955	22.11.2023	Ireland
PP969996	20.11.2023	Ireland
OR840705	08.04.2023	China
PP504649	13.11.2023	USA
PQ066215	19.11.2023	USA
PP270245	20.11.2023	USA
PP781428	27.02.2024	USA
LC816571	08.08.2023	Japan
PQ788213	11.11.2024	USA
PP974157	17.12.2023	China

LC816569	14.08.2023	Japan
PQ762972	04.12.2023	France
PP270268	05.12.2023	USA
PQ788223	17.11.2024	USA
PQ192490	11.08.2023	Thailand
LC816570	31.08.2023	Japan
PQ192489	10.10.2023	Thailand
PQ390735	22.12.2023	USA
PP504639	13.11.2023	USA
PQ762998	12.01.2024	France
PQ762863	30.12.2023	France
PQ762957	26.12.2023	France
PQ763027	23.12.2023	France
PQ762621	22.11.2023	France
PQ737344	22.11.2023	France
OK500265	29.04.2021	France
OR522467	06.11.2022	USA
OR522459	25.10.2022	USA
OR522522	11.12.2022	USA
PQ638667	25.11.2022	USA
PP495849	09.01.2024	USA
PQ638698	08.11.2022	USA
PQ638787	22.11.2023	USA
PP495845	28.12.2023	USA
PQ638798	02.11.2023	USA
PP352348	05.10.2022	USA
PP352366	01.11.2022	USA
OR522528	31.01.2023	USA
OR522505	27.12.2022	USA
OR975299	27.12.2022	USA
PP969966	11.10.2022	Ireland
PP203264	31.12.2022	USA
PQ638664	24.11.2022	USA
PP681254	23.11.2022	USA
OP890317	22.08.2022	USA
OR143185	14.01.2023	USA
OR143192	29.12.2022	USA
OR522472	08.11.2022	USA
OP890336	01.11.2022	USA

OR882976	07.11.2022	USA
OR915768	22.11.2022	USA
OP890325	01.10.2022	USA
OR915753	15.12.2022	USA
OR143146	08.12.2022	USA
PP352370	01.11.2022	USA
PP681276	06.01.2023	USA
OR522469	06.11.2022	USA
OR522495	04.11.2022	USA
OQ024156	03.11.2022	USA
OP890334	01.11.2022	USA
OP890331	01.10.2022	USA
OP890327	01.10.2022	USA
OR522514	10.11.2022	USA
OR522476	10.11.2022	USA
PP795153	01.12.2023	USA
OR522511	10.01.2023	USA
OR872584	24.08.2022	USA
OP890335	01.11.2022	USA
OR522485	04.12.2022	USA
OR143156	16.12.2022	USA
OR915742	15.12.2022	USA
OR143200	17.01.2023	USA
OR162281	02.03.2023	Kenya
PP934449	26.02.2024	USA
PP882678	05.12.2023	USA
PP270261	11.12.2023	USA
PP970002	24.11.2023	Ireland
PP594802	24.11.2023	USA
PP969980	10.10.2022	Ireland
PP270254	25.10.2023	USA
PP957759	07.02.2024	USA
PQ618080	10.07.2024	Panama
OR795479	28.11.2022	Germany
PQ762894	05.01.2024	France
PQ763040	30.12.2023	France
PQ763100	29.12.2023	France
PQ762895	19.12.2023	France
PQ762984	13.12.2023	France

PQ762804	12.12.2023	France
PQ762893	08.12.2023	France
PQ762888	06.12.2023	France
PQ762900	04.12.2023	France
PQ762937	29.11.2023	France
PQ763166	28.11.2023	France
PQ763022	26.11.2023	France
PQ737348	19.11.2023	France
PQ762633	29.10.2023	France
PQ737355	28.10.2023	France
PQ762632	28.10.2023	France
PQ762907	27.10.2023	France
PQ737352	20.10.2023	France
PQ737350	19.10.2023	France
PQ762627	19.10.2023	France
PQ737351	19.10.2023	France
PQ763065	30.01.2024	France
PQ737356	29.10.2023	France
PQ762802	09.12.2023	France
PQ763142	07.11.2023	France
PQ762889	15.11.2023	France
PQ763081	20.11.2023	France
PQ762880	19.12.2023	France
PQ762629	20.10.2023	France
PQ762628	19.10.2023	France
PQ762885	04.12.2023	France
PQ762625	19.11.2023	France
PQ763169	30.12.2023	France
PQ762878	10.12.2023	France
PQ763125	15.11.2023	France
PQ762891	26.12.2023	France
PQ763103	07.01.2024	France
PQ618079	11.08.2023	Panama
OR522492	07.12.2022	USA
OR522484	02.12.2022	USA
OR872634	24.06.2022	USA
OR872611	24.08.2022	USA
OR915745	31.10.2022	USA
OR872613	13.10.2022	USA

PP781396	08.01.2024	USA
PP709391	03.01.2024	USA
OR915730	31.10.2022	USA
OR915751	02.10.2022	USA
OR872603	26.10.2022	USA
OR872595	15.07.2022	USA
PP969970	25.10.2022	Ireland
PP969977	11.10.2022	Ireland
PP969945	06.10.2022	Ireland
PP969946	06.10.2022	Ireland
PP969944	06.10.2022	Ireland
OR872593	16.10.2022	USA
OR872642	25.10.2022	USA
OR882993	27.11.2022	USA
OR883015	27.11.2022	USA
PQ638690	11.11.2022	USA
OR872591	27.10.2022	USA
OR872598	14.09.2022	USA
OR872633	11.05.2022	USA
OR882997	13.11.2022	USA
OR883006	01.05.2023	USA
PP903816	19.10.2022	USA
OR915739	17.12.2022	USA
OR915733	13.10.2022	USA
OR872631	08.07.2022	USA
OR872606	21.10.2022	USA
PP781404	21.12.2023	USA
PP781422	26.01.2024	USA
PP882676	21.11.2023	USA
PP681252	08.12.2022	USA
PQ834846	04.11.2022	Mexico
PQ834837	11.10.2022	Mexico
OR915750	28.11.2022	USA
OR143157	05.01.2023	USA
PP903833	11.04.2024	USA
PQ849803	10.08.2024	USA
PQ638705	16.11.2022	USA
PQ638694	11.11.2022	USA
OQ171905	26.10.2022	USA

PP910782	13.02.2024	USA
PP910781	13.02.2024	USA
PP352359	01.11.2022	USA
PP352335	01.10.2022	USA
PP352339	30.11.2022	USA
PP352387	01.12.2022	USA
PQ834886	21.11.2023	Mexico
PQ834885	21.11.2023	Mexico
PQ834882	13.11.2023	Mexico
PQ638700	11.11.2022	USA
OP890324	31.10.2022	USA
OR522482	06.12.2022	USA
PP760398	01.11.2022	USA
OP890320	01.11.2022	USA
OP890328	01.11.2022	USA
PP760404	01.12.2022	USA
OR522463	31.10.2022	USA
OR883016	11.11.2022	USA
PQ638691	11.11.2022	USA
PQ638665	24.11.2022	USA
PQ638671	24.11.2022	USA
PQ638660	10.10.2022	USA
PP178651	04.12.2022	USA
PQ638689	11.11.2022	USA
PP760416	12.10.2022	USA
OP890321	12.10.2022	USA
OR915747	11.10.2022	USA
OP890338	01.10.2022	USA
OP890318	01.10.2022	USA
PP970039	10.11.2023	Ireland
PP997255	01.04.2024	USA
PQ390721	19.12.2023	USA
PQ638729	05.09.2023	USA
PP530257	18.12.2023	USA
PP530271	19.12.2023	USA
PP530275	07.12.2023	USA
PQ638786	23.11.2023	USA
PP495847	04.01.2024	USA
OQ024127	03.11.2022	USA

OR143167	17.12.2022	USA
OQ171902	18.10.2022	USA
OR882996	17.04.2023	USA
OR522508	23.01.2023	USA
OR522513	15.01.2023	USA
PP352380	31.12.2022	USA
OR143151	10.12.2022	USA
PP352373	31.12.2022	USA
PQ638663	24.11.2022	USA
OR872601	26.10.2022	USA
OR143205	05.12.2022	USA
OQ024113	14.11.2022	USA
OQ171908	21.10.2022	USA
OQ024129	04.11.2022	USA
OQ024144	03.11.2022	USA
OP890333	01.11.2022	USA
OP965712	01.11.2022	USA
OR143150	21.12.2022	USA
OR143175	25.12.2022	USA
OR143172	05.12.2022	USA
PP760388	31.10.2022	USA
PP795155	11.12.2023	USA
PP270236	21.11.2023	USA
OR601478	03.11.2022	USA
PQ638711	16.10.2022	USA
OR522475	09.11.2022	USA
PP903834	20.10.2022	USA
OR915749	12.12.2022	USA
OR872582	25.10.2022	USA
OR883022	28.06.2023	USA
OR882980	14.11.2022	USA
PP903807	24.10.2022	USA
PP903806	30.11.2023	USA
PQ066225	01.12.2023	USA
PP903828	29.10.2022	USA
PP903822	06.10.2022	USA
OR882970	12.11.2022	USA
OR882995	28.02.2023	USA
OR882971	24.03.2023	USA

PP781399	17.12.2023	USA
OR883004	16.12.2022	USA
OR883008	14.12.2022	USA
PP903826	22.09.2022	USA
PQ066218	12.09.2022	USA
PP903829	17.09.2022	USA
PP903835	19.10.2022	USA
OR882973	07.11.2022	USA
OR915775	01.12.2022	USA
PP903825	22.11.2022	USA
OR522519	17.01.2023	USA
OR143147	09.12.2022	USA
OR143155	11.12.2022	USA
PP681278	10.01.2023	USA
OR522480	16.10.2022	USA
OR522483	05.12.2022	USA
OR522488	29.11.2022	USA
OR522489	29.11.2022	USA
OR143199	11.03.2023	USA
OR143173	17.12.2022	USA
OR143208	06.12.2022	USA
OR143219	01.12.2022	USA
OR143193	31.12.2022	USA
PP760392	01.10.2022	USA
OR143203	06.12.2022	USA
OR882979	23.11.2022	USA
OR883013	03.11.2022	USA
OR883001	28.10.2022	USA
PP903808	03.11.2022	USA
PP903820	26.10.2022	USA
PP903831	26.10.2022	USA
PP903832	26.10.2022	USA
OR882990	07.12.2022	USA
OR882987	27.11.2022	USA
OR882998	25.11.2022	USA
OR883021	22.11.2022	USA
PP903818	11.10.2022	USA
PP781423	15.02.2024	USA
PQ066223	18.12.2023	USA

PQ066226	18.12.2023	USA
PP903823	19.10.2022	USA
OR882984	27.11.2022	USA
OR915764	22.11.2022	USA
OR882994	04.11.2022	USA
PP781412	02.01.2024	USA
OR883011	13.12.2022	USA
PP903811	09.10.2022	USA
OR882977	20.11.2022	USA
OR915766	27.11.2022	USA
PP903810	20.03.2024	USA
PP781394	03.01.2024	USA
OR915759	10.11.2022	USA
OR915746	02.11.2022	USA
OR915757	20.10.2022	USA
OR882983	31.10.2022	USA
OR883019	31.10.2022	USA
OR915748	27.10.2022	USA
OR915736	01.01.2023	USA
OR915743	01.01.2023	USA
OR915761	01.01.2023	USA
PQ762506	19.03.2024	USA
PQ762500	14.03.2024	USA
PP978497	19.02.2024	USA
PP957757	07.02.2024	USA
PP748753	28.01.2024	USA
PP910770	27.01.2024	USA
PP847392	17.01.2024	USA
PP847375	06.01.2024	USA
PP709458	02.01.2024	USA
PP709389	31.12.2023	USA
PP709381	17.12.2023	USA
PP830396	14.12.2023	USA
PP594803	26.11.2023	USA
PP504640	11.11.2023	USA
PP386336	26.10.2023	USA
PP237782	02.10.2023	USA
PP830404	21.12.2023	USA
PQ762525	17.05.2024	USA

PP910765	25.01.2024	USA
PP978506	20.02.2024	USA
PP594800	24.11.2023	USA
PP830407	21.12.2023	USA
PQ762516	01.05.2024	USA
PP957770	28.03.2024	USA
PP910769	26.01.2024	USA
PP847385	21.01.2024	USA
PP401818	29.11.2023	USA
PP401810	05.11.2023	USA
PP504646	09.11.2023	USA
PP504638	05.11.2023	USA
PP978532	11.03.2024	USA
PP830411	25.12.2023	USA
PP594808	25.11.2023	USA
PP847372	04.01.2024	USA
PP957758	05.02.2024	USA
PP978524	29.02.2024	USA
PQ762489	30.01.2024	USA
PP934466	24.03.2024	USA
PP978500	19.02.2024	USA
PP847374	06.01.2024	USA
PP709455	04.01.2024	USA
PP709382	23.12.2023	USA
PP504642	09.11.2023	USA
PP957751	03.02.2024	USA
PP910763	25.01.2024	USA
PP709390	02.01.2024	USA
PP709383	25.12.2023	USA
PP957762	09.02.2024	USA
PP910777	31.01.2024	USA
PP978503	20.02.2024	USA
PP978502	16.02.2024	USA
PP973761	05.02.2024	USA
PQ762519	30.04.2024	USA
PQ762521	08.05.2024	USA
PQ762518	30.04.2024	USA
PQ762481	12.01.2024	USA
PP401812	07.11.2023	USA

PP978518	24.02.2024	USA
PP668175	18.12.2023	USA
PP830383	09.12.2023	USA
PP910784	11.02.2024	USA
PQ762493	13.03.2024	USA
PQ762485	14.01.2024	USA
PP594801	24.11.2023	USA
PP709377	27.12.2023	USA
PP709443	26.12.2023	USA
PQ788246	13.01.2024	USA
PP709388	28.12.2023	USA
PP830412	24.12.2023	USA
PP978533	11.03.2024	USA
PP709441	23.12.2023	USA
PP847373	10.01.2024	USA
PP957748	03.02.2024	USA
PP795154	07.12.2023	USA
PP270267	11.12.2023	USA
OR883009	19.06.2023	USA
PP795138	22.10.2023	USA
PP781401	28.12.2023	USA
PP957773	08.04.2024	USA
PP978512	22.02.2024	USA
PP957745	02.02.2024	USA
PP973751	15.01.2024	USA
PP709444	25.12.2023	USA
PP668174	19.12.2023	USA
PP504632	04.11.2023	USA
PP386347	27.10.2023	USA
PP237793	15.10.2023	USA
PP934450	29.02.2024	USA
PP934465	22.03.2024	USA
PP847367	09.01.2024	USA
PP401820	03.12.2023	USA
PP847394	21.01.2024	USA
PQ762510	19.03.2024	USA
PQ788248	12.01.2024	USA
PP934459	09.03.2024	USA
PP910778	13.02.2024	USA

PP910779	13.02.2024	USA
PP910775	28.01.2024	USA
PP709442	22.12.2023	USA
PP709449	03.01.2024	USA
PP978505	17.02.2024	USA
PP594811	25.11.2023	USA
PP973759	29.01.2024	USA
PP830397	14.12.2023	USA
PP847364	09.01.2024	USA
PP978520	25.02.2024	USA
PP978519	24.02.2024	USA
PP668169	05.12.2023	USA
PQ762502	17.03.2024	USA
PQ762496	14.03.2024	USA
PQ788253	14.01.2024	USA
PP401814	20.11.2023	USA
PP504634	06.11.2023	USA
PP594817	04.12.2023	USA
PP830392	14.12.2023	USA
OR143211	08.01.2023	USA
PP270233	20.11.2023	USA
PP270252	20.11.2023	USA
PP270248	01.11.2023	USA
PP270263	08.12.2023	USA
PP781392	29.12.2023	USA
PQ114111	29.12.2022	USA
PQ638683	27.12.2022	USA
PP781379	17.12.2023	USA
PP781421	17.01.2024	USA
OR872612	20.09.2022	USA
OR872597	16.10.2022	USA
PP795135	11.10.2023	USA
OQ024126	04.11.2022	USA
PQ638661	22.11.2022	USA
PP352365	01.11.2022	USA
OQ024145	14.11.2022	USA
PP352331	01.10.2022	USA
OR522466	05.11.2022	USA
OR143148	10.12.2022	USA

OR143145	08.12.2022	USA
OR143158	25.01.2023	USA
OR143169	03.12.2022	USA
OR143143	26.12.2022	USA
OR143163	04.02.2023	USA
PP270244	08.11.2023	USA
OR915767	16.09.2023	USA
PP781391	16.02.2024	USA
OR143160	25.01.2023	USA
OR143178	18.04.2023	USA
OR143196	08.12.2022	USA
PP910773	28.01.2024	USA
OR915776	17.07.2023	USA
PQ762513	20.04.2024	USA
PP342427	06.12.2023	USA
PP342428	06.12.2023	USA
PP342418	29.11.2023	USA
OR915754	02.11.2022	USA
OR143165	23.02.2023	USA
OR143164	22.02.2023	USA
OR143159	09.02.2023	USA
OR522525	29.12.2022	USA
OR143141	18.12.2022	USA
OR883020	13.04.2023	USA
OR143201	05.12.2022	USA
OR143180	30.11.2022	USA
OR143186	06.12.2022	USA
OR143176	16.12.2022	USA
OR143216	13.12.2022	USA
PP270260	15.11.2023	USA
PP978516	22.02.2024	USA
PP978504	17.02.2024	USA
PP709451	01.01.2024	USA
PP830401	25.12.2023	USA
PP830414	22.12.2023	USA
OR872614	04.11.2022	USA
OR872629	06.11.2022	USA
OR522500	20.12.2022	USA
PP681263	12.12.2022	USA

PP760401	01.11.2022	USA
PP681248	06.12.2022	USA
PP760396	30.11.2022	USA
PQ638708	16.11.2022	USA
OR522471	07.11.2022	USA
PP760408	01.01.2023	USA
OQ024110	07.11.2022	USA
OQ024140	03.11.2022	USA
OQ171925	12.10.2022	USA
OQ171929	12.10.2022	USA
OQ024146	04.11.2022	USA
OQ024151	04.11.2022	USA
PP296424	02.12.2022	USA
PP278016	30.11.2022	USA
PP352372	01.11.2022	USA
PP352343	01.11.2022	USA
OR915771	27.11.2022	USA
OR143153	09.12.2022	USA
PQ788242	27.10.2024	USA
PP973756	01.02.2024	USA
PP973767	06.02.2024	USA
PP781395	18.01.2024	USA
PP781400	11.01.2024	USA
PQ762486	15.01.2024	USA
PQ788240	31.10.2024	USA
PP270235	02.11.2023	USA
PQ834894	04.12.2023	Mexico
PQ834891	27.11.2023	Mexico
PQ834898	05.12.2023	Mexico
PQ834884	15.11.2023	Mexico
PQ834892	28.11.2023	Mexico
OR872592	27.10.2022	USA
OP890313	24.10.2022	USA
OP890326	24.10.2022	USA
PP781427	15.01.2024	USA
PP830384	10.12.2023	USA
PQ762754	27.11.2023	France
PP910753	11.01.2024	USA
PP973768	05.02.2024	USA

PP973762	03.02.2024	USA
PQ762499	14.03.2024	USA
PQ008874	05.03.2024	USA
PP910755	12.01.2024	USA
PP847368	09.01.2024	USA
PP709372	25.12.2023	USA
PP504643	10.11.2023	USA
PP903819	08.01.2024	USA
PP903824	08.01.2024	USA
PP934455	28.02.2024	USA
PQ788203	26.11.2024	USA
PQ788225	17.11.2024	USA
PQ849790	01.12.2024	USA
PQ849783	09.12.2024	USA
PQ849784	08.12.2024	USA
PQ849772	07.12.2024	USA
OQ171913	28.10.2022	USA
OR915729	07.01.2023	USA
OQ171912	12.07.2022	USA
PQ762743	10.10.2023	France
OR143135	11.02.2023	USA
PP203265	10.10.2022	USA
PP830379	06.12.2023	USA
PP795139	26.10.2023	USA
PP795141	26.10.2023	USA
PP795146	06.11.2023	USA
PP795144	30.10.2023	USA
PP795132	05.10.2023	USA
OR882989	04.11.2022	USA
PP352374	30.11.2022	USA
OR143152	14.12.2022	USA
PP760412	31.12.2022	USA
OR143174	30.11.2022	USA
OR143136	20.12.2022	USA
OR143217	11.12.2022	USA
OR795473	20.10.2022	Germany
OR143191	04.12.2022	USA
OR143161	24.01.2023	USA
OR143181	02.12.2022	USA

OR522532	04.01.2023	USA
PP681256	08.12.2022	USA
PP760387	26.11.2022	USA
OP890323	26.11.2022	USA
OR915762	26.12.2022	USA
OR915731	11.12.2022	USA
OR915755	24.10.2022	USA
OR915763	11.12.2022	USA
OP890329	01.11.2022	USA
PQ638669	23.11.2022	USA
PP178652	25.11.2022	USA
OP890339	01.10.2022	USA
PP760399	01.11.2022	USA
PP781411	09.01.2024	USA
OR143214	04.01.2023	USA
OR883012	17.12.2022	USA
PQ834851	23.11.2022	Mexico
OR872643	18.09.2022	USA
OQ171923	21.10.2022	USA
PQ834850	24.10.2022	Mexico
OQ171930	18.10.2022	USA
OQ171903	17.10.2022	USA
OQ171898	31.10.2022	USA
PP352332	01.10.2022	USA
OQ024118	04.11.2022	USA
OQ024132	02.11.2022	USA
PP352360	30.11.2022	USA
PP352328	31.10.2022	USA
OQ024133	03.11.2022	USA
PQ834873	18.11.2022	Mexico
PP795152	27.11.2023	USA
PQ834889	24.11.2023	Mexico
PP594809	24.11.2023	USA
PP847383	11.01.2024	USA
PP709456	03.01.2024	USA
PP934464	18.03.2024	USA
PP681279	02.12.2022	USA
OP890332	17.10.2022	USA
OR143162	29.01.2023	USA

OR143182	16.01.2023	USA
PP882670	19.12.2023	USA
PP795163	24.01.2024	USA
PQ834903	13.12.2023	Mexico
PQ762504	17.03.2024	USA
PQ762501	16.03.2024	USA
PP973776	14.02.2024	USA
OR915741	02.10.2022	USA
PP795164	09.02.2024	USA
OR915752	16.10.2022	USA
OQ024130	03.11.2022	USA
OQ171928	25.10.2022	USA
PQ834877	06.12.2022	Mexico
PP270258	06.12.2023	USA
PP770453	27.02.2024	USA
PP770470	29.01.2024	USA
PP781424	31.01.2024	USA
PQ618078	28.07.2023	Panama
PQ834896	01.12.2023	Mexico
PP270246	06.11.2023	USA
PP504650	11.11.2023	USA
PP830394	14.12.2023	USA
PQ762985	10.12.2023	France
PQ762715	04.01.2024	France
PQ763023	24.12.2023	France

References

1. Jukes, T. H. & Cantor, C. R. Chapter 24 - Evolution of protein molecules. in *Mammalian Protein Metabolism* (ed. Munro, H. N.) 21–132 (Academic Press, 1969). doi:10.1016/B978-1-4832-3211-9.50009-7.
2. Stadler, T. *et al. Decoding Genomes: From Sequences to Phylodynamics*. (ETH Zurich, 2024). doi:10.3929/ethz-b-000664449.
3. Jang, J. & Bae, S.-E. Comparative co-evolution analysis between the HA and NA genes of influenza A virus. *Virology* **9**, 1178122X18788328 (2018).
4. Yu, J.-M., Fu, Y.-H., Peng, X.-L., Zheng, Y.-P. & He, J.-S. Genetic diversity and molecular evolution of human respiratory syncytial virus A and B. *Sci. Rep.* **11**, 12941 (2021).
5. Hadfield, J. *et al.* Nextstrain: Real-time tracking of pathogen evolution. *Bioinformatics* **34**, 4121–4123 (2018).
6. Bürki, C. *et al.* Characterizing co-circulating respiratory virus genomic diversity in Switzerland with hybrid-capture sequencing and phylogenetic reconstructions: Insights into the 2023/24 season. 2025.07.21.664397 Preprint at doi.org/10.1101/2025.07.21.664397 (2025).
